# Supplementary material for: Deep learning-based transformation of H&E stained tissues into special stains
Source: Nat Commun. 2021 Aug 12;12:4884. doi: 10.1038/s41467-021-25221-2 (PMC8361203; doi:10.1038/s41467-021-25221-2)
Supplement: Supplementary file 1 — Supplementary Information [file 41467_2021_25221_MOESM1_ESM.pdf]

Supplementary information for

Deep learning-based transformation of H&E stained tissues into special stains

Kevin de Haan[1,2,3], Yijie Zhang[1,2,3], Jonathan E. Zuckerman[4], Tairan Liu[1,2,3], Anthony E. Sisk[4], Miguel F. P. Diaz[5], Kuang-Yu Jen[6], Alexander Nobori[4], Sofia Liou[4], Sarah Zhang[4], Rana Riahi[4], Yair Rivenson[1,2,3], W. Dean Wallace[7], Aydogan Ozcan[1,2,3,8]

[1]Electrical and Computer Engineering Department, University of California, Los Angeles, CA, USA

[2]Bioengineering Department, University of California, Los Angeles, CA, USA

[3]California NanoSystems Institute (CNSI), University of California, Los Angeles, CA, USA

[4]Department of Pathology and Laboratory Medicine, David Geffen School of Medicine, University of California, Los Angeles, Los Angeles, CA, USA.

[5]Kaiser Permanente Los Angeles Medical Center, Department of Pathology, Los Angeles, CA, USA.

[6]Department of Pathology and Laboratory Medicine, University of California at Davis, Sacramento, CA, USA

[7]Department of Pathology and Laboratory Medicine, Keck School of Medicine of USC, Los Angeles, CA, USA

[8]Department of Surgery, David Geffen School of Medicine, University of California, Los Angeles, CA, USA

Corresponding authors: Yair Rivenson; rivensonyair@g.ucla.edu
W. Dean Wallace; william.wallace@med.usc.edu
Aydogan Ozcan; ozcan@ucla.edu

Supplementary Table 1. *Quality comparison between the stains generated by the stain transformation network and the histochemically stained tissue, where 4 is Perfect, 3 is Very Good, 2 is Good Enough (Passable), and 1 is Not Acceptable. The scores are the average of 16 fields-of view coming from 3 tissue sections, each rated twice by three separate pathologists.*

a) Masson's Trichrome

	Stain quality score	Nuclear detail	Cytoplasmic detail	Extracellular Fibrosis
Stain transformation	3.19	3.39	3.24	3.11
Histologically stained	3.09	2.95	3.19	3.30
Stain transformation std. error (between pathologists)	0.52	0.35	0.47	0.71
Std. error histological (between pathologists)	0.21	0.27	0.25	0.43

b) PAS

	Stain quality score	Nuclear detail	Cytoplasmic detail	Basement membrane detail
Stain transformation	3.40	3.53	3.38	3.39
Histologically stained	3.51	3.49	3.41	3.53
Stain transformation std. error (between pathologists)	0.41	0.26	0.39	0.44
Std. error histological (between pathologists)	0.33	0.33	0.42	0.33

c) Jones Silver Stain

	Stain quality score	Nuclear detail	Cytoplasmic detail	Basement membrane detail
Stain transformation	3.84	3.70	3.70	3.91
Histologically stained	3.88	3.72	3.82	3.98
Stain transformation std. error (between pathologists)	0.13	0.22	0.15	0.05
Std. error histological (between pathologists)	0.06	0.16	0.01	0.02

Supplementary Table 2. *Original diagnoses for the samples used to train the virtual staining and stain transformation neural networks.*

Sample number	Original diagnosis
Training sample 1	ACR 1A with CA-ACR grade 1A
Training sample 2	ACR 1A
Training sample 3	Active AMR, ACR 1A, TMA, and early membranous nephropathy
Training sample 4	Active AMR, ACR 2A
Training sample 5	Chronic active AMR, ACR 1B
Training sample 6	BK virus nephropathy
Training sample 7	Chronic active AMR
Training sample 8	ATN
Training sample 9	Active AMR, ACR 1A, TMA, and early membranous nephropathy
Training sample 10 (H&E only)	ATN
Training sample 11 (PAS, MT, Jones)	ATN
Training sample 12 (PAS only)	Active AMR
Validation sample 1	Active AMR, ACR 2B, TMA
Validation sample 2	Active AMR and Borderline changes
Validation sample 3	ATN

Supplementary Table 3. *Original diagnoses and the number of 1424×1424-pixel image patches for the samples used to train the style transfer CycleGAN neural networks.*

Sample number	Original diagnosis	Number of patches
Augmentation sample 1	ACR 1B	153
Augmentation sample 2	ACR 1A	139
Augmentation sample 3	Borderline changes	105
Augmentation sample 4	Acute GN	98
Augmentation sample 5	Crecentic lupus	99
Augmentation sample 6	MPGN	86
Augmentation sample 7	Diabetes	199
Augmentation sample 8	ATN	274

Abbreviations: ACR = acute cellular rejection, AMR = acute antibody-mediated rejection, TMA = thrombotic microangiopathy, GN = glomerulonephritis, MPGN = membranoproliferative glomerulonephritis.

Supplementary Table 4. *Diagnosis worksheet template.*

<u>KIDNEY BIOPSY EVALUATION WORKSHEET # (1 through 16)</u>

LINK TO H&E IMAGE (web link)

REVIEWER:

DATE:

HISTORY:

PRELIMINARY DIAGNOSIS:

(please check stains reviewed: ☐ H&E ☐ vPAS ☐ vMT ☐ vJMS)

Chronicity:

MICROSCOPIC EXAM:

Cores: _

Components:

Cortex ☐

Medulla ☐

Capsule ☐

Other ☐ ____

Number of glomeruli (completely sclerotic): _ (_)

Number of arteries: _

COMMENTS:

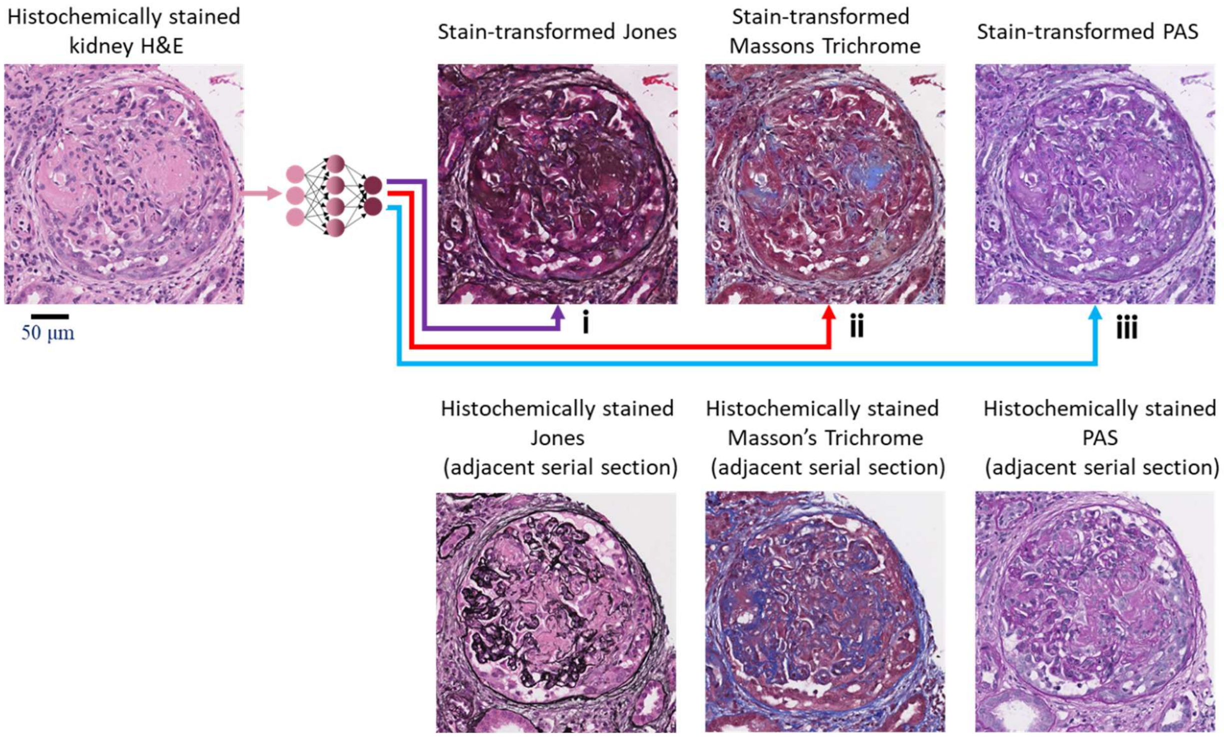

Supplementary Figure **1***: Example of the discordance demonstrated between the H&E and stain-transformed special stains for case # 3. The amyloid is darker than normal in the stain-transformed JMS stain for this field of view. (i) Generation of JMS. (ii) Generation of MT. (iii) Generation of PAS. A total of 58 cases were viewed by 3 pathologists to perform the statistical analysis.*

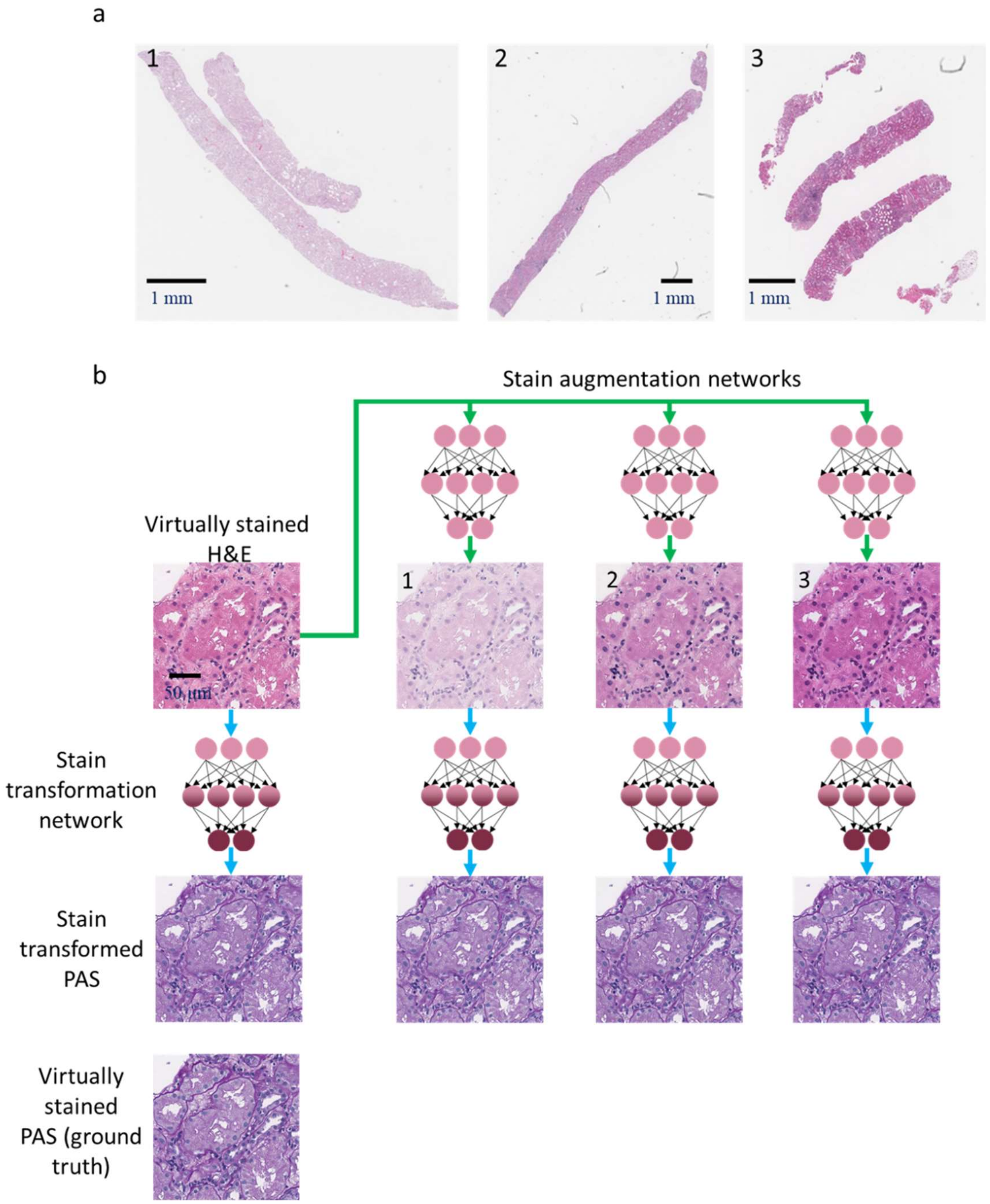

***Supplementary* Figure 2:** *Demonstration of the use of style transfer-based image data augmentation. a) Three H&E WSIs that are used as the ground truth distributions for three different stain augmentation networks. b) Demonstration of the effectiveness of the stain transformation technique across different H&E distributions. The three stain augmentation networks (green arrows) perform a style transfer to make the H&E field of view match the distribution of the corresponding three distributions shown in part a. This demonstrates that the stain transformation network (blue arrows) is robust and able to consistently perform accurate transformations when applied to various distributions. A total of 8 style augmentation networks corresponding to 8 unique H&E slides were used to train the stain transformation network.*

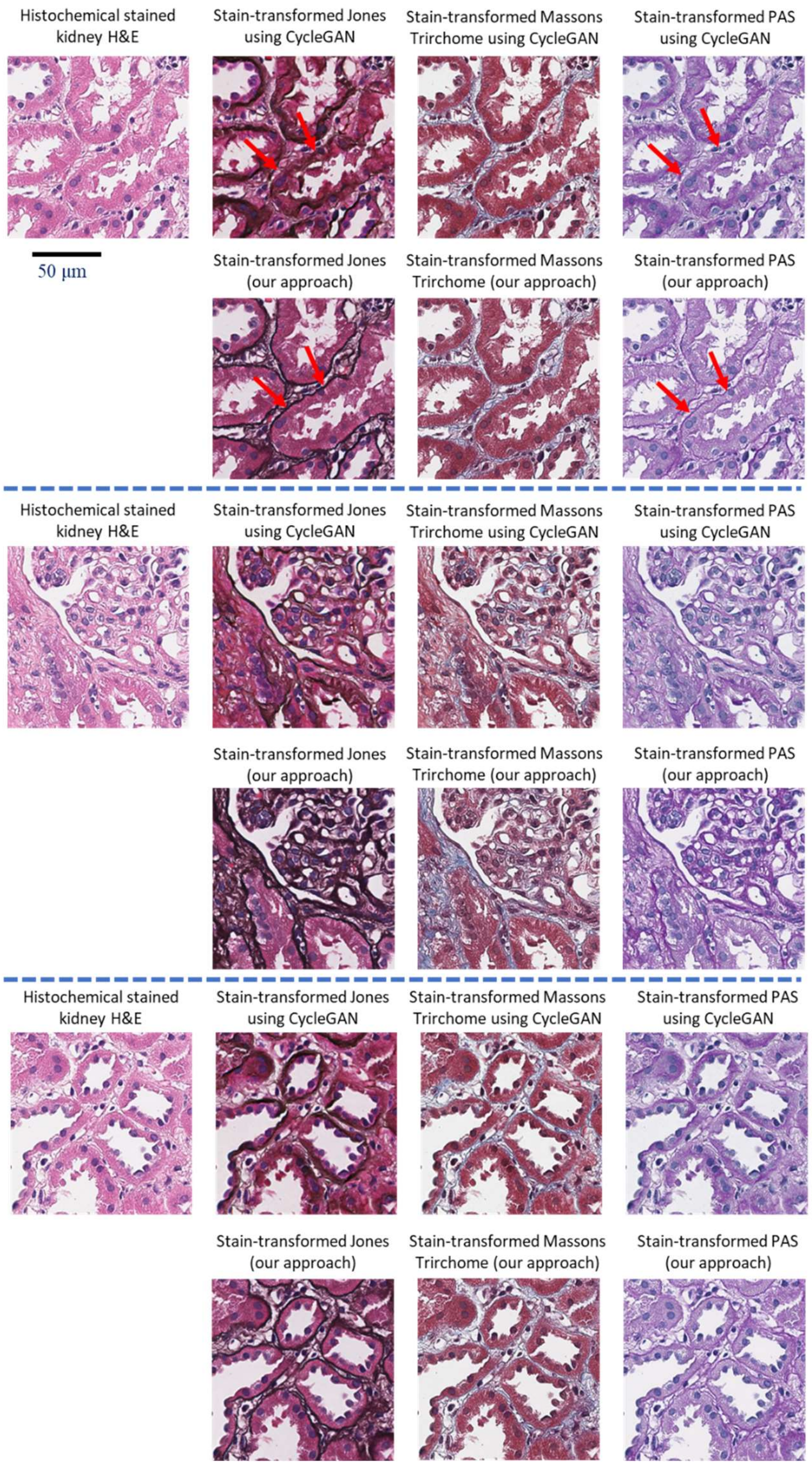

Supplementary Figure 3: *Comparison between the performance of the stain-transformation network presented in this work and stain transformations performed by a CycleGAN. For the Masson's Trichome stain there are only minor differences between the quality of the CycleGAN and our method. However, the other two stains are much more difficult for a CycleGAN to perform, and the CycleGAN technique hallucinates features throughout all of the images generated for these stains. For example, the red arrows point to locations where the basement membrane has been incorrectly labeled by the CycleGAN for the Jones and PAS stains.*

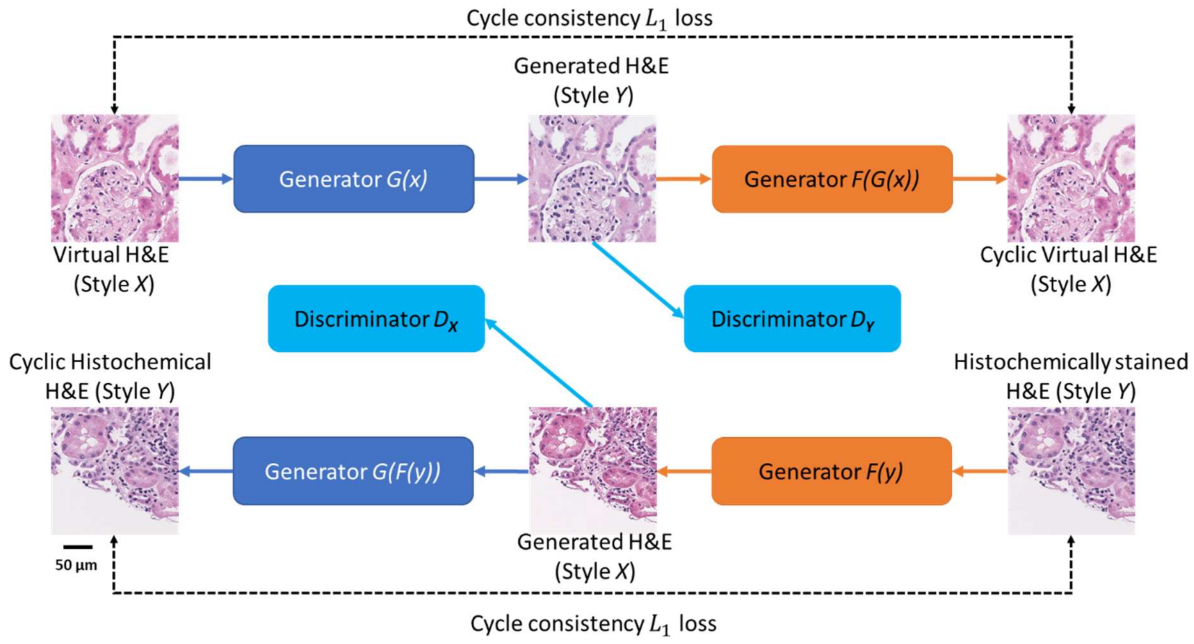

***Supplementary Figure* 4**: *Diagram showing the transformations performed by the various networks during the training phase (see the Methods section of the main text).*
