# Supplementary material for: Deep learning-based transformation of H&E stained tissues into special stains
Source: Nat Commun. 2021 Aug 12;12:4884. doi: 10.1038/s41467-021-25221-2 (PMC8361203; doi:10.1038/s41467-021-25221-2)
Supplement: Supplementary file 3 — Description of Additional Supplementary Files [file 41467_2021_25221_MOESM3_ESM.pdf]

Description of Additional Supplementary Files

Deep learning-based transformation of H&E stained tissues into special stains

File Name: Supplementary Data 1

Description:

Sheet 1: Adjudication report comparing the diagnoses made with H&E only against the use of both H&E and the stain transformed special stains.

Sheet 2: Adjudication report comparing the diagnoses made with H&E only against the use of both H&E and the histochemically stained special stains coming from serial tissue sections.

Sheets 3-6: The raw diagnostic information given by each pathologist for each of the three phases of the study.

File Name: Supplementary Data 2

Description:

Full set of images used for the stain quality assessment study, along with the text boxes used by the pathologists to rate the various aspects of each image. The images viewed by the pathologists were randomized and duplicated.
