# Supplementary material for: Deep learning-based transformation of H&E stained tissues into special stains
Source: Nat Commun. 2021 Aug 12;12:4884. doi: 10.1038/s41467-021-25221-2 (PMC8361203; doi:10.1038/s41467-021-25221-2)
Supplement: Supplementary file 5 — Supplementary Data 2 [file 41467_2021_25221_MOESM5_ESM.pdf]

Images used for stain quality assessment

This file contains the full set of images used for the stain quality assessment study, along with the text boxes used by the pathologists to rate the various aspects of each image. The images viewed by the pathologists were randomized and duplicated.

Masson’s Trichrome

Stain-transformed image #1

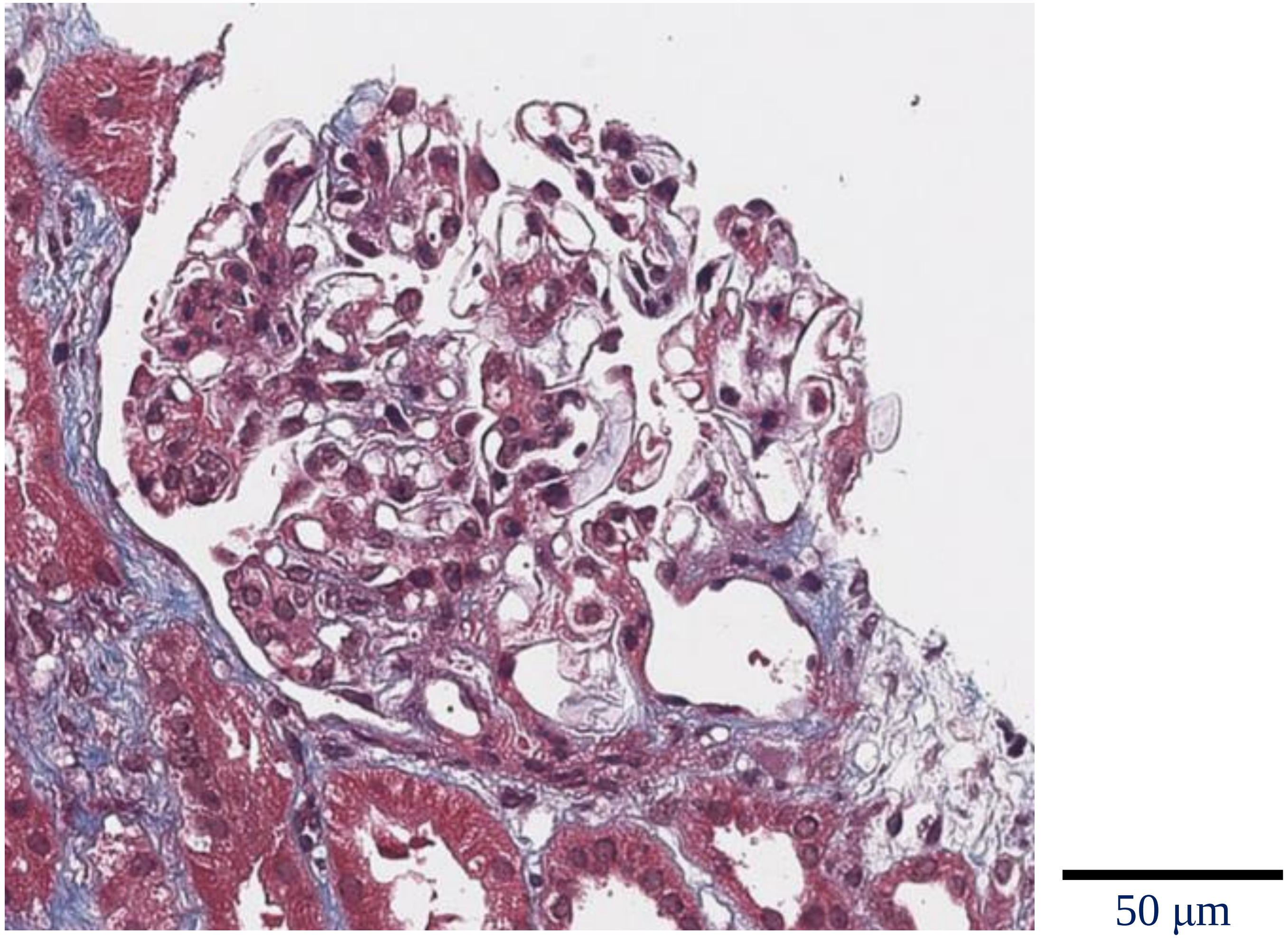

4 – Perfect
3 – Very good
2 – Good enough
1 – Not acceptable

	Stain quality score	Nuclear detail	Cytoplasmic detail	Extracellular Fibrosis
Score				

Histochemically stained image #1

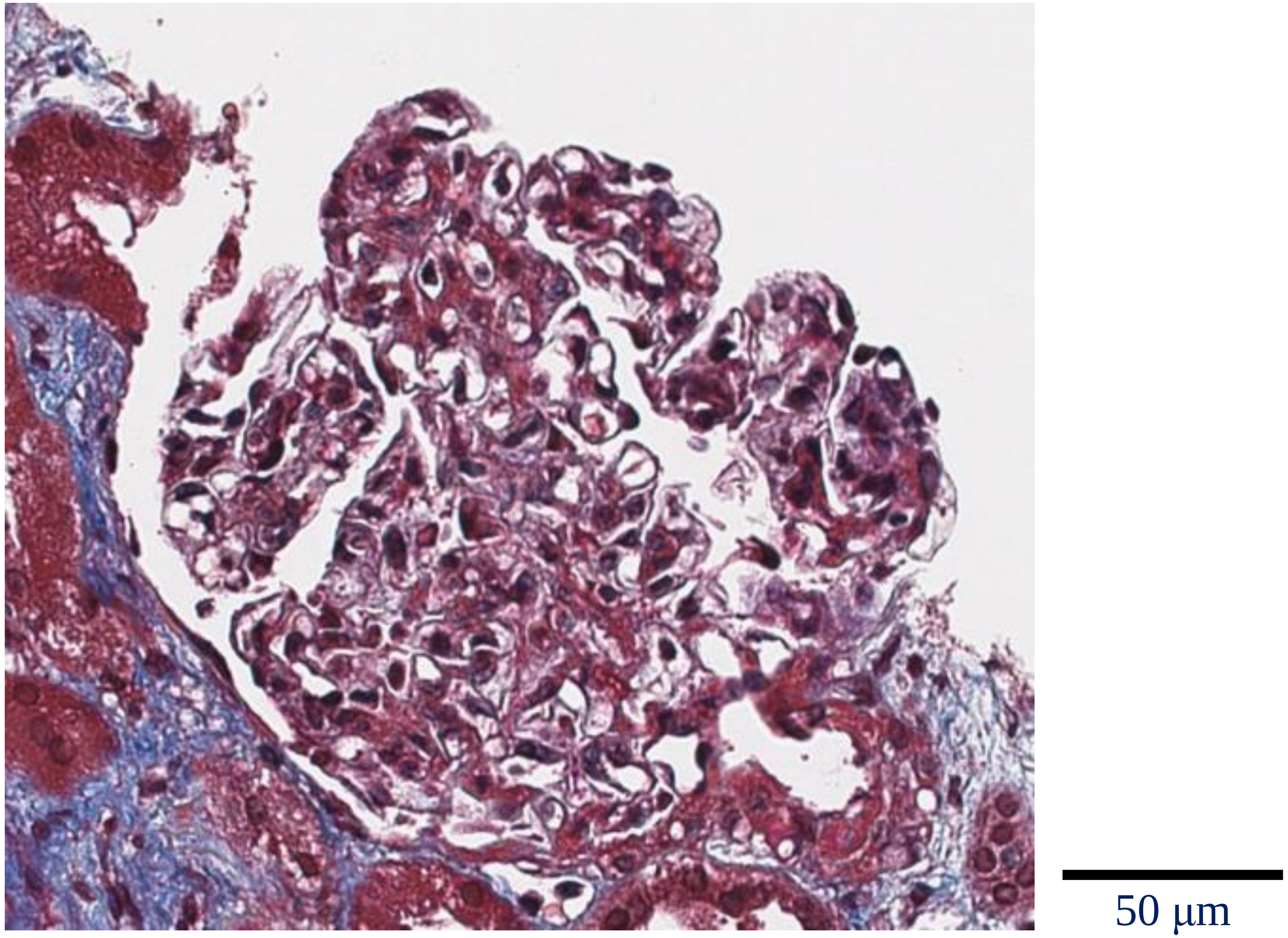

4 – Perfect
3 – Very good
2 – Good enough
1 – Not acceptable

	Stain quality score	Nuclear detail	Cytoplasmic detail	Extracellular Fibrosis
Score				

Stain-transformed image #2

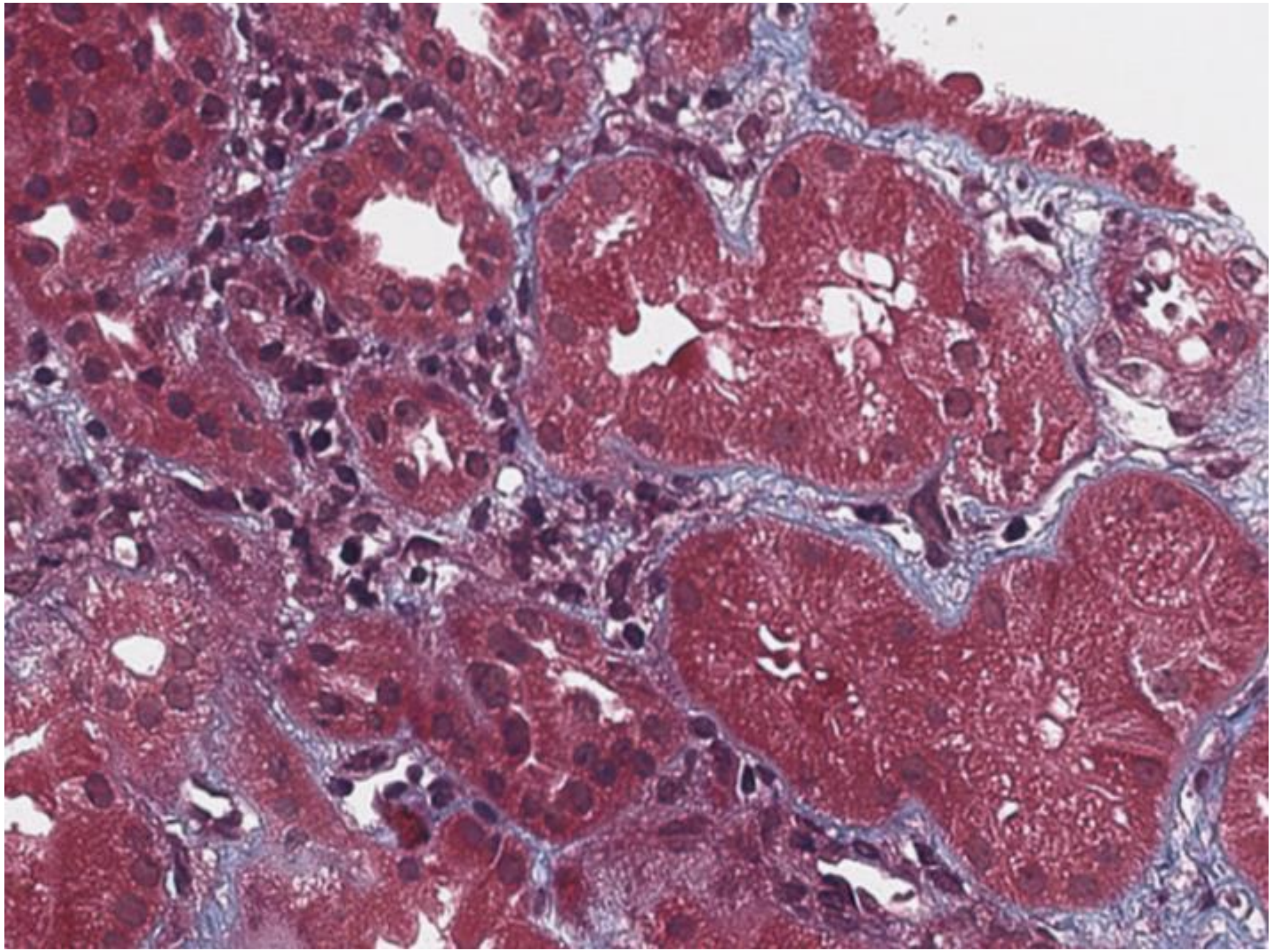

50 µm

4 – Perfect
3 – Very good
2 – Good enough
1 – Not acceptable

	Stain quality score	Nuclear detail	Cytoplasmic detail	Extracellular Fibrosis
Score				

Histochemically stained image #2

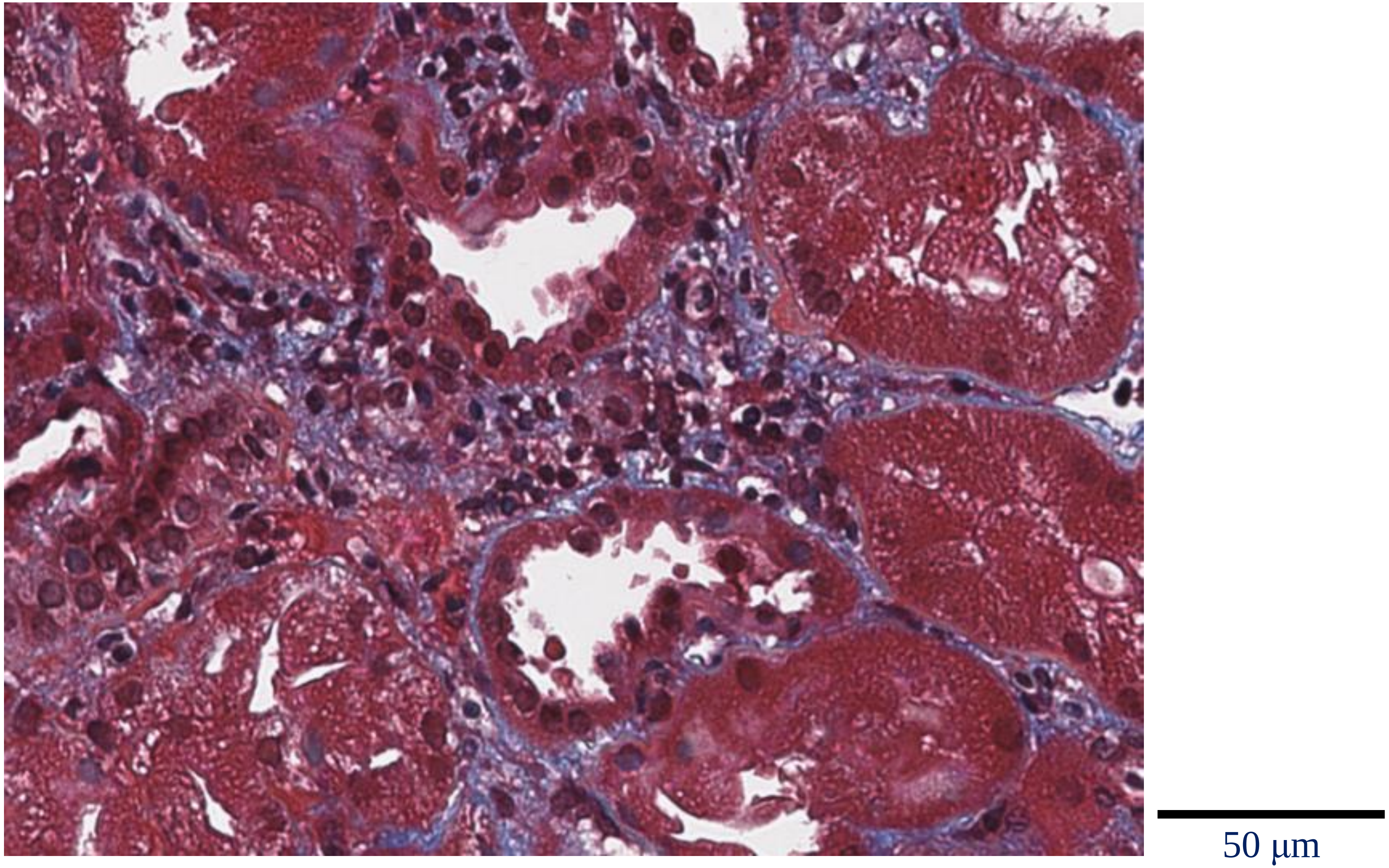

4 – Perfect
3 – Very good
2 – Good enough
1 – Not acceptable

	Stain quality score	Nuclear detail	Cytoplasmic detail	Extracellular Fibrosis
Score				

Stain-transformed image #3

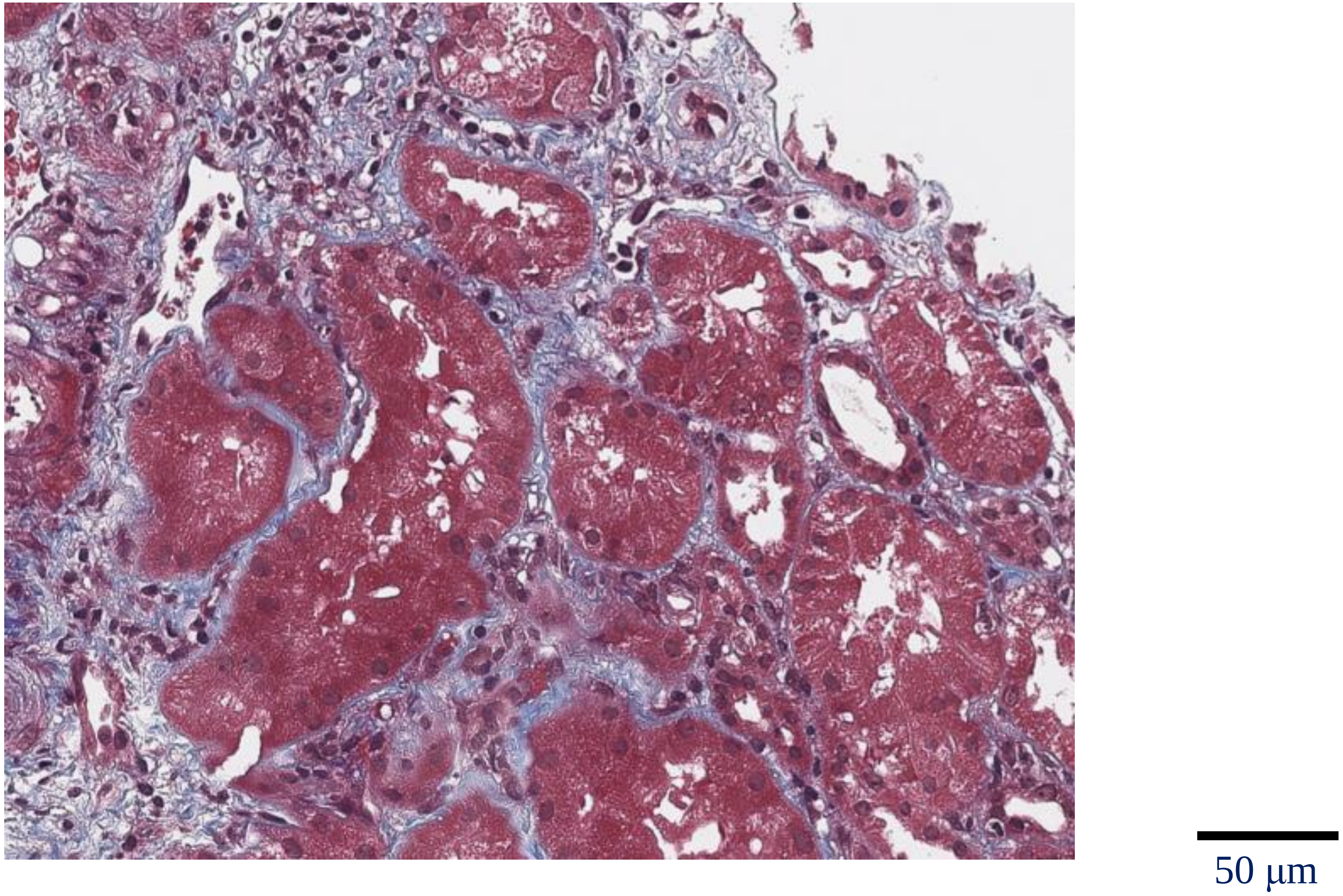

4 – Perfect
3 – Very good
2 – Good enough
1 – Not acceptable

	Stain quality score	Nuclear detail	Cytoplasmic detail	Extracellular Fibrosis
Score				

Histochemically stained image #3

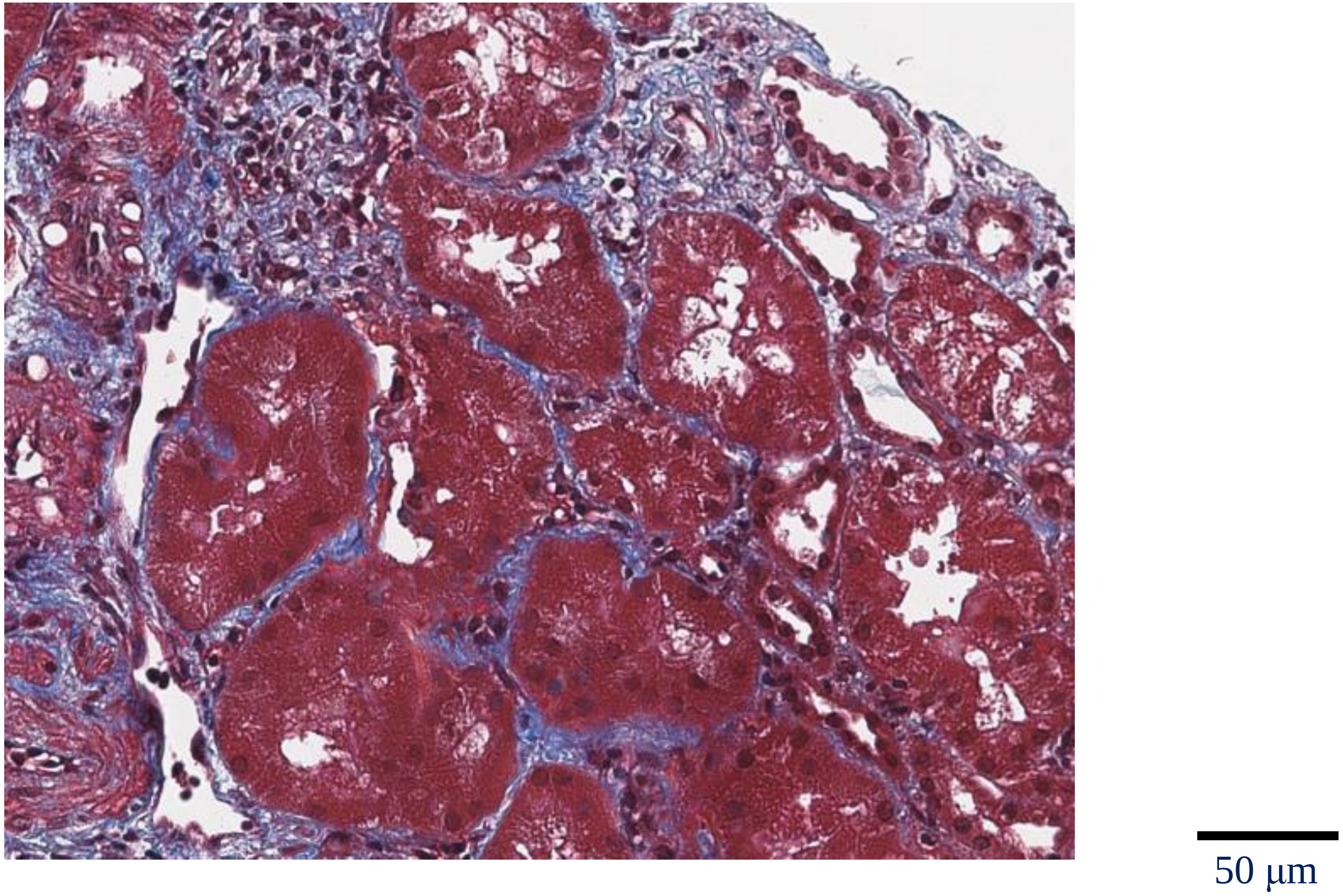

4 – Perfect
3 – Very good
2 – Good enough
1 – Not acceptable

	Stain quality score	Nuclear detail	Cytoplasmic detail	Extracellular Fibrosis
Score				

Stain-transformed image #4

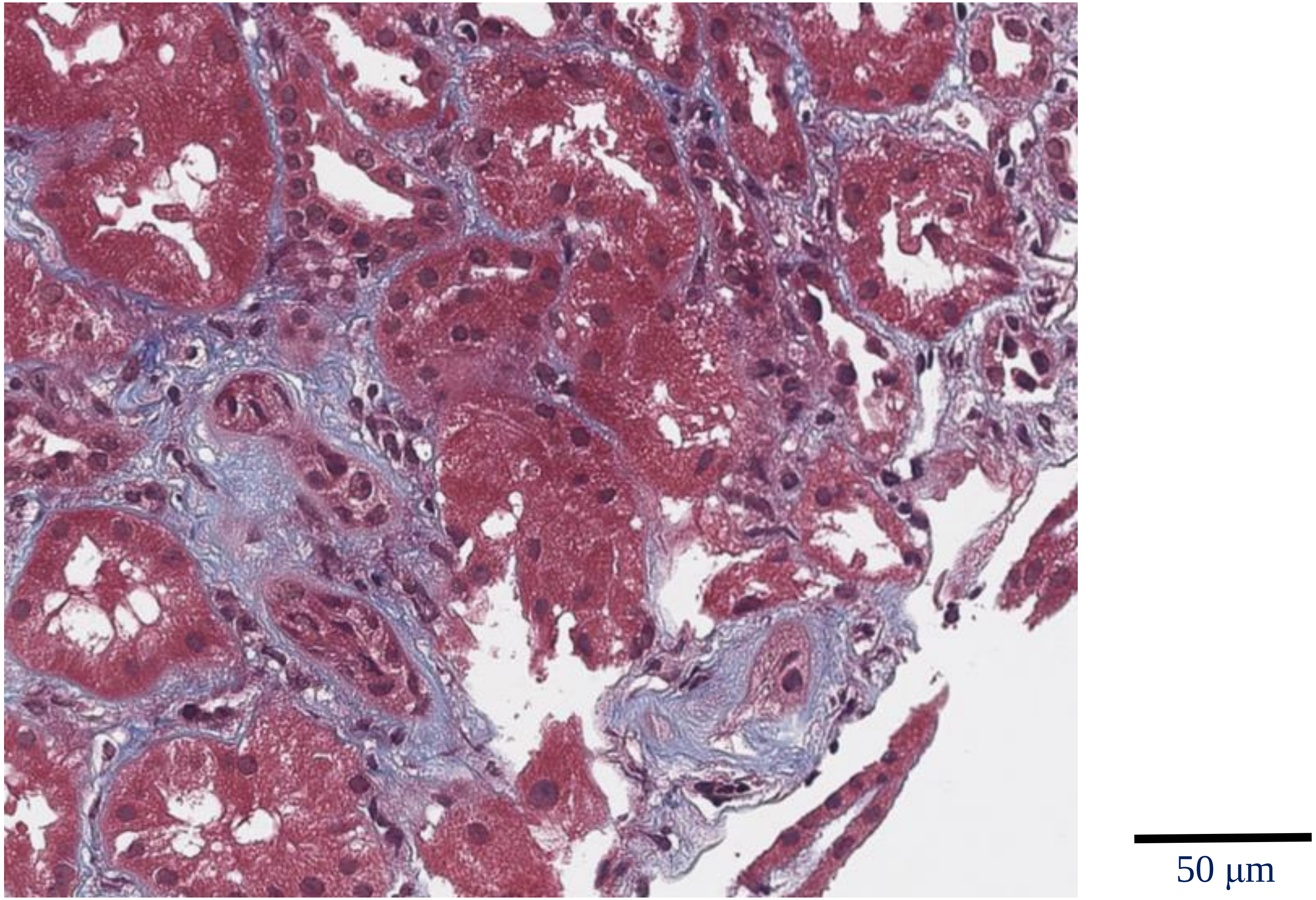

4 – Perfect
3 – Very good
2 – Good enough
1 – Not acceptable

	Stain quality score	Nuclear detail	Cytoplasmic detail	Extracellular Fibrosis
Score				

Histochemically stained image #4

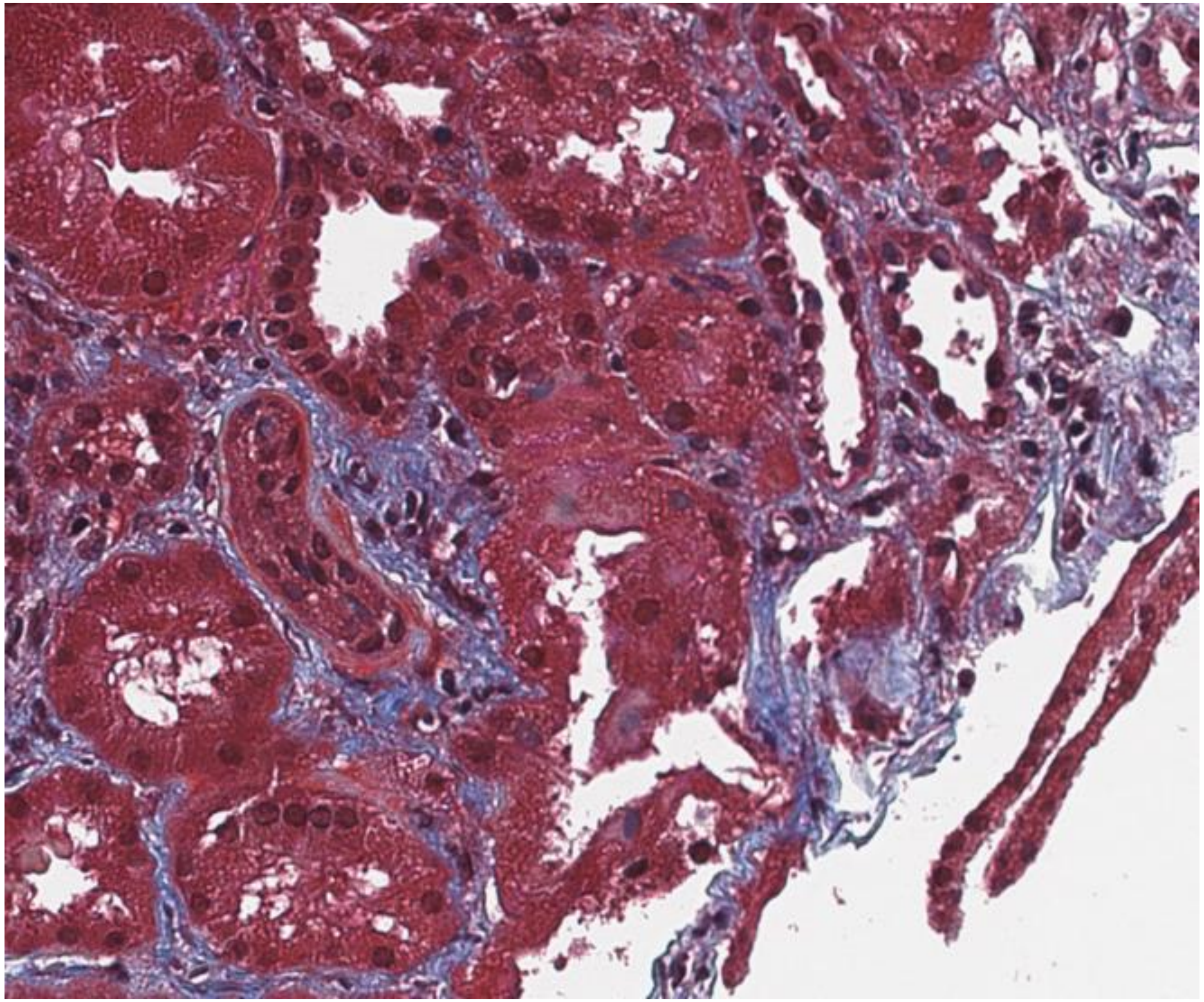

50 µm

4 – Perfect
3 – Very good
2 – Good enough
1 – Not acceptable

	Stain quality score	Nuclear detail	Cytoplasmic detail	Extracellular Fibrosis
Score				

Stain-transformed image #5

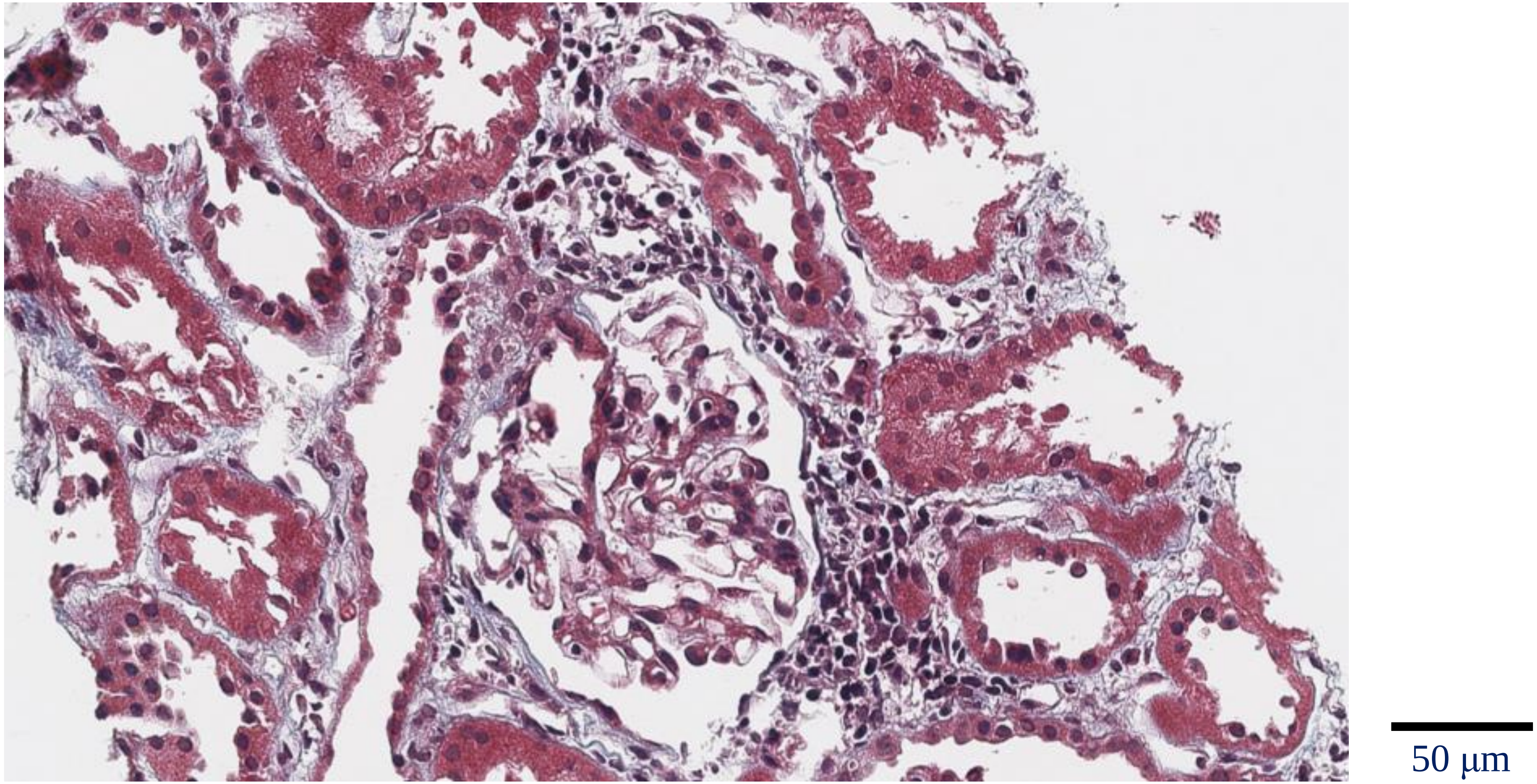

4 – Perfect
3 – Very good
2 – Good enough
1 – Not acceptable

	Stain quality score	Nuclear detail	Cytoplasmic detail	Extracellular Fibrosis
Score				

Histochemically stained image #5

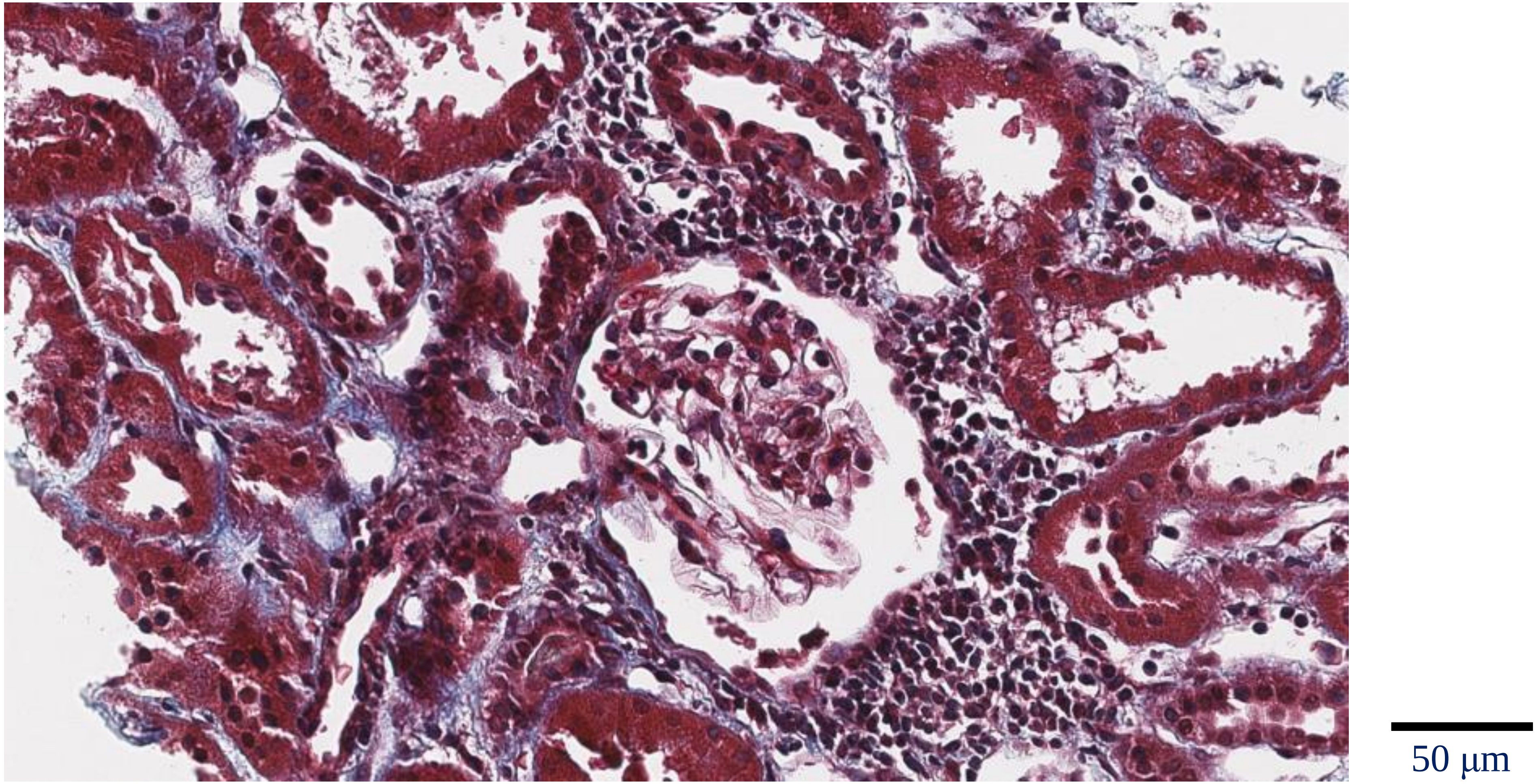

4 – Perfect
3 – Very good
2 – Good enough
1 – Not acceptable

	Stain quality score	Nuclear detail	Cytoplasmic detail	Extracellular Fibrosis
Score				

Stain-transformed image #6

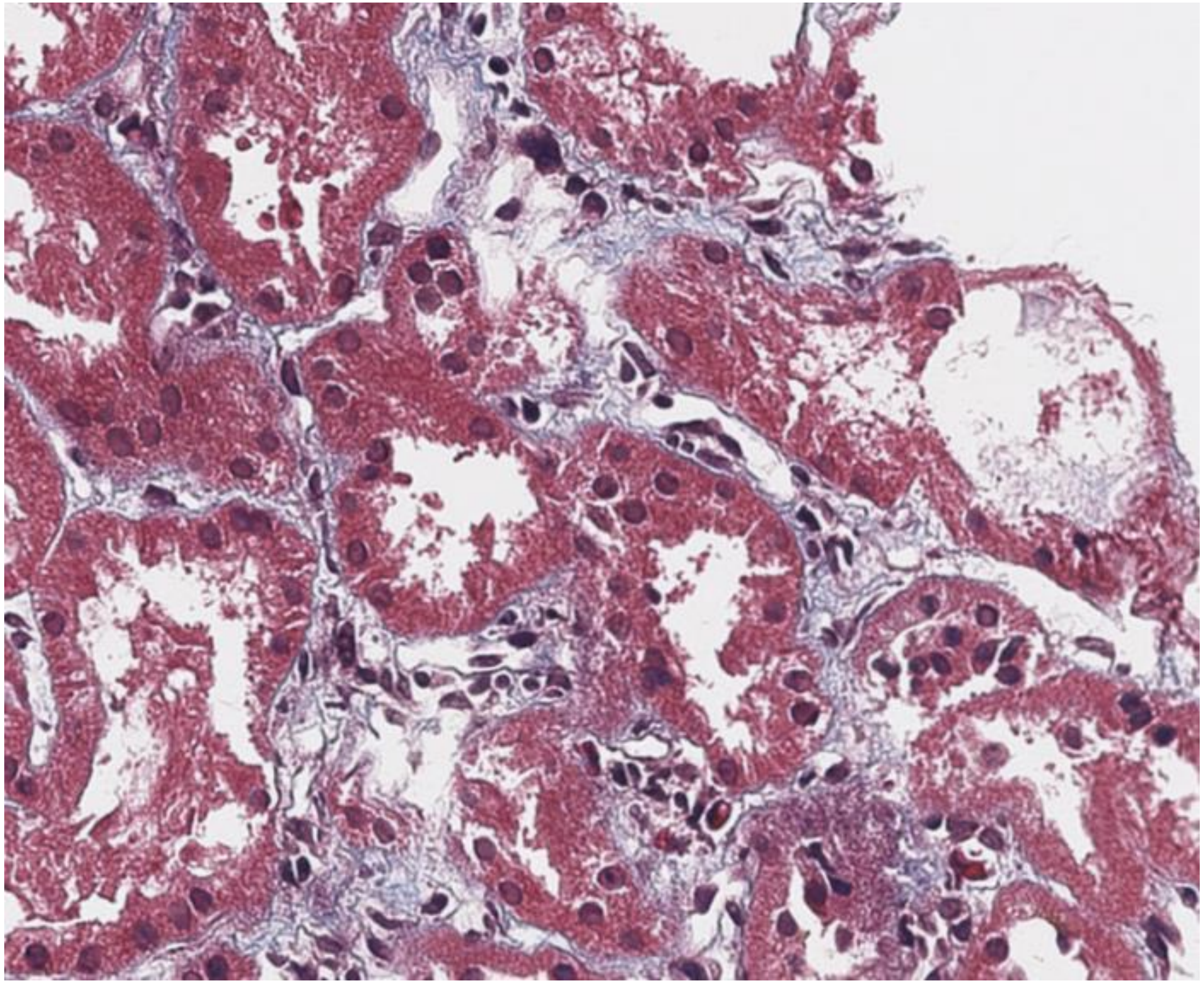

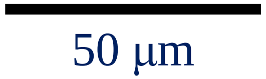

4 – Perfect
3 – Very good
2 – Good enough
1 – Not acceptable

	Stain quality score	Nuclear detail	Cytoplasmic detail	Extracellular Fibrosis
Score				

Histochemically stained image #6

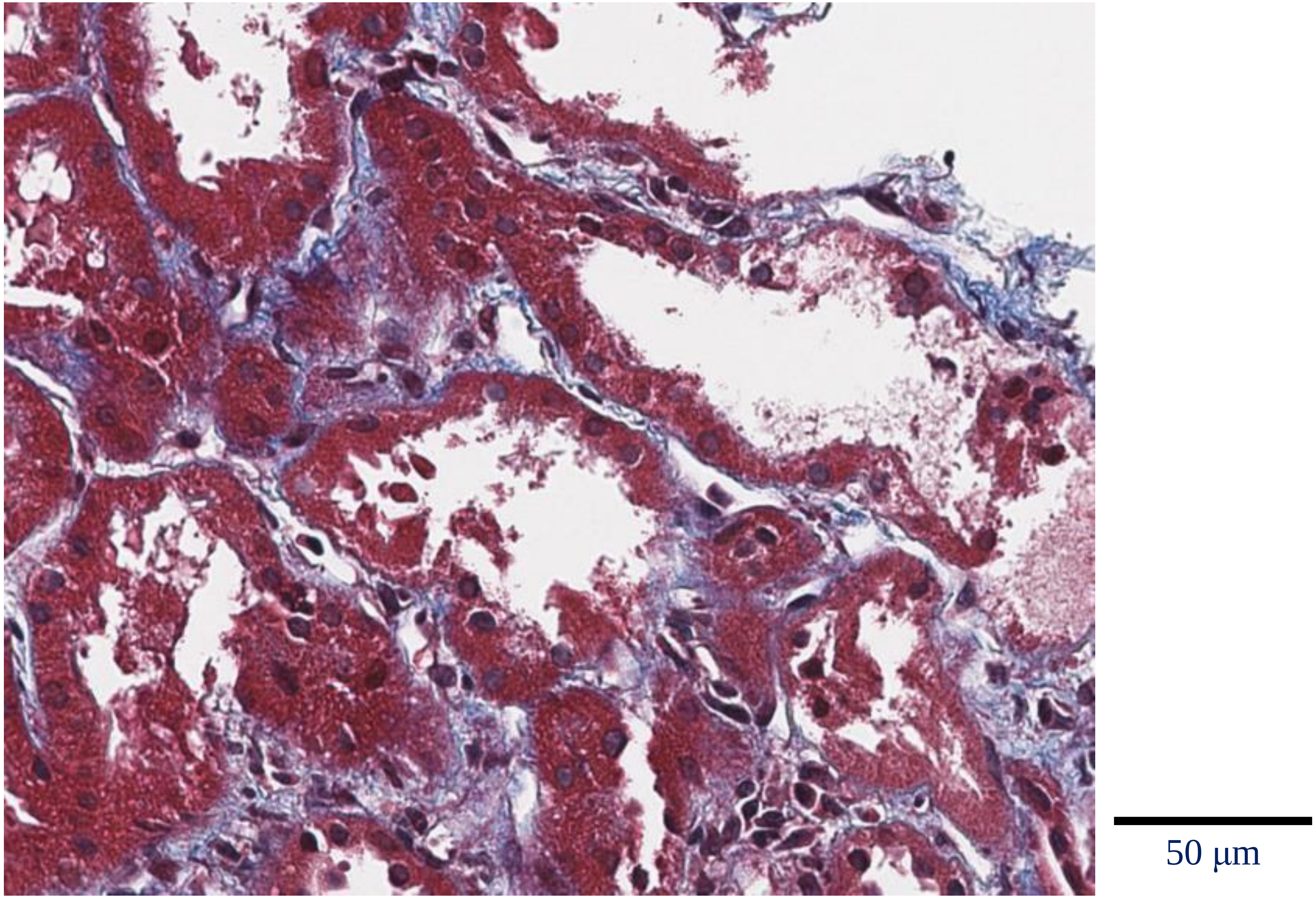

4 – Perfect
3 – Very good
2 – Good enough
1 – Not acceptable

	Stain quality score	Nuclear detail	Cytoplasmic detail	Extracellular Fibrosis
Score				

Stain-transformed image #7

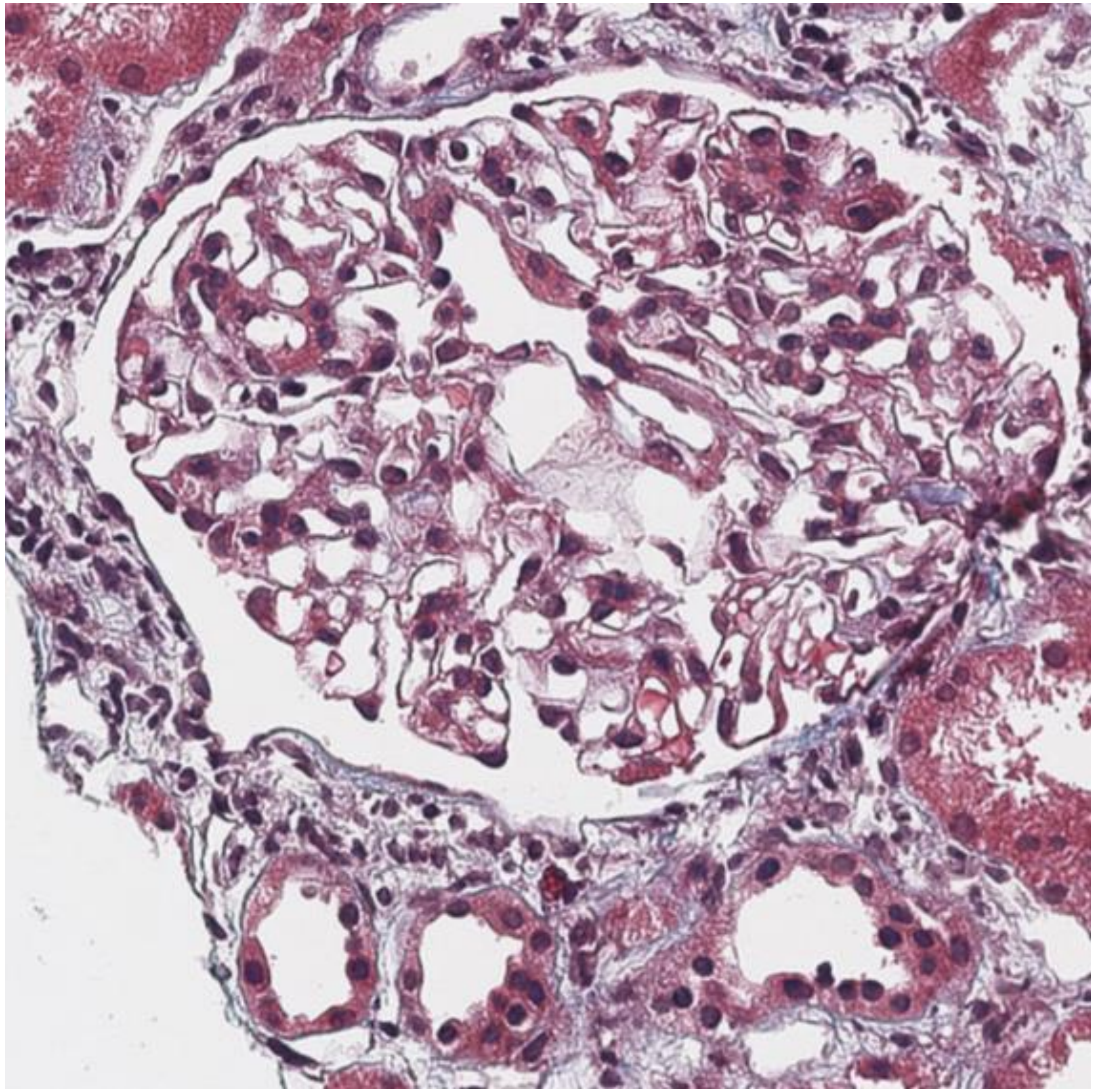

50 µm

4 – Perfect
3 – Very good
2 – Good enough
1 – Not acceptable

	Stain quality score	Nuclear detail	Cytoplasmic detail	Extracellular Fibrosis
Score				

Histochemically stained image #7

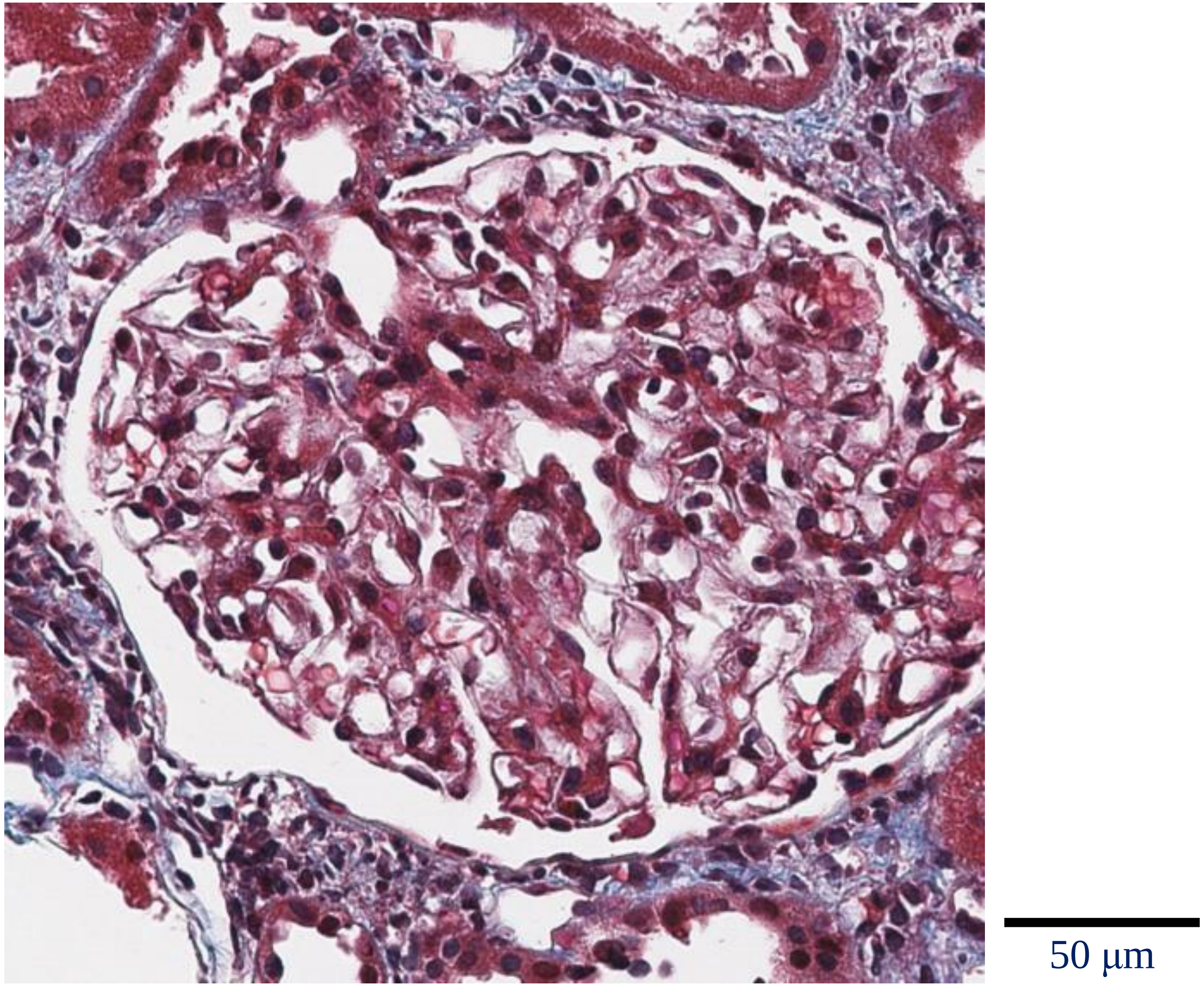

4 – Perfect
3 – Very good
2 – Good enough
1 – Not acceptable

	Stain quality score	Nuclear detail	Cytoplasmic detail	Extracellular Fibrosis
Score				

Stain-transformed image #8

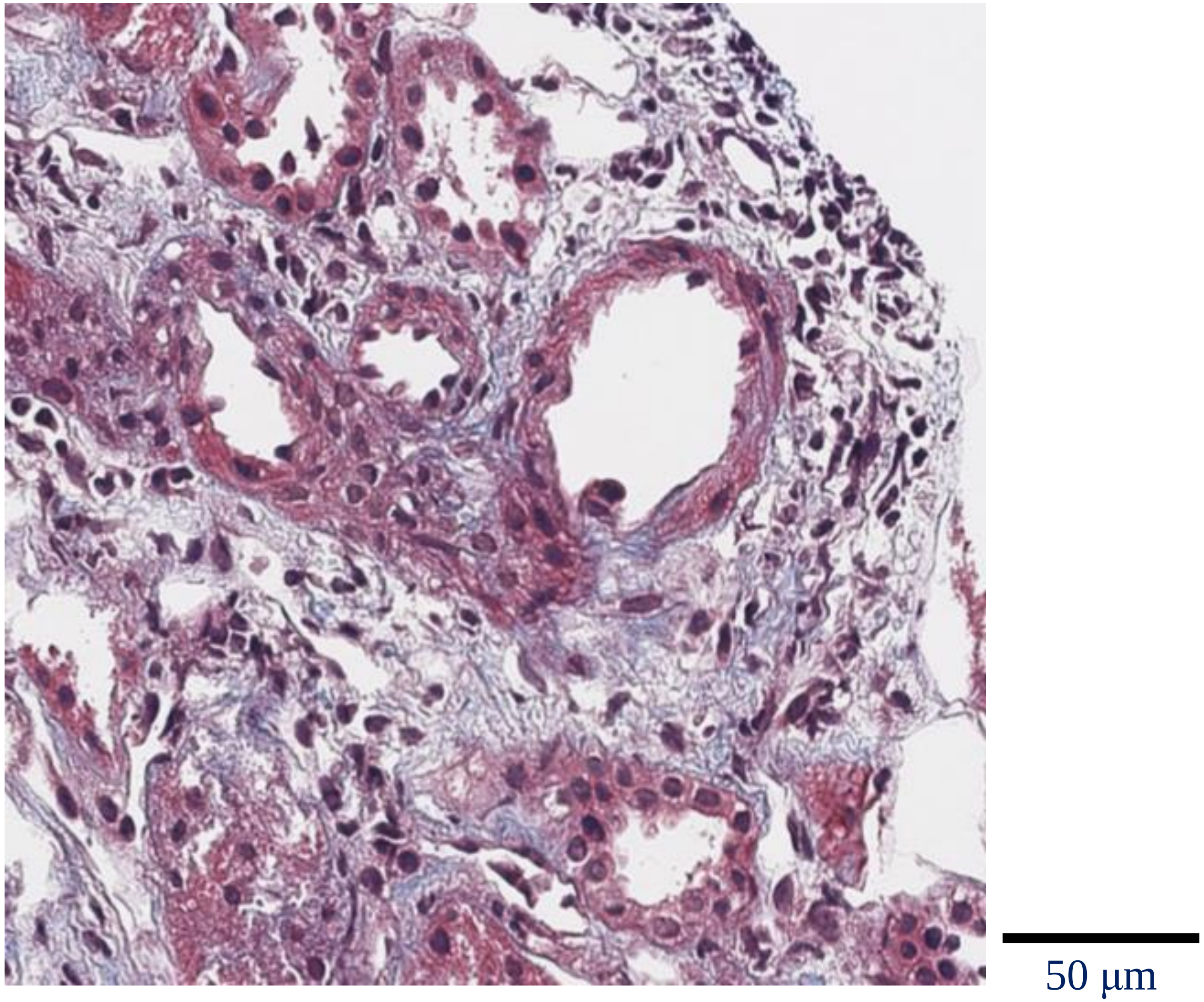

4 – Perfect
3 – Very good
2 – Good enough
1 – Not acceptable

	Stain quality score	Nuclear detail	Cytoplasmic detail	Extracellular Fibrosis
Score				

Histochemically stained image #8

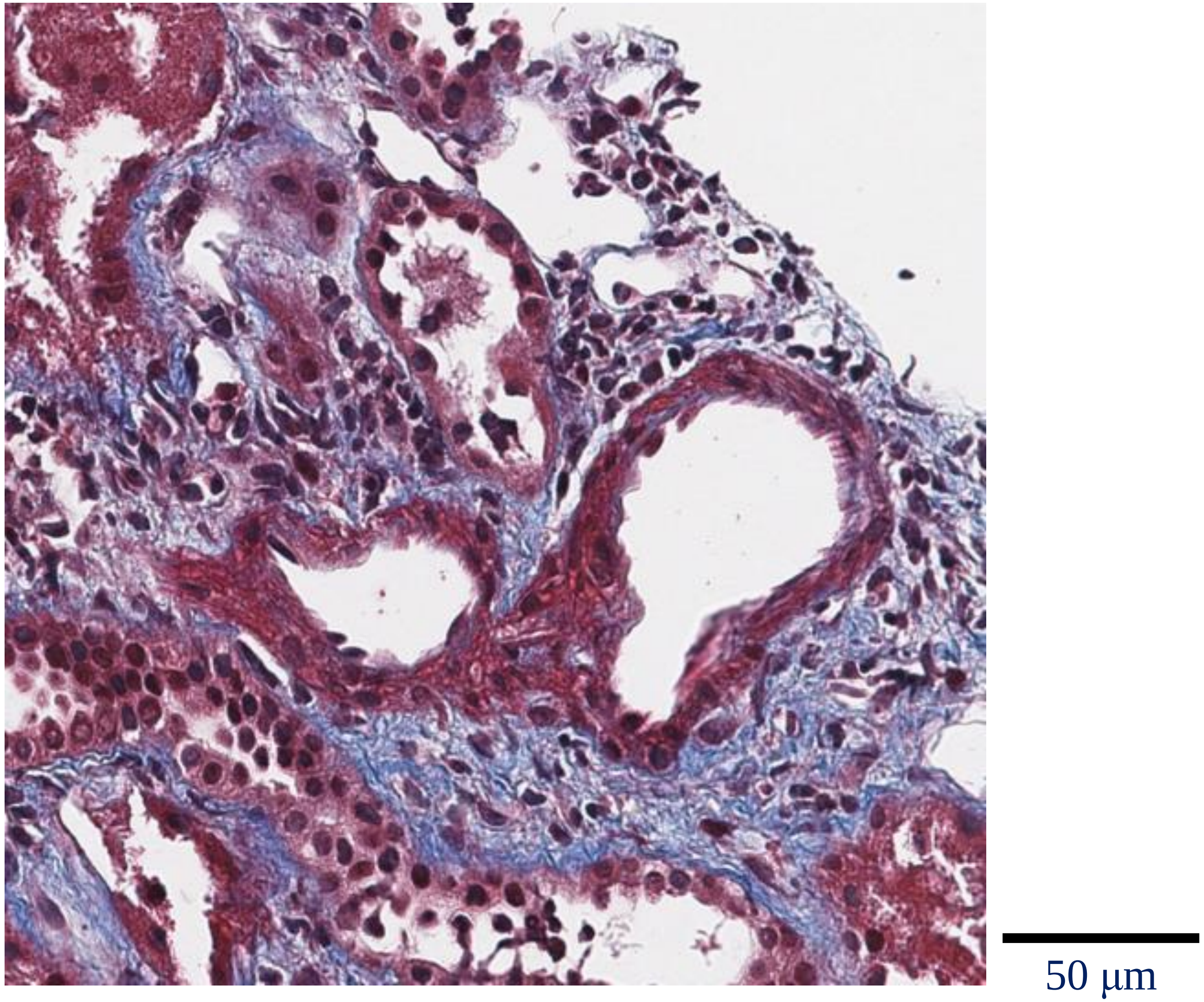

4 – Perfect
3 – Very good
2 – Good enough
1 – Not acceptable

	Stain quality score	Nuclear detail	Cytoplasmic detail	Extracellular Fibrosis
Score				

Stain-transformed image #9

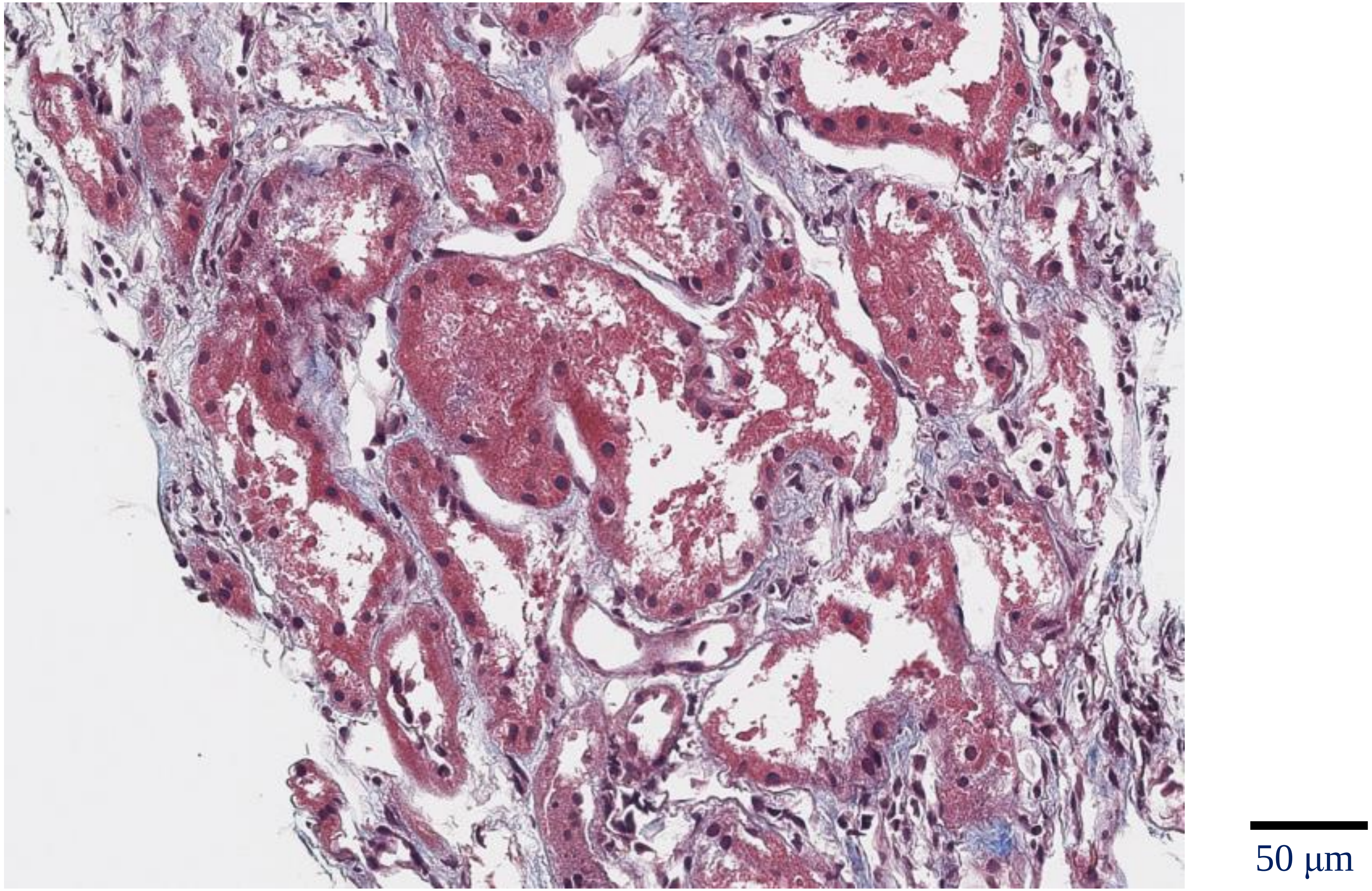

4 – Perfect
3 – Very good
2 – Good enough
1 – Not acceptable

	Stain quality score	Nuclear detail	Cytoplasmic detail	Extracellular Fibrosis
Score				

Histochemically stained image #9

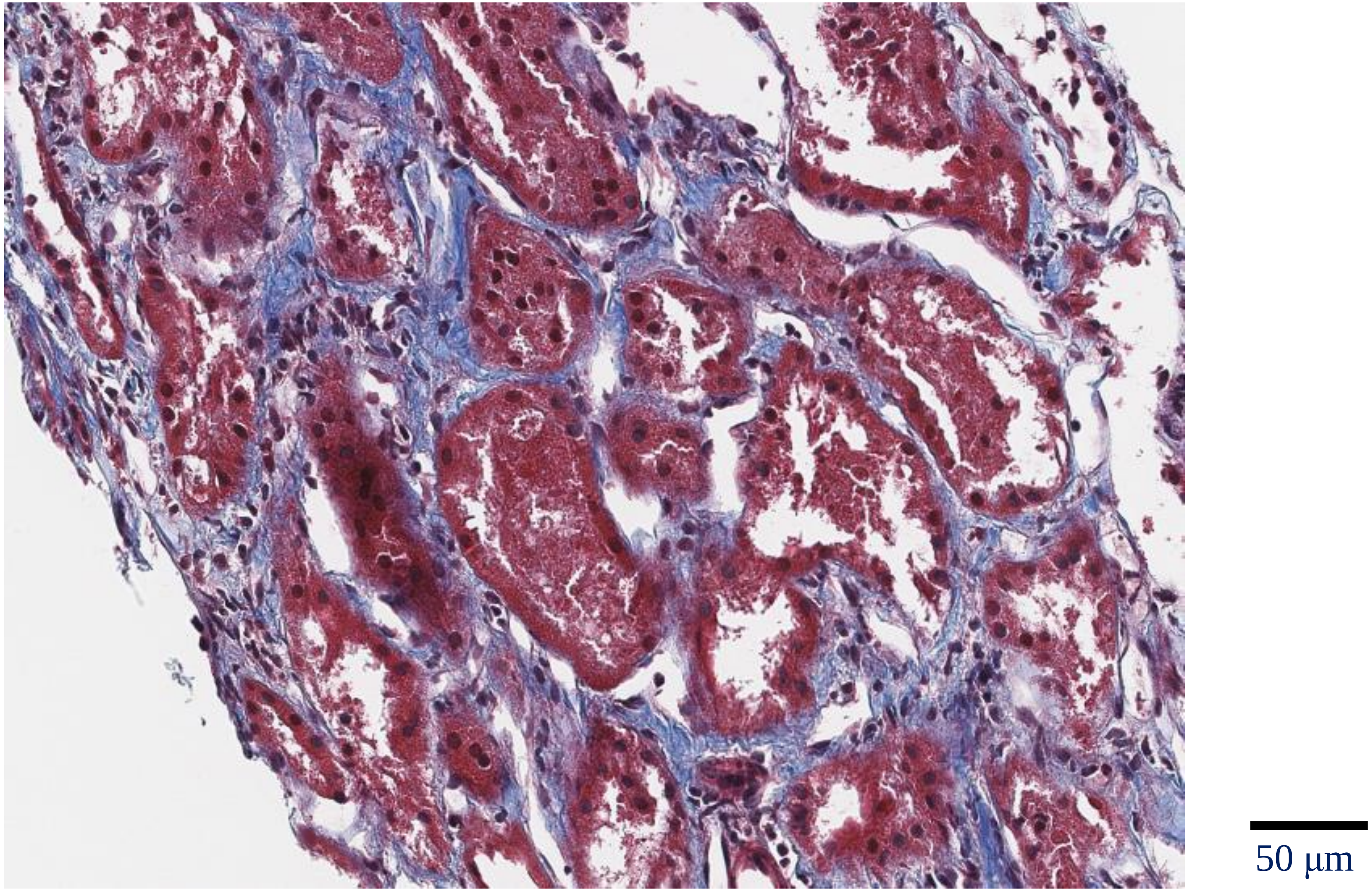

4 – Perfect
3 – Very good
2 – Good enough
1 – Not acceptable

	Stain quality score	Nuclear detail	Cytoplasmic detail	Extracellular Fibrosis
Score				

Stain-transformed image #10

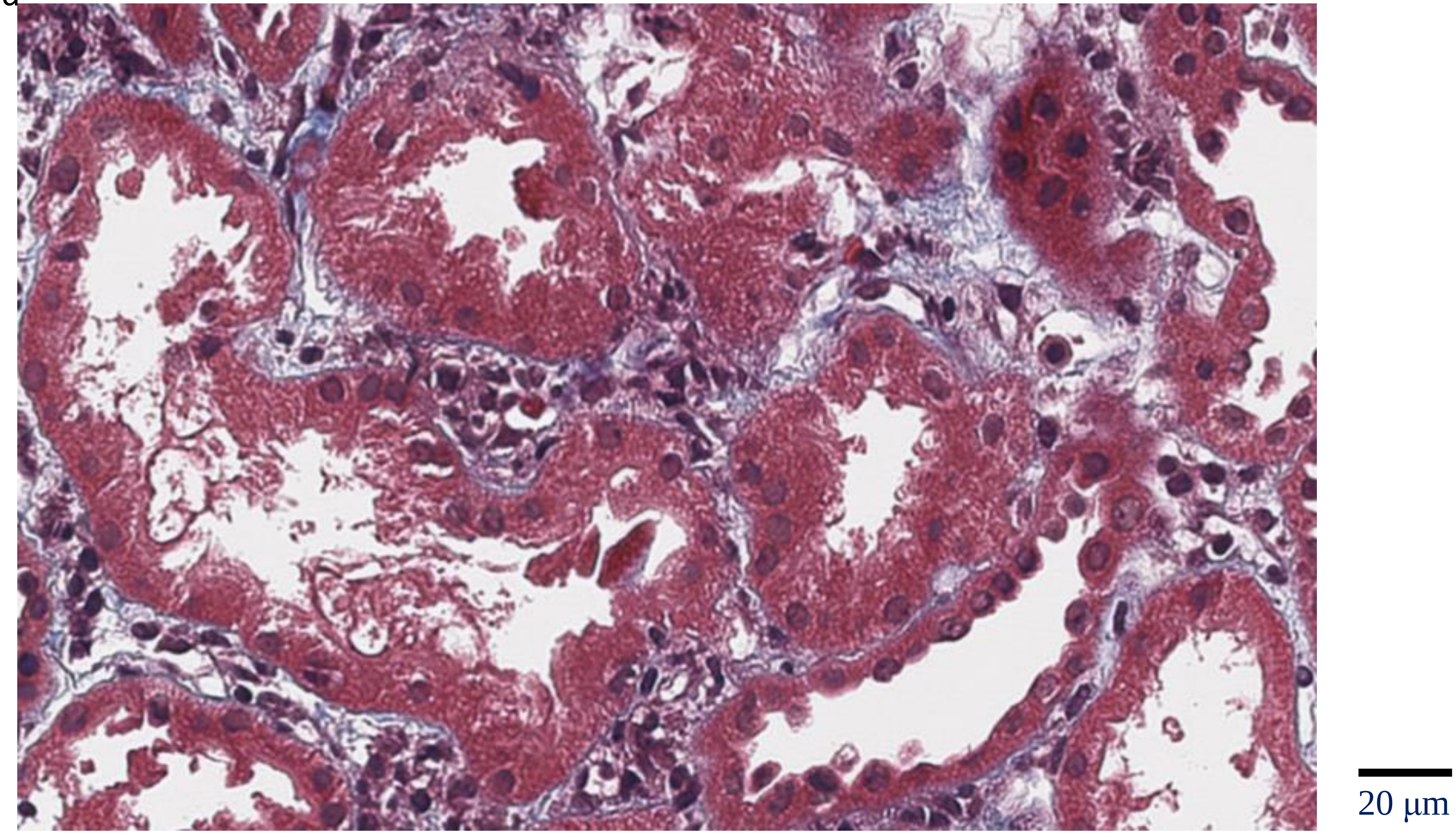

4 – Perfect
3 – Very good
2 – Good enough
1 – Not acceptable

	Stain quality score	Nuclear detail	Cytoplasmic detail	Extracellular Fibrosis
Score				

Histochemically stained image #10

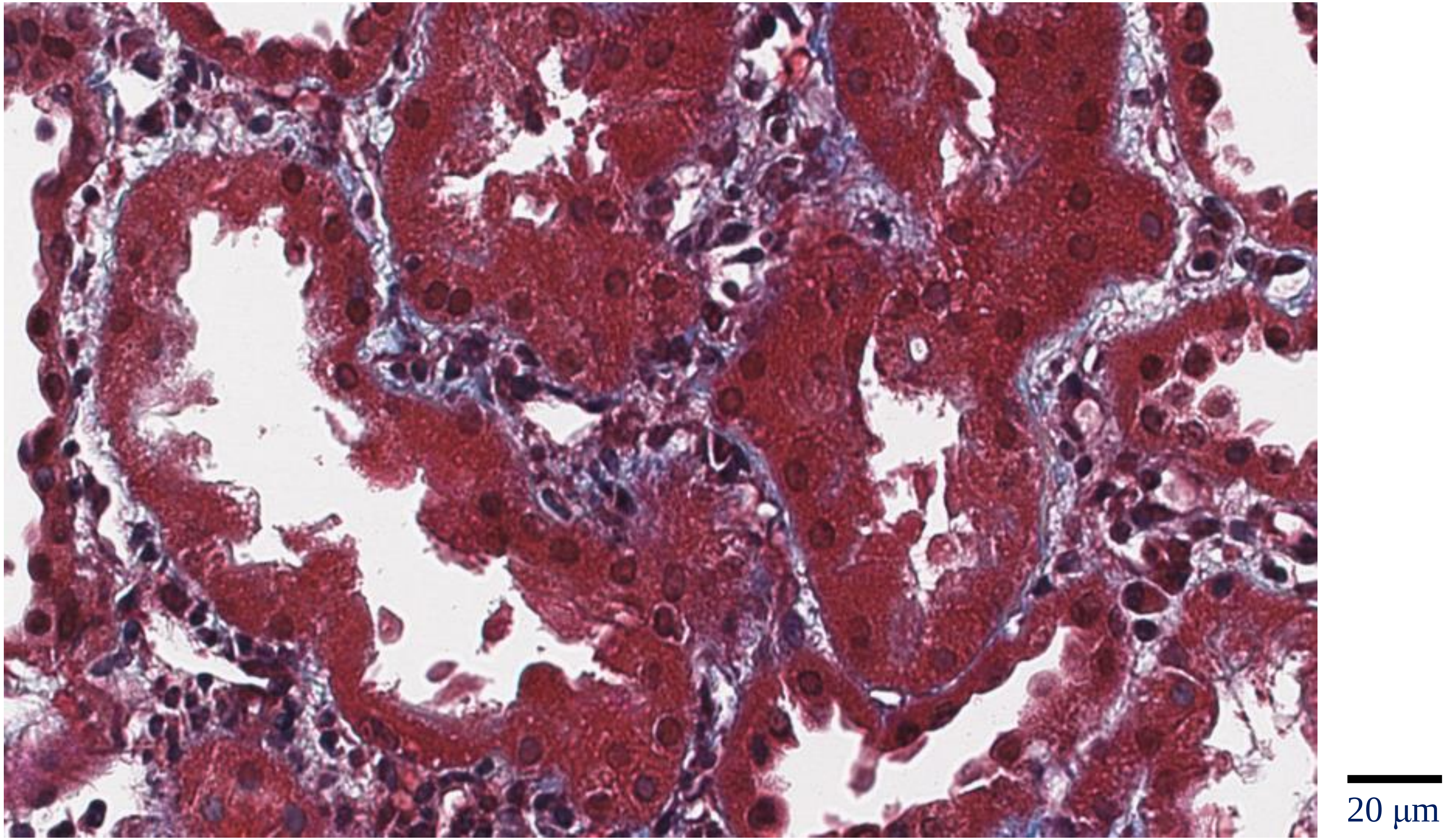

4 – Perfect
3 – Very good
2 – Good enough
1 – Not acceptable

	Stain quality score	Nuclear detail	Cytoplasmic detail	Extracellular Fibrosis
Score				

Stain-transformed image #11

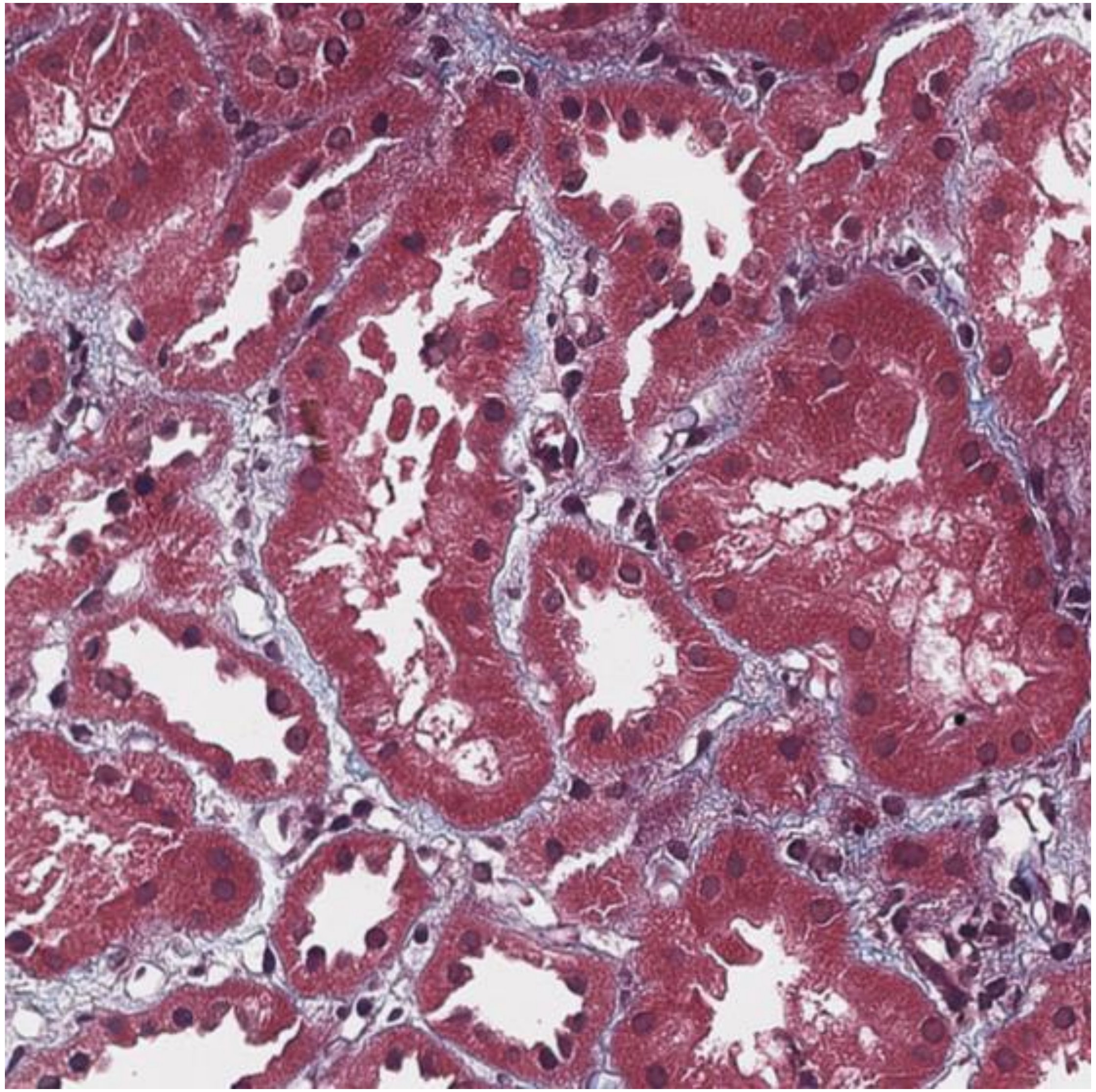

50 μm

4 – Perfect
3 – Very good
2 – Good enough
1 – Not acceptable

	Stain quality score	Nuclear detail	Cytoplasmic detail	Extracellular Fibrosis
Score				

Histochemically stained image #11

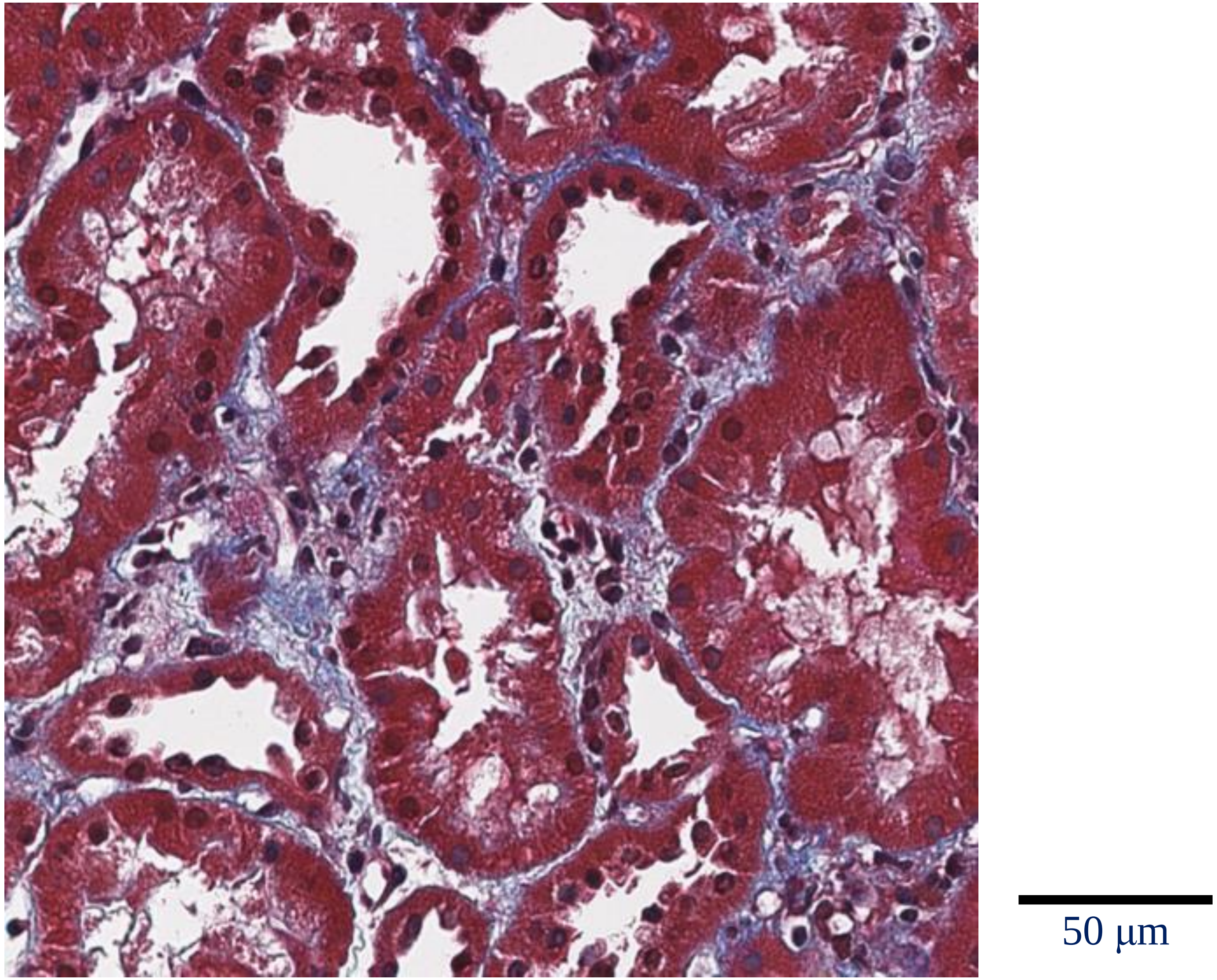

	Stain quality score	Nuclear detail	Cytoplasmic detail	Extracellular Fibrosis
Score				

4 – Perfect
3 – Very good
2 – Good enough
1 – Not acceptable

Stain-transformed image #12

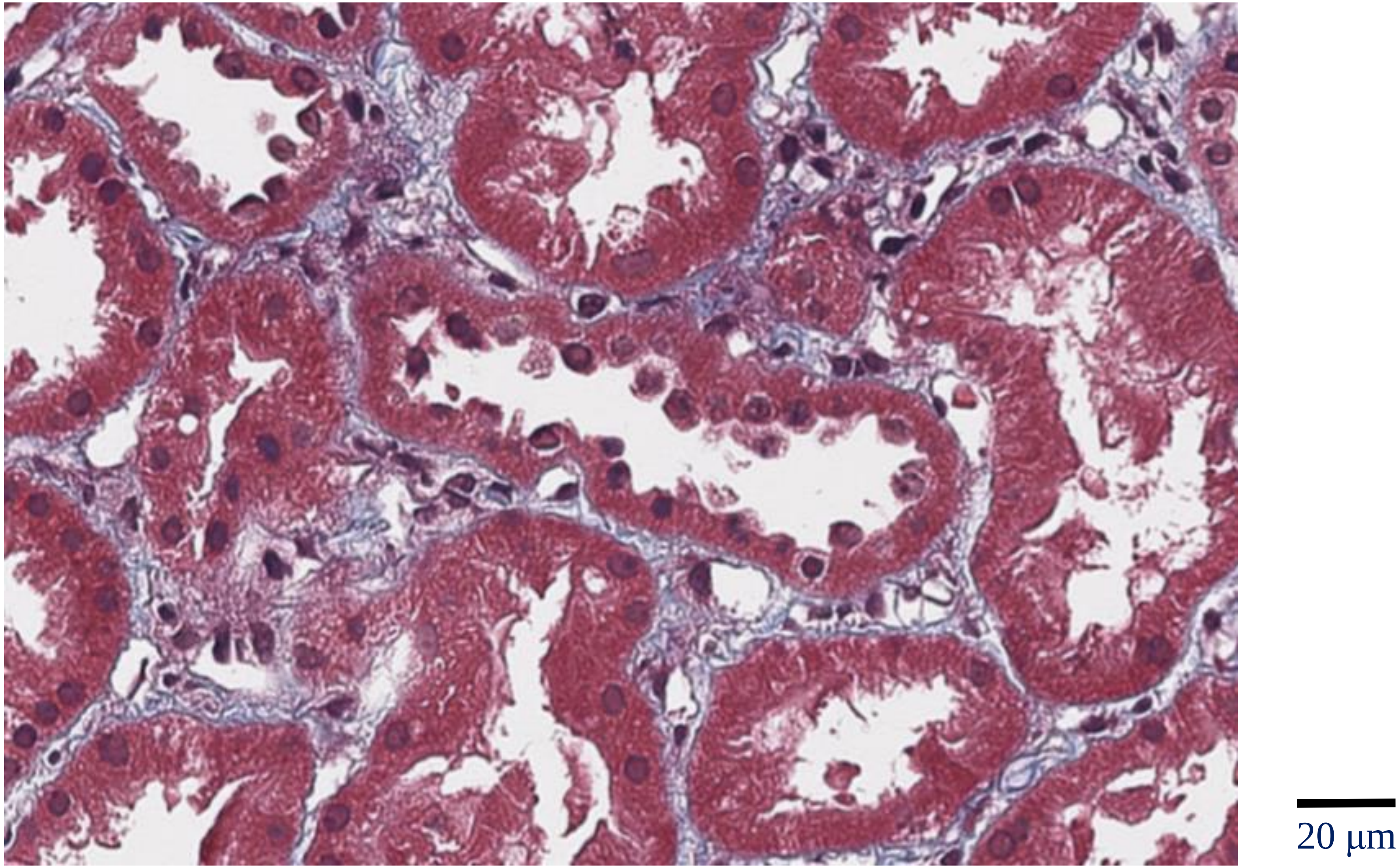

4 – Perfect
3 – Very good
2 – Good enough
1 – Not acceptable

	Stain quality score	Nuclear detail	Cytoplasmic detail	Extracellular Fibrosis
Score				

Histochemically stained image #12

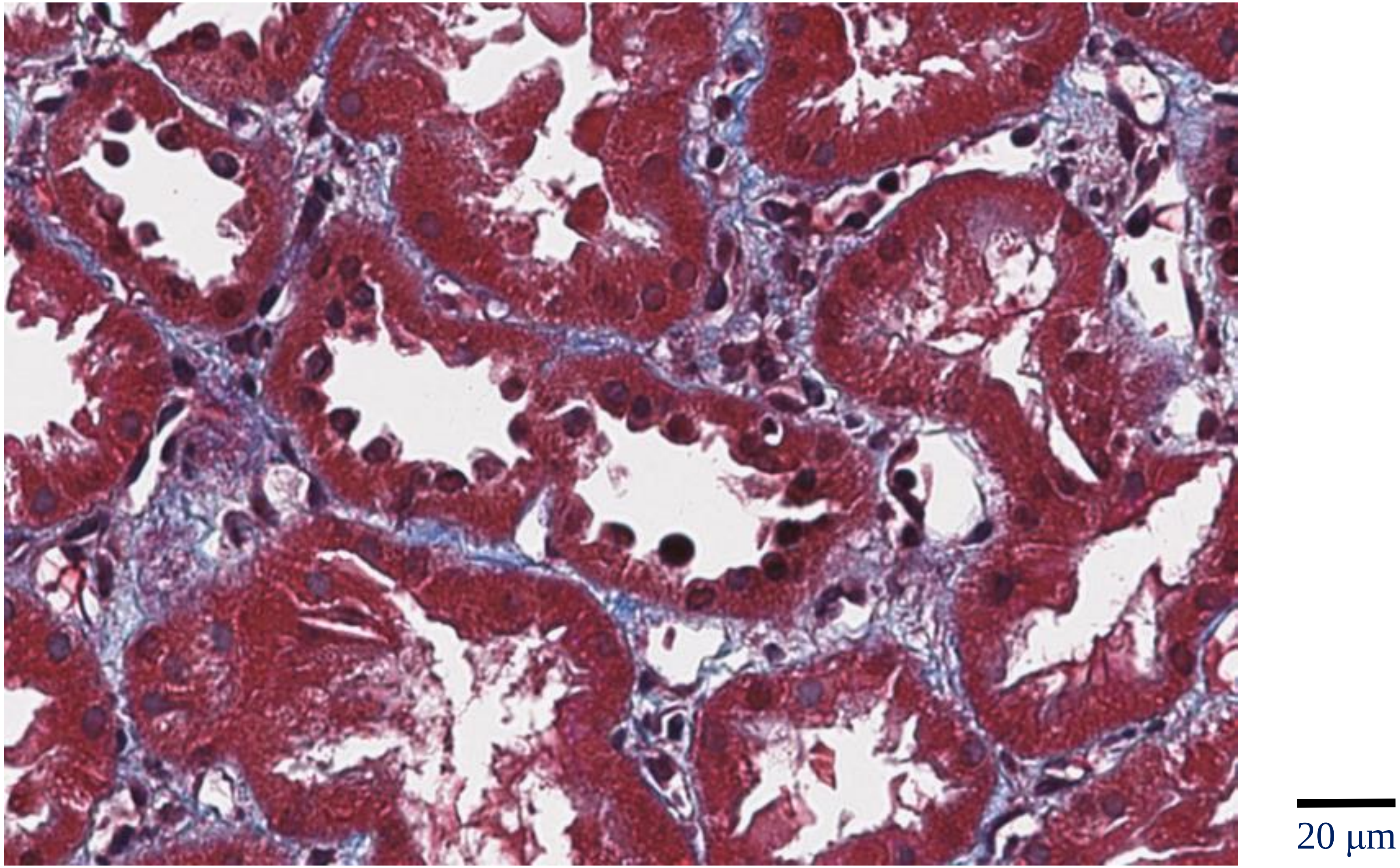

4 – Perfect
3 – Very good
2 – Good enough
1 – Not acceptable

	Stain quality score	Nuclear detail	Cytoplasmic detail	Extracellular Fibrosis
Score				

Stain-transformed image #13

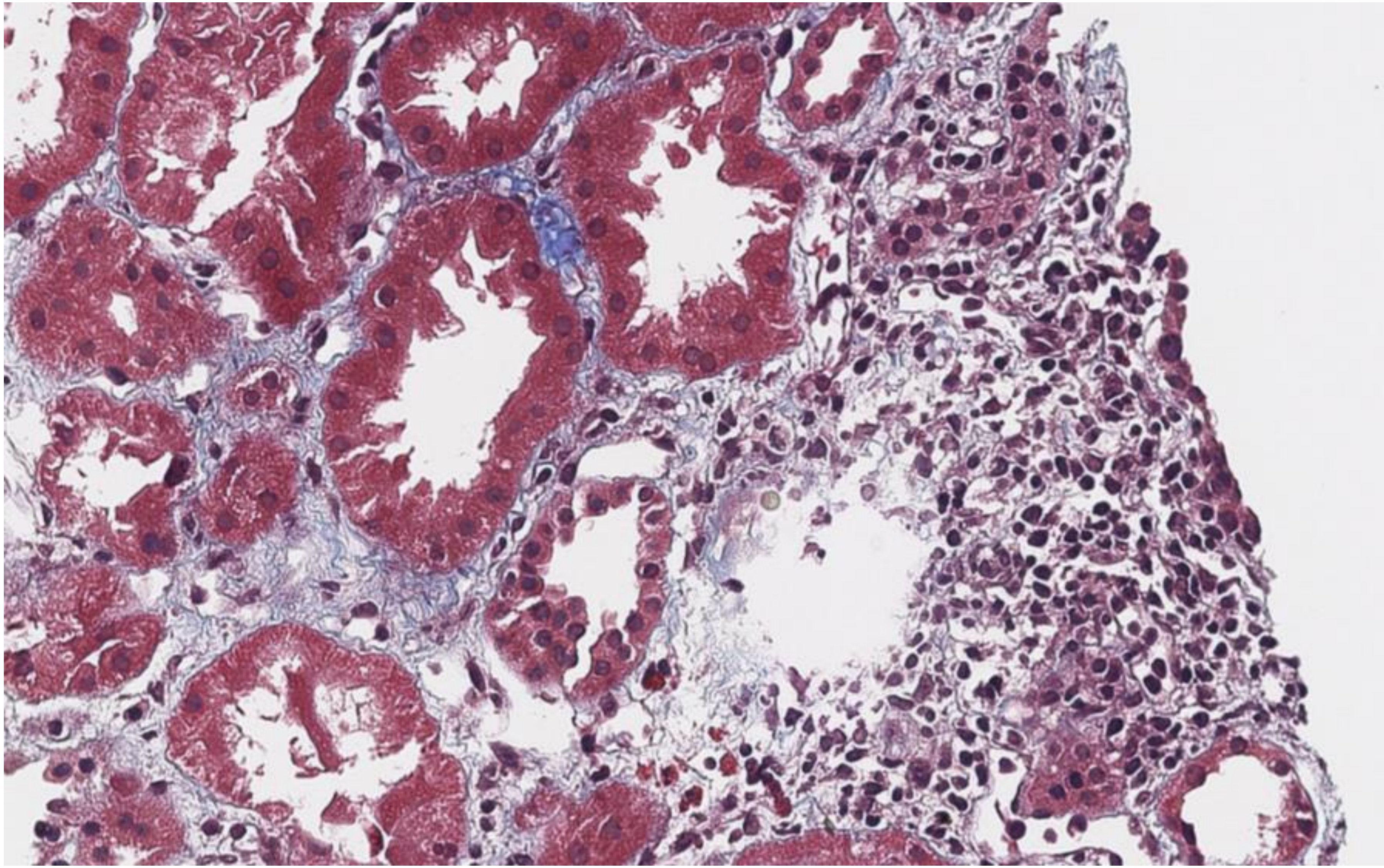

50 µm

4 – Perfect
3 – Very good
2 – Good enough
1 – Not acceptable

	Stain quality score	Nuclear detail	Cytoplasmic detail	Extracellular Fibrosis
Score				

Histochemically stained image #13

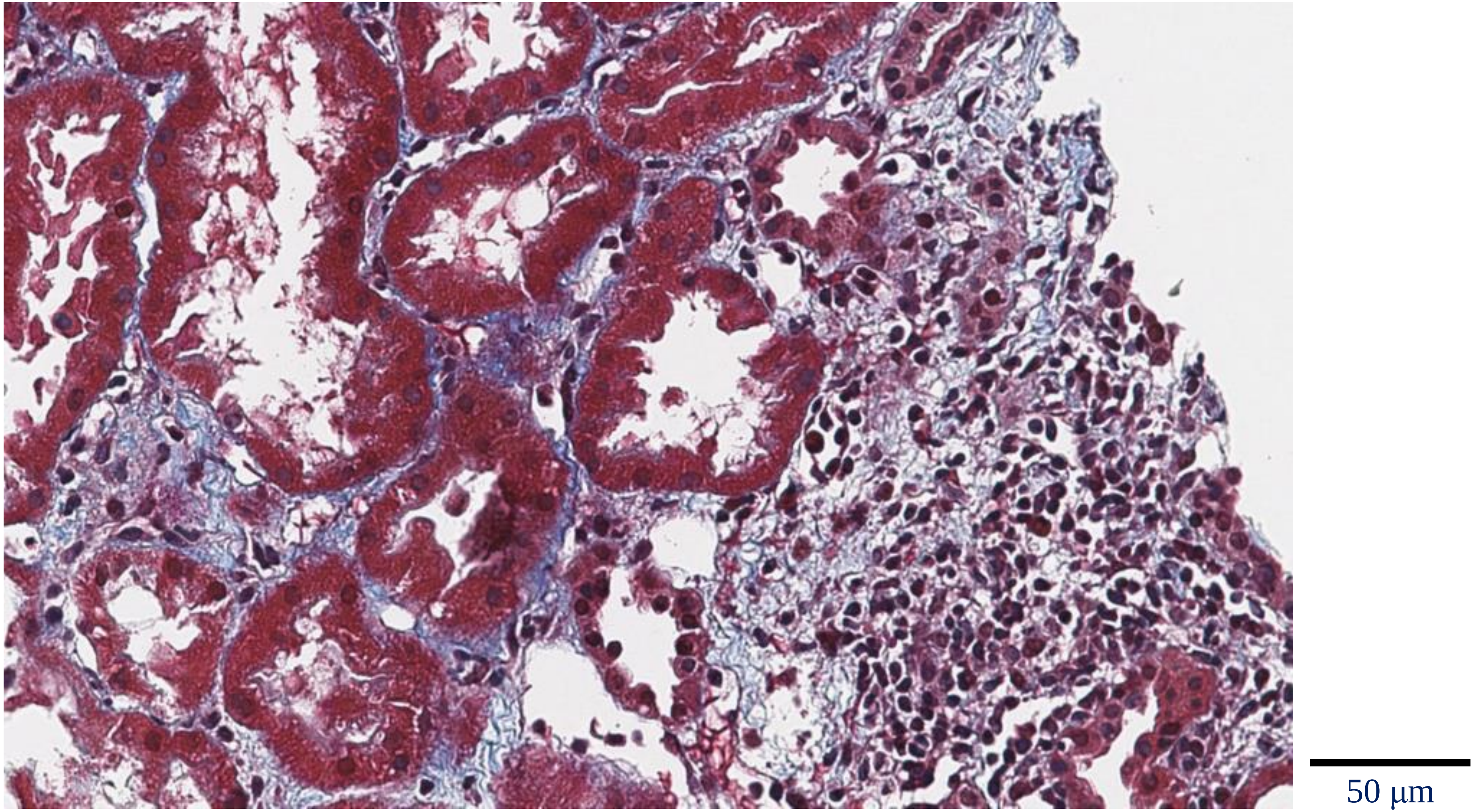

4 – Perfect
3 – Very good
2 – Good enough
1 – Not acceptable

	Stain quality score	Nuclear detail	Cytoplasmic detail	Extracellular Fibrosis
Score				

Stain-transformed image #14

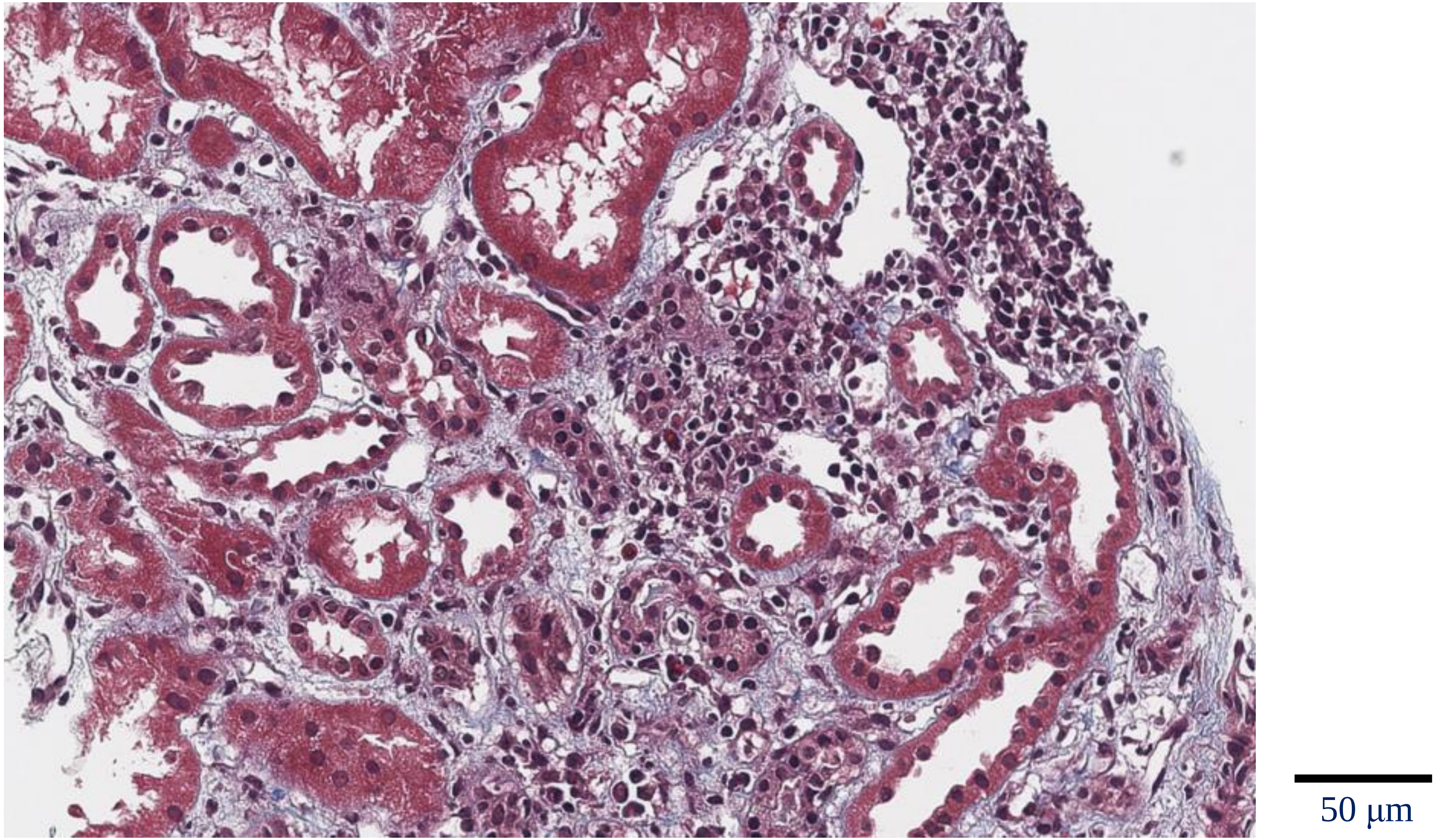

4 – Perfect
3 – Very good
2 – Good enough
1 – Not acceptable

	Stain quality score	Nuclear detail	Cytoplasmic detail	Extracellular Fibrosis
Score				

Histochemically stained image #14

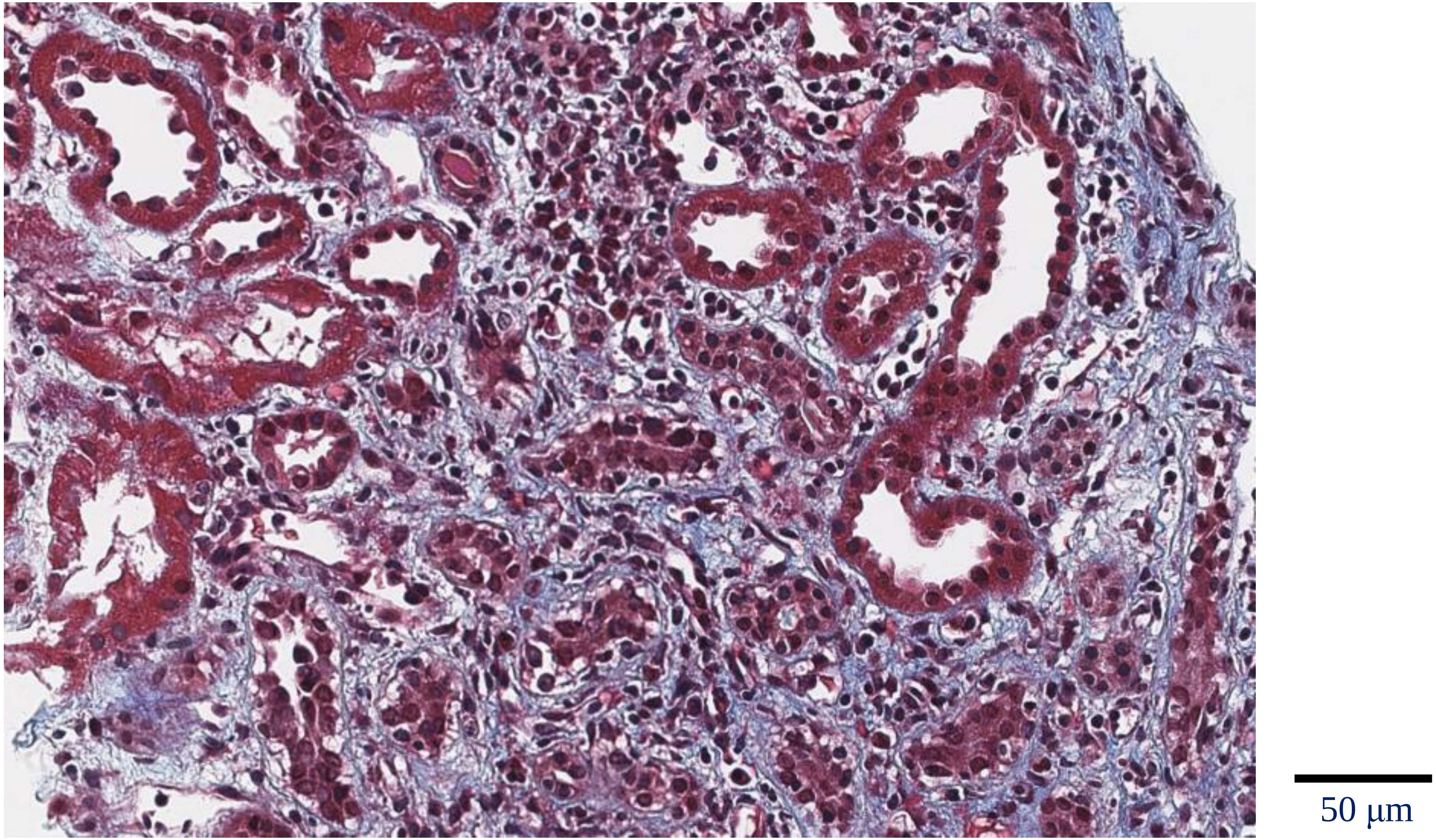

4 – Perfect
3 – Very good
2 – Good enough
1 – Not acceptable

	Stain quality score	Nuclear detail	Cytoplasmic detail	Extracellular Fibrosis
Score				

Stain-transformed image #15

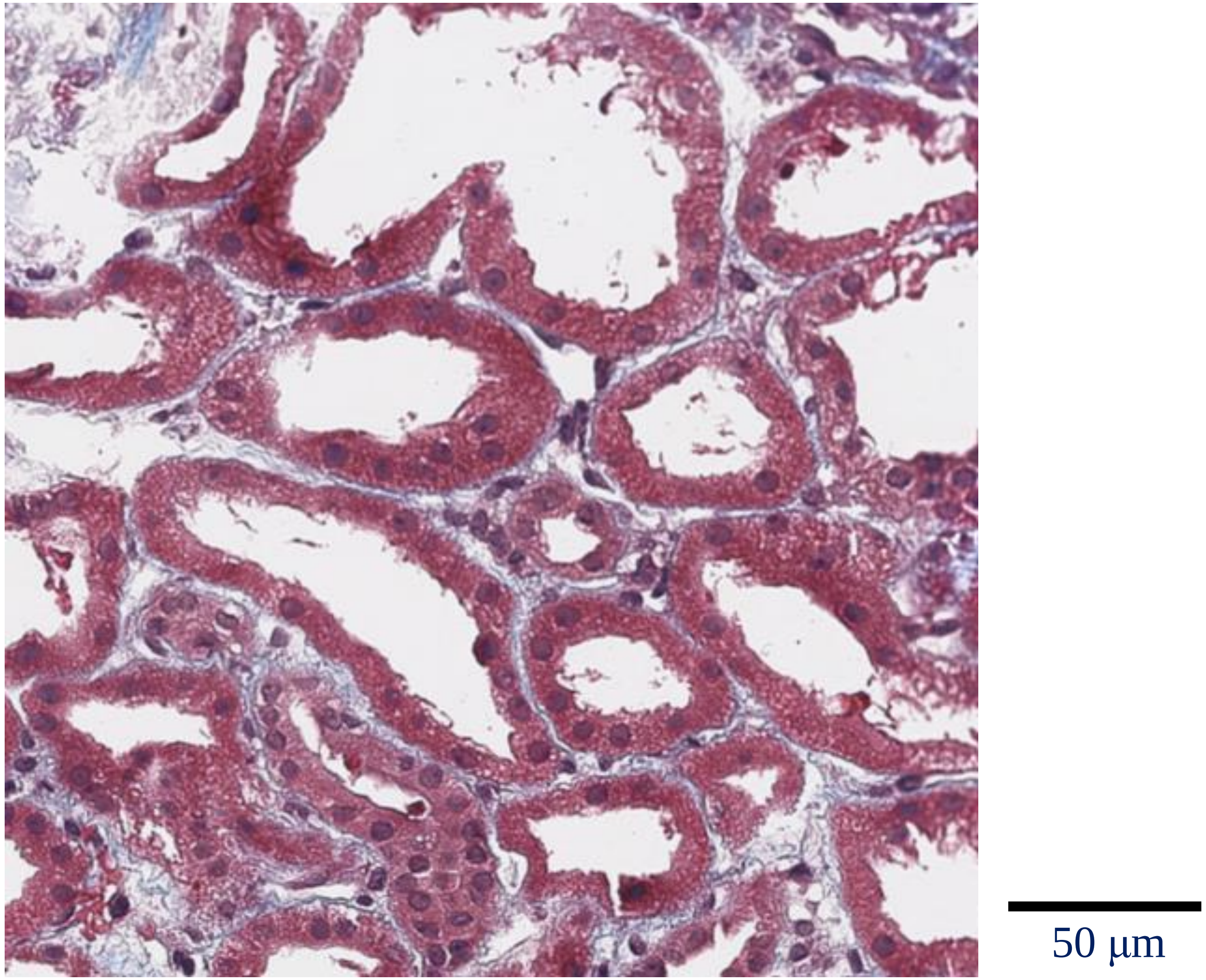

4 – Perfect
3 – Very good
2 – Good enough
1 – Not acceptable

	Stain quality score	Nuclear detail	Cytoplasmic detail	Extracellular Fibrosis
Score				

Histochemically stained image #15

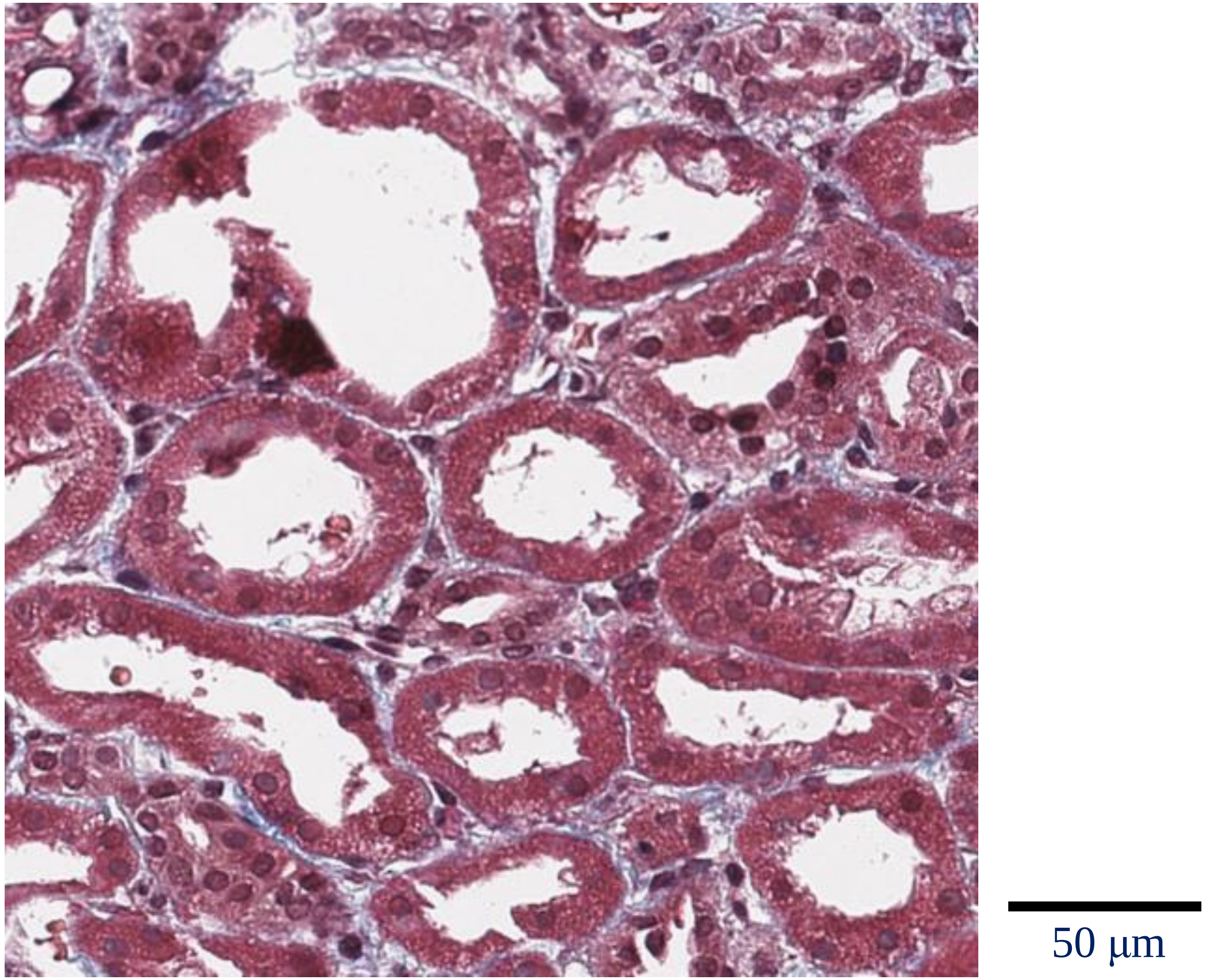

4 – Perfect
3 – Very good
2 – Good enough
1 – Not acceptable

	Stain quality score	Nuclear detail	Cytoplasmic detail	Extracellular Fibrosis
Score				

Stain-transformed image #16

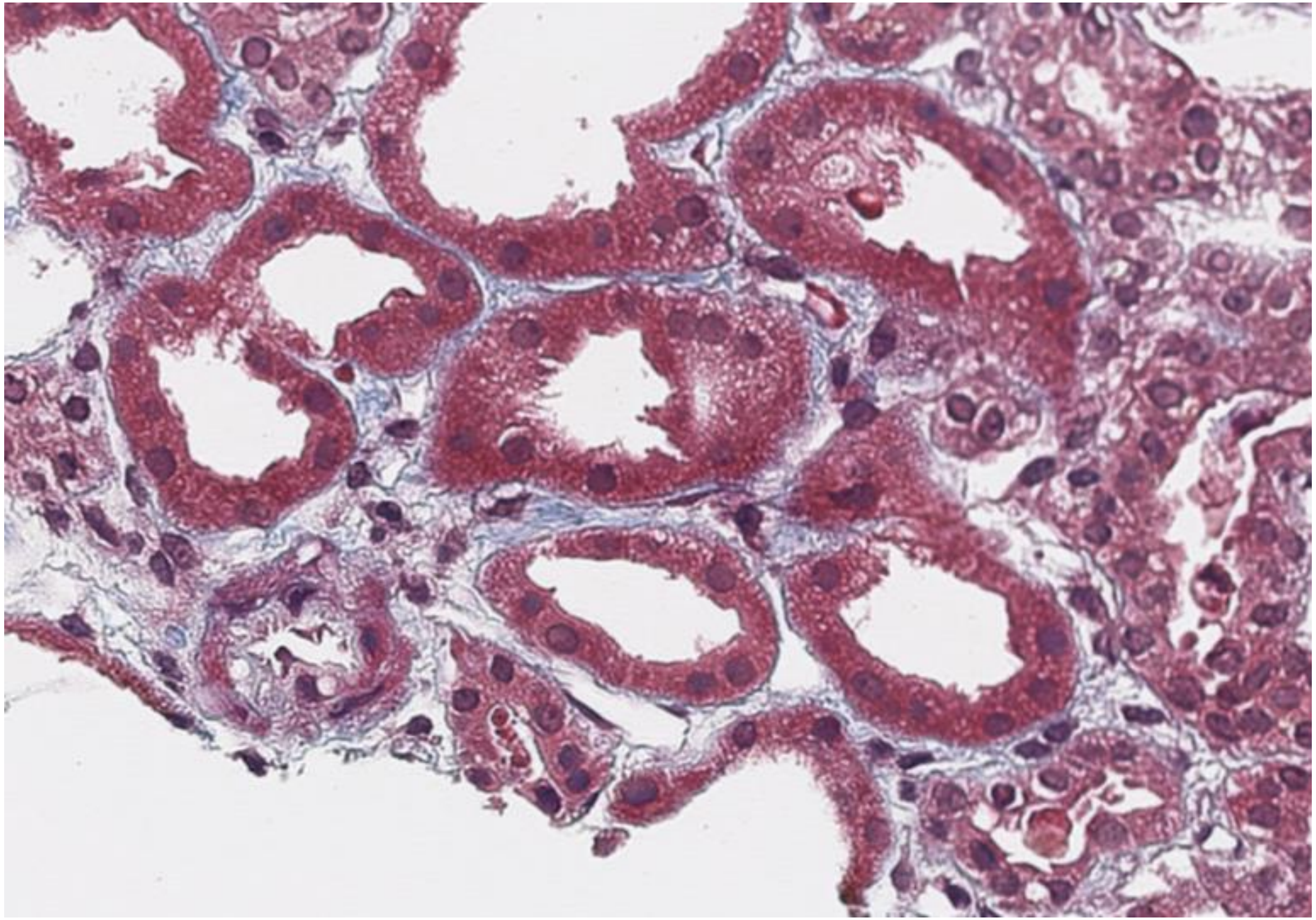

20 µm

	Stain quality score	Nuclear detail	Cytoplasmic detail	Extracellular Fibrosis
Score				

4 – Perfect
3 – Very good
2 – Good enough
1 – Not acceptable

Histochemically stained image #16

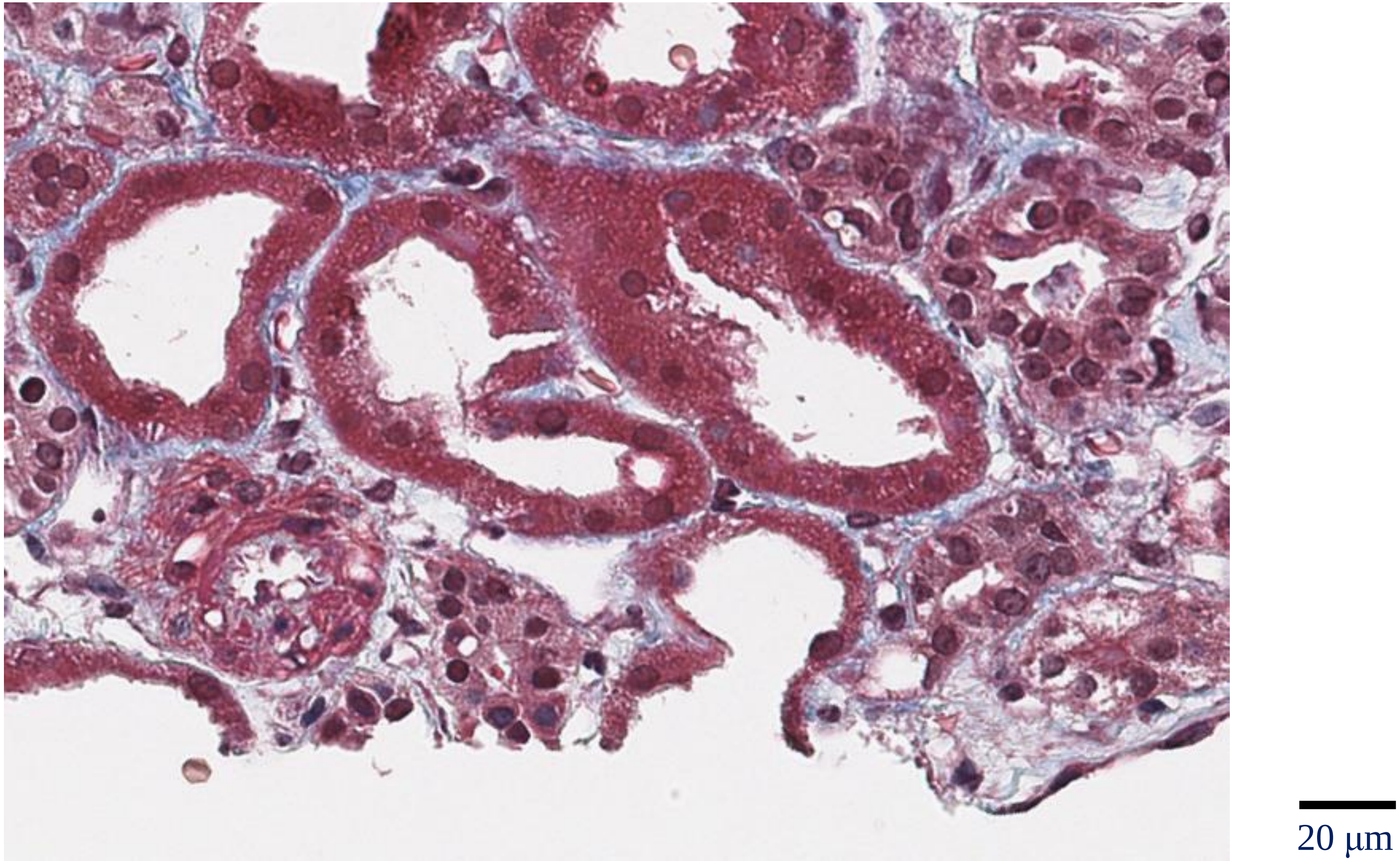

4 – Perfect
3 – Very good
2 – Good enough
1 – Not acceptable

	Stain quality score	Nuclear detail	Cytoplasmic detail	Extracellular Fibrosis
Score				

PAS

Stain-transformed image #1

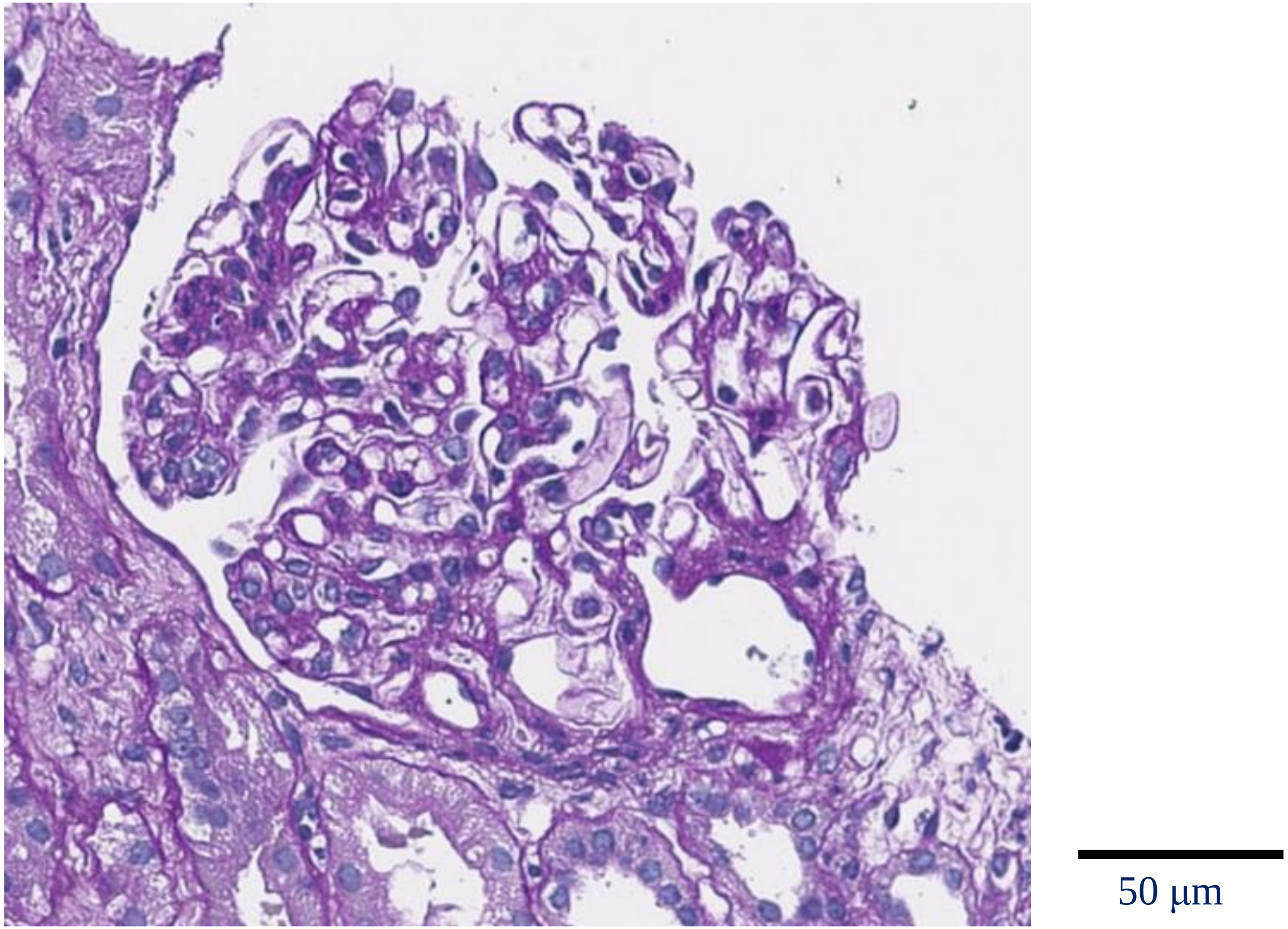

4 – Perfect
3 – Very good
2 – Good enough
1 – Not acceptable

	Stain quality score	Nuclear detail	Cytoplasmic detail	Basement membrane detail
Score				

Histochemically
stained image #1

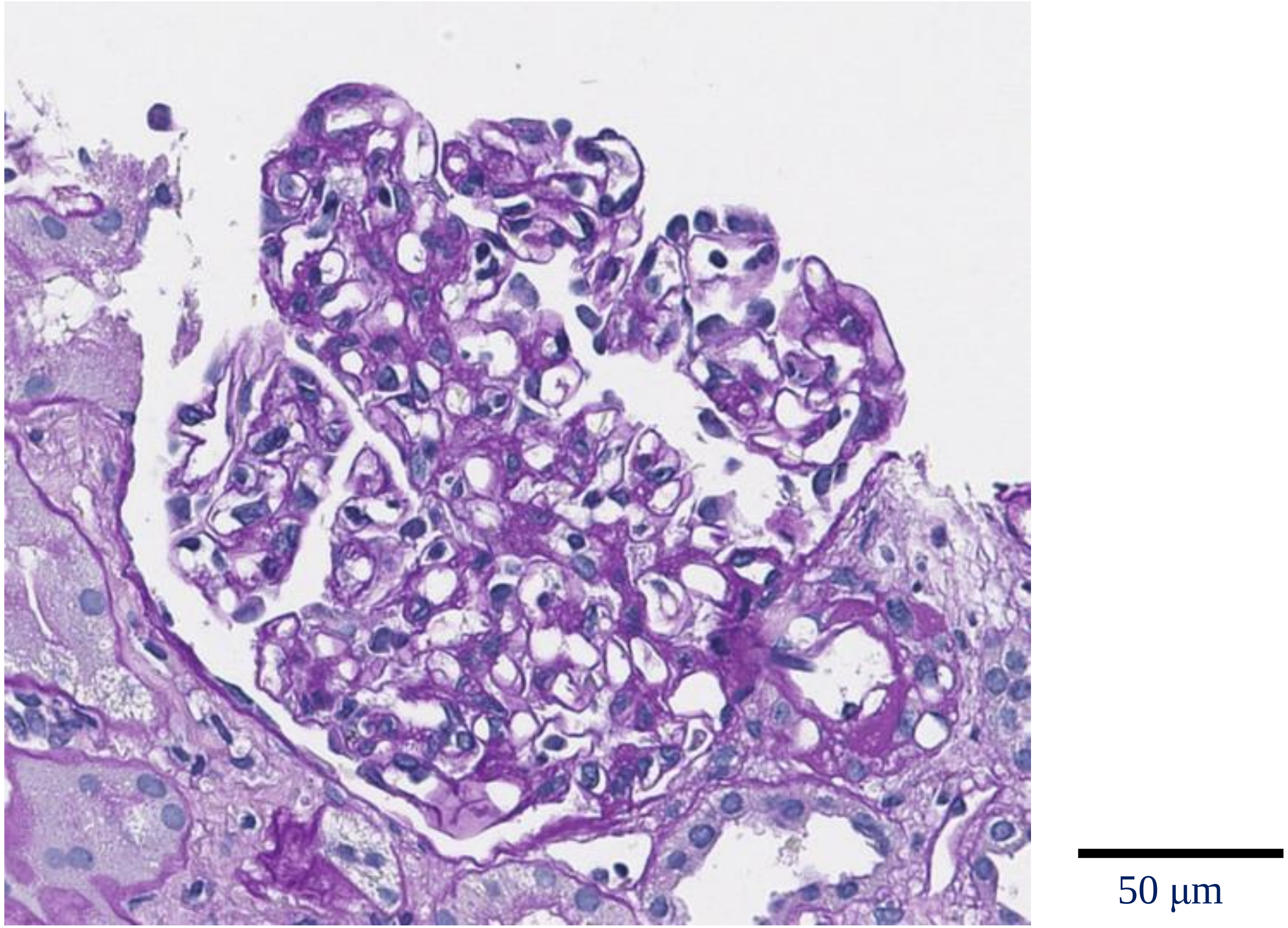

4 – Perfect
3 – Very good
2 – Good enough
1 – Not acceptable

	Stain quality score	Nuclear detail	Cytoplasmic detail	Basement membrane detail
Score				

Stain-transformed image #2

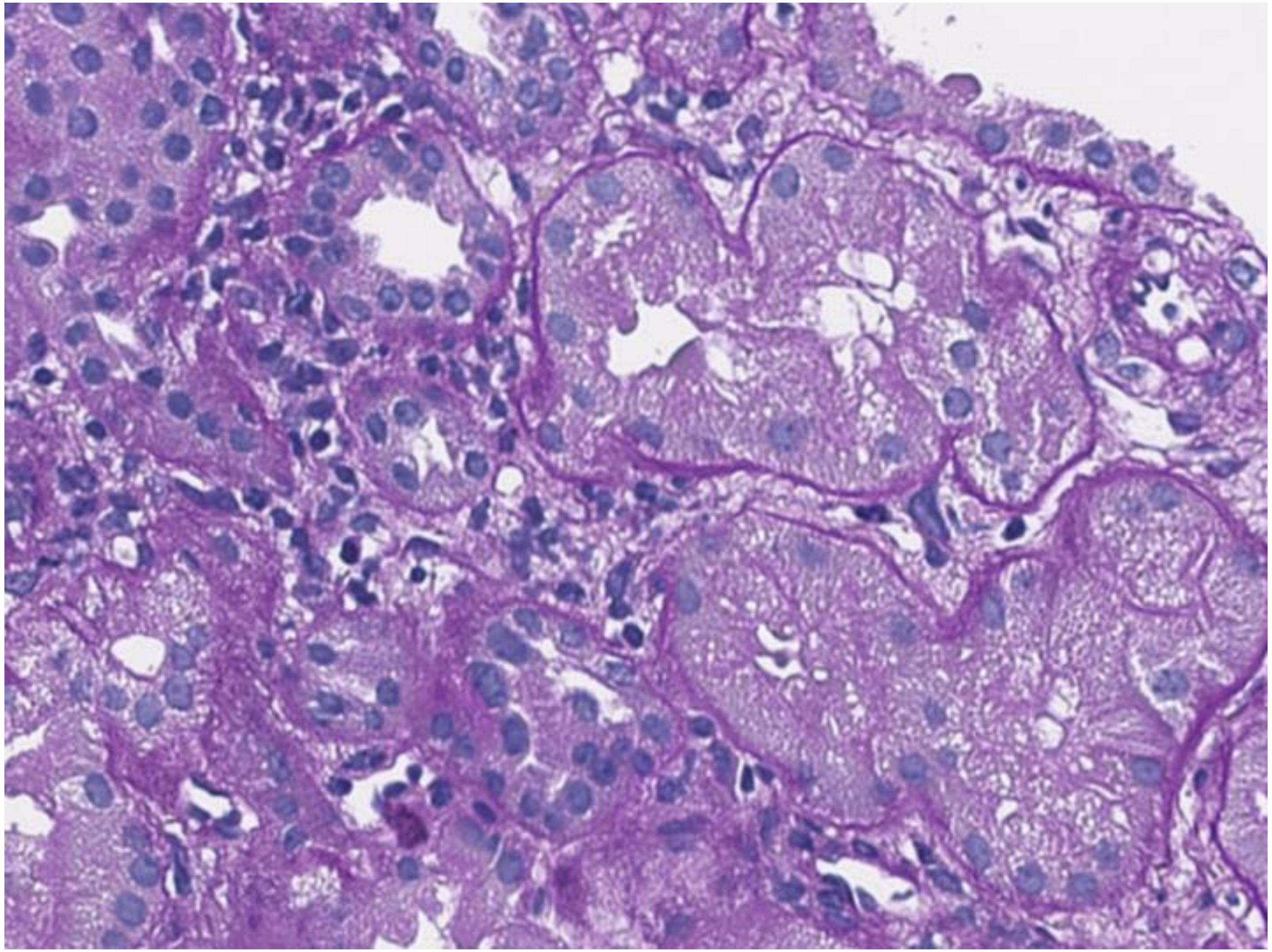

50 µm

4 – Perfect
3 – Very good
2 – Good enough
1 – Not acceptable

	Stain quality score	Nuclear detail	Cytoplasmic detail	Basement membrane detail
Score				

Histochemically stained image #2

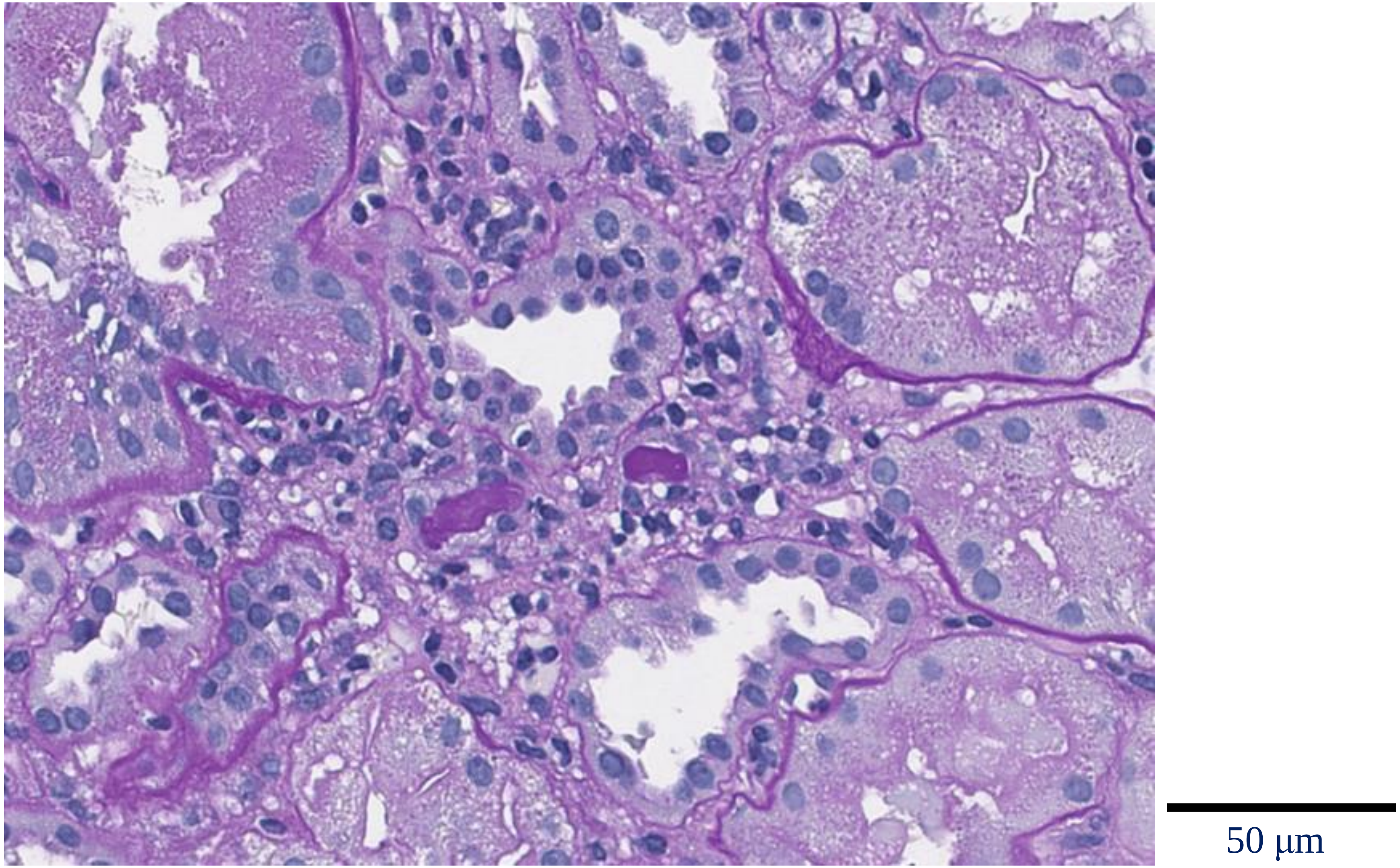

4 – Perfect
3 – Very good
2 – Good enough
1 – Not acceptable

	Stain quality score	Nuclear detail	Cytoplasmic detail	Basement membrane detail
Score				

Stain-transformed
image #3

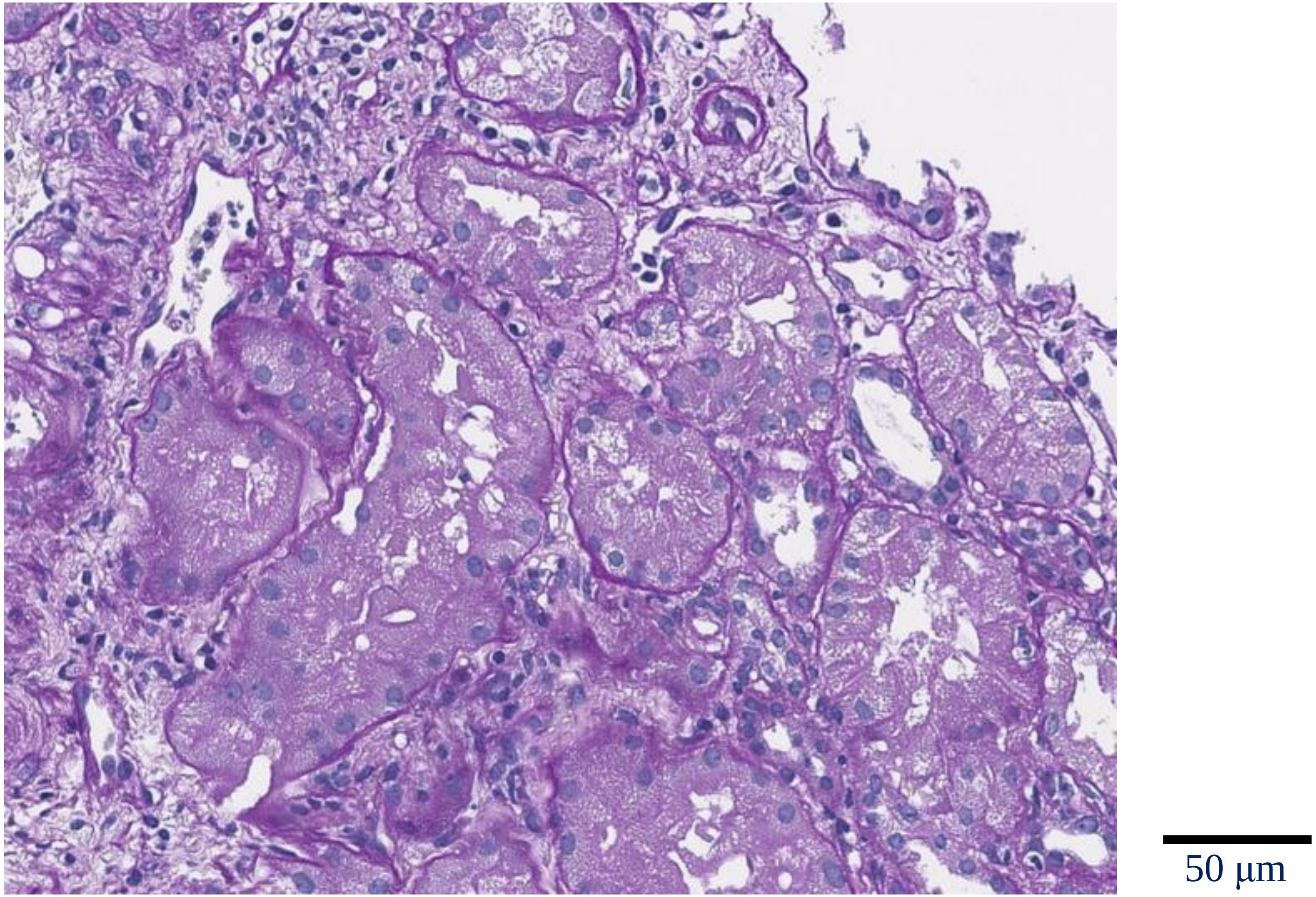

4 – Perfect
3 – Very good
2 – Good enough
1 – Not acceptable

	Stain quality score	Nuclear detail	Cytoplasmic detail	Basement membrane detail
Score				

Histochemically stained image #3

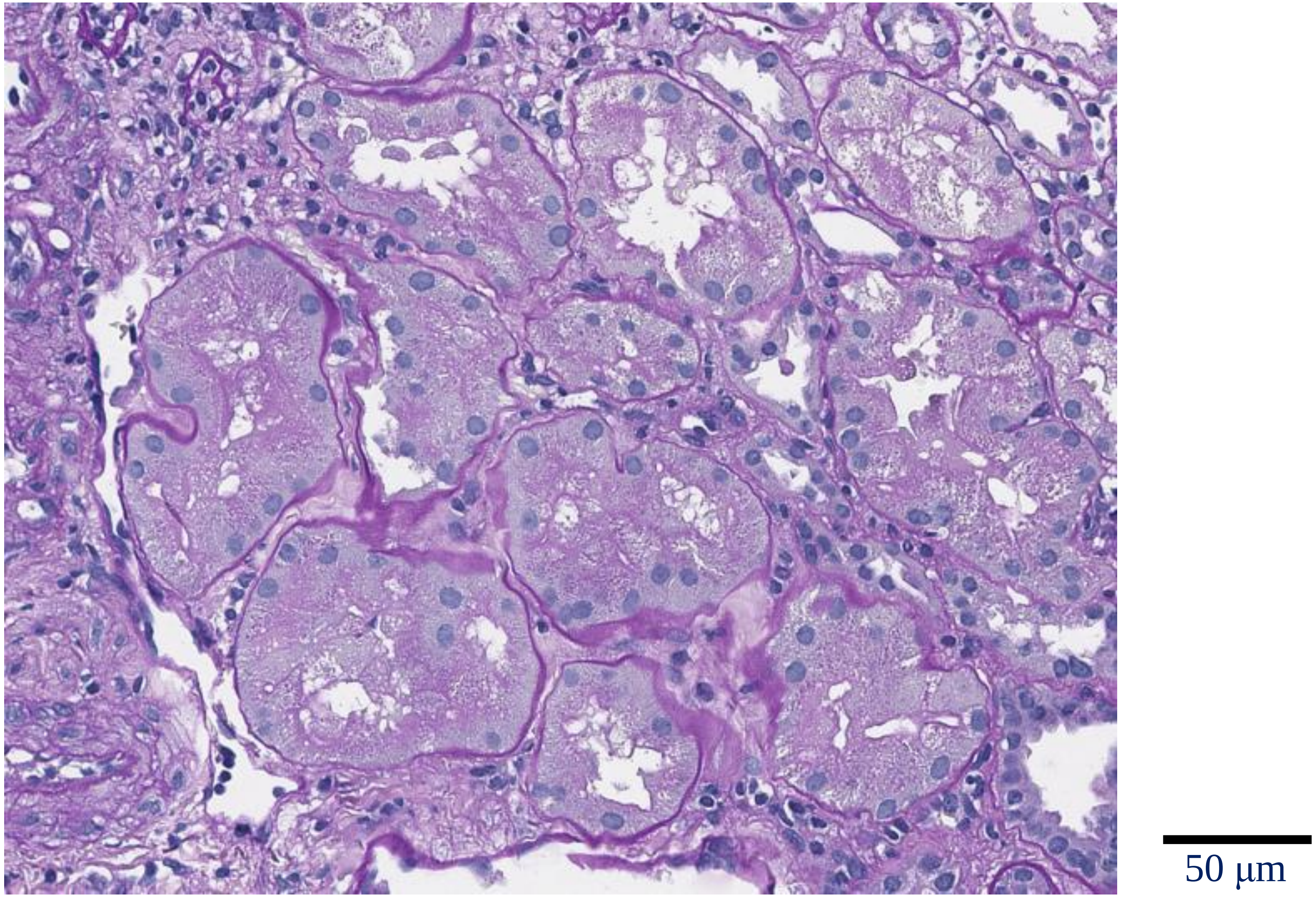

4 – Perfect
3 – Very good
2 – Good enough
1 – Not acceptable

	Stain quality score	Nuclear detail	Cytoplasmic detail	Basement membrane detail
Score				

Stain-transformed image #4

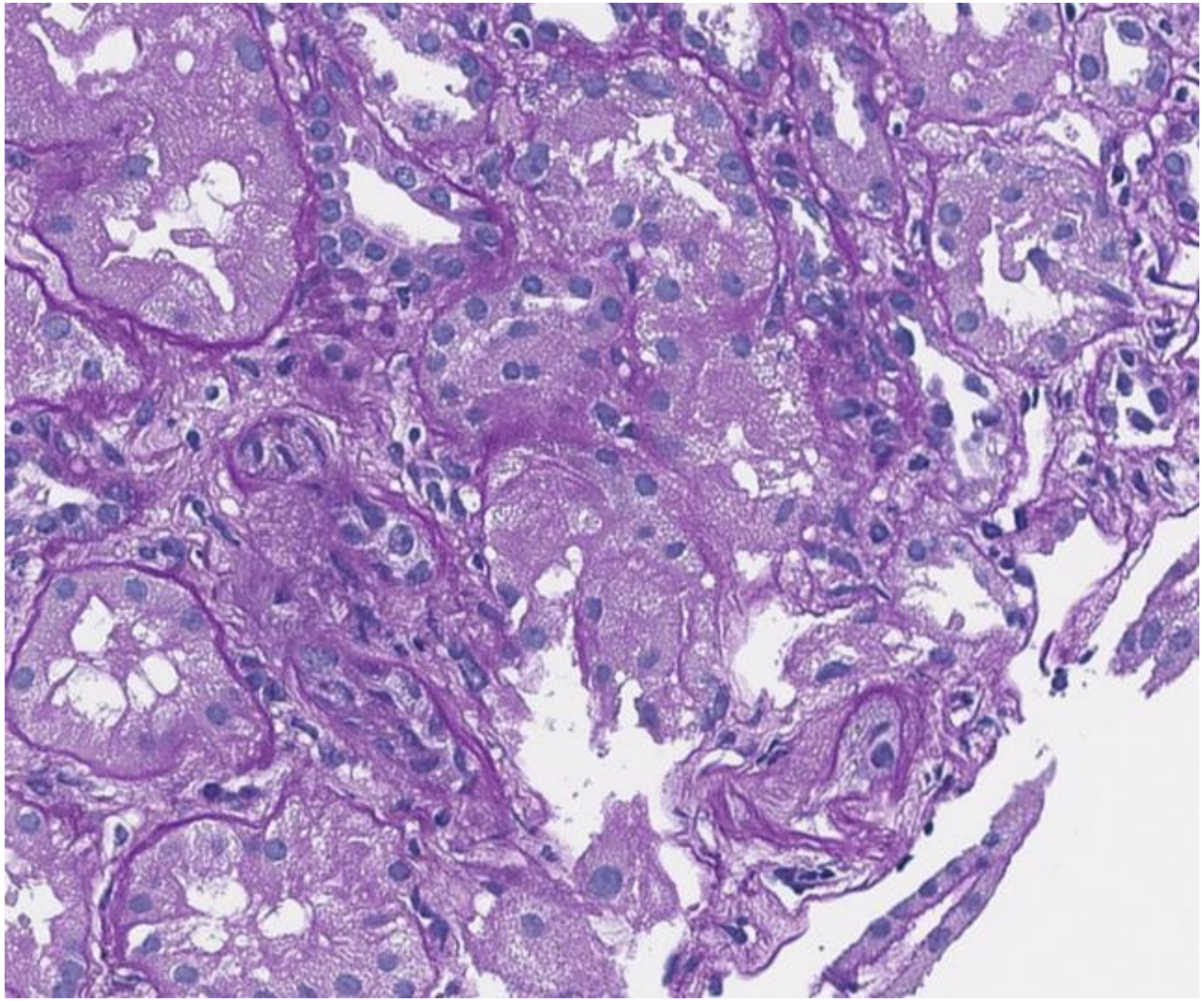

50 µm

4 – Perfect
3 – Very good
2 – Good enough
1 – Not acceptable

	Stain quality score	Nuclear detail	Cytoplasmic detail	Basement membrane detail
Score				

Histochemically stained image #4

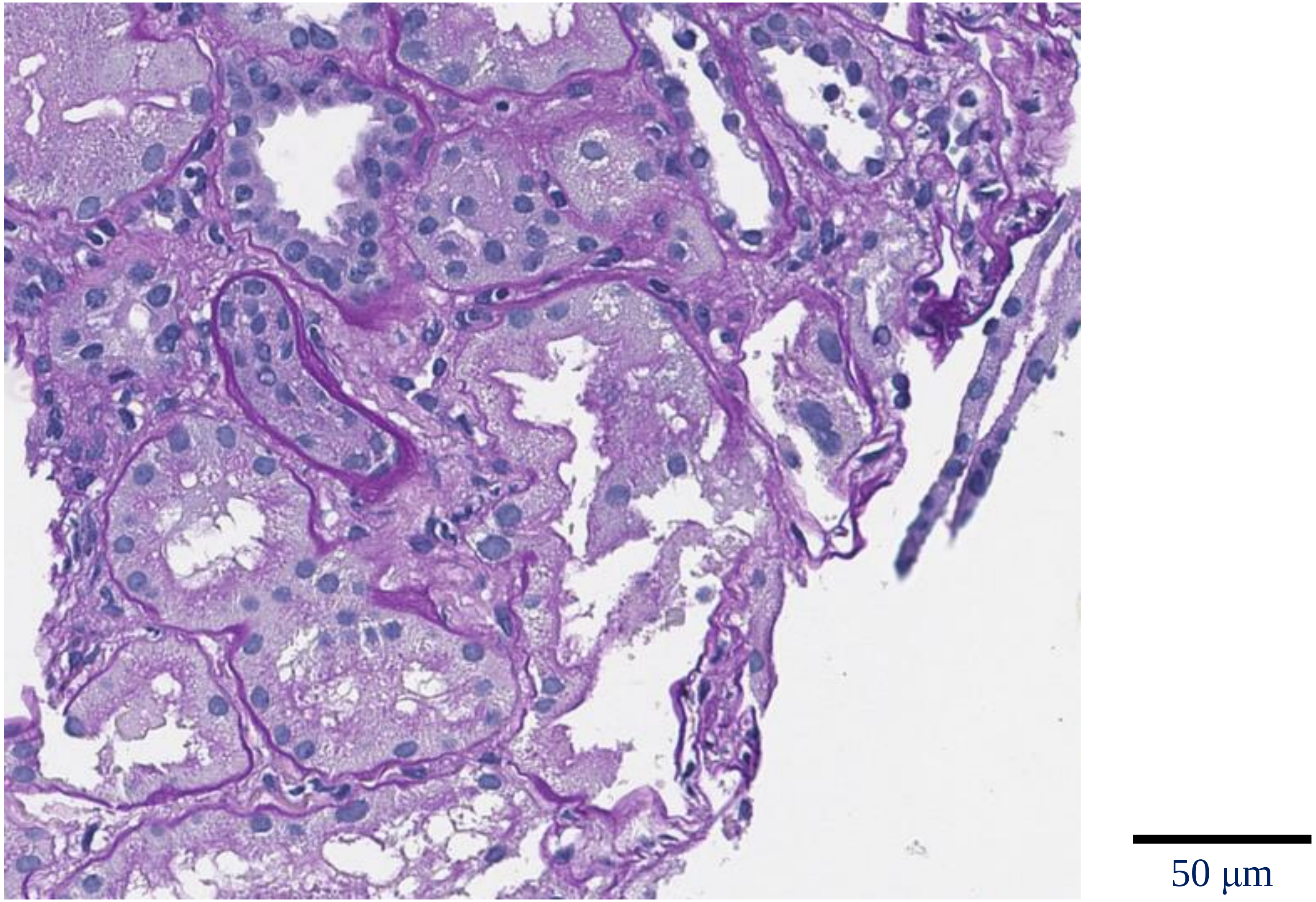

4 – Perfect
3 – Very good
2 – Good enough
1 – Not acceptable

	Stain quality score	Nuclear detail	Cytoplasmic detail	Basement membrane detail
Score				

Stain-transformed image #5

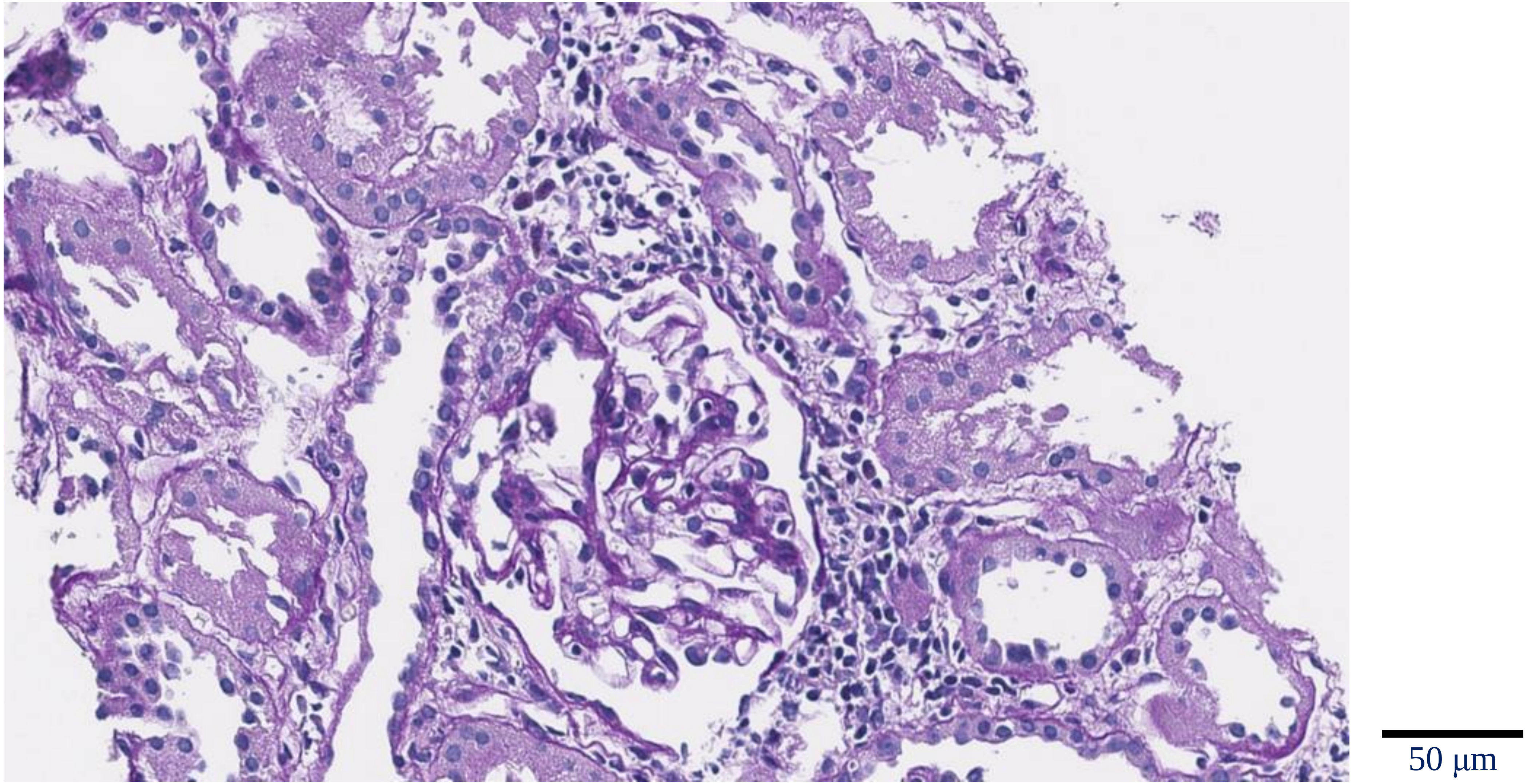

4 – Perfect
3 – Very good
2 – Good enough
1 – Not acceptable

	Stain quality score	Nuclear detail	Cytoplasmic detail	Basement membrane detail
Score				

Histochemically stained image #5

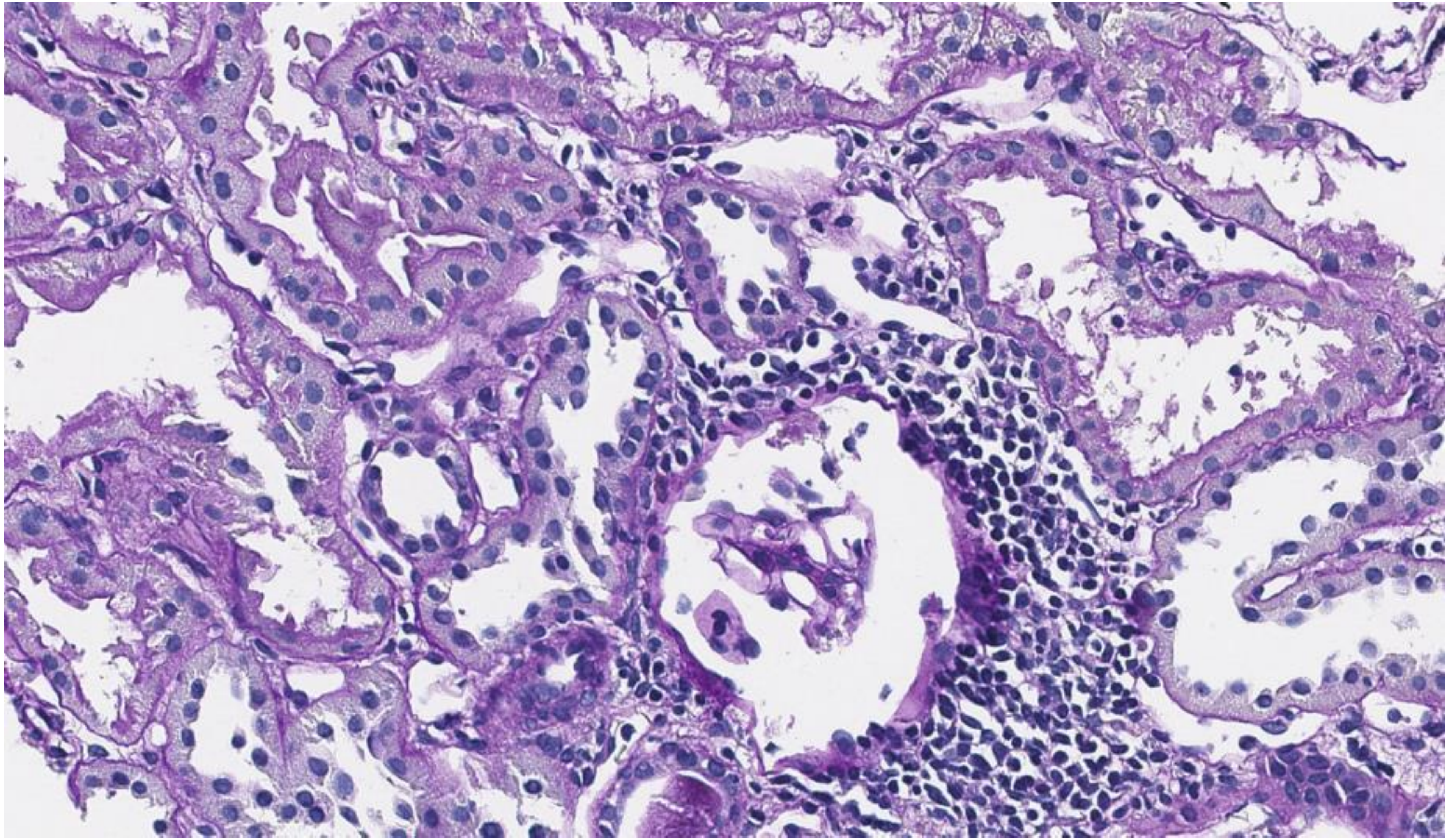

50 µm

4 – Perfect
3 – Very good
2 – Good enough
1 – Not acceptable

	Stain quality score	Nuclear detail	Cytoplasmic detail	Basement membrane detail
Score				

Stain-transformed image #6

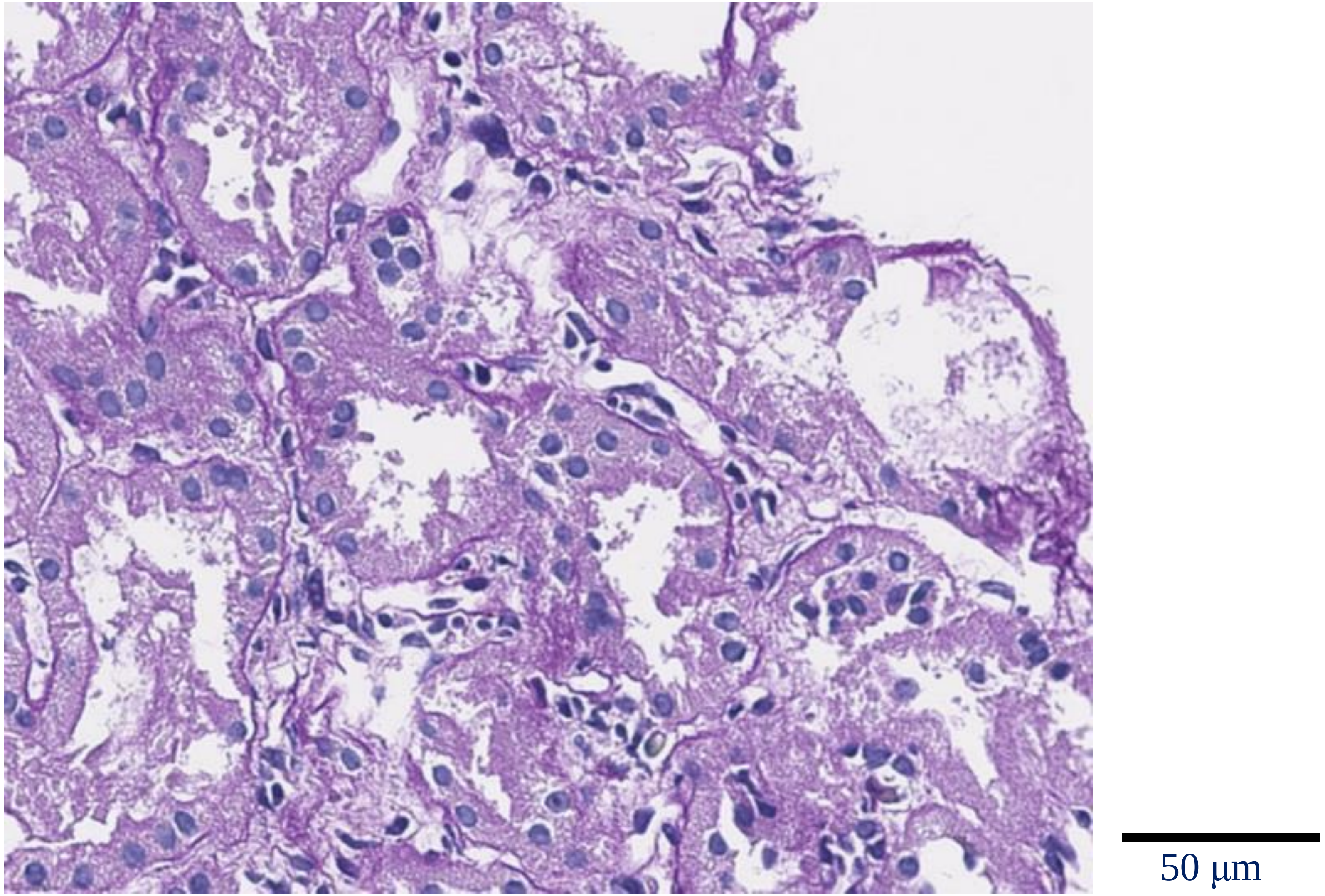

4 – Perfect
3 – Very good
2 – Good enough
1 – Not acceptable

	Stain quality score	Nuclear detail	Cytoplasmic detail	Basement membrane detail
Score				

Histochemically stained image #6

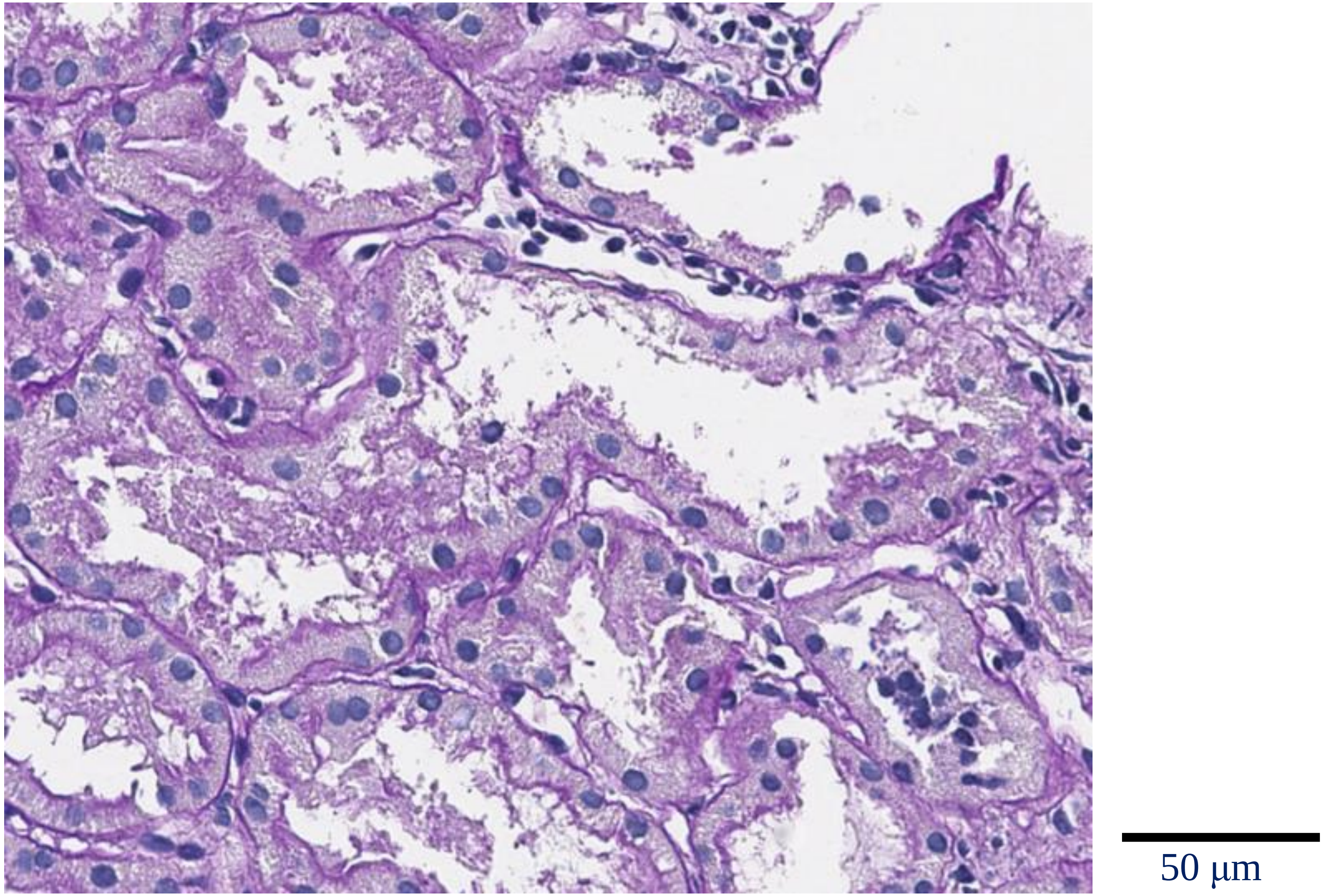

4 – Perfect
3 – Very good
2 – Good enough
1 – Not acceptable

	Stain quality score	Nuclear detail	Cytoplasmic detail	Basement membrane detail
Score				

Stain-transformed image #7

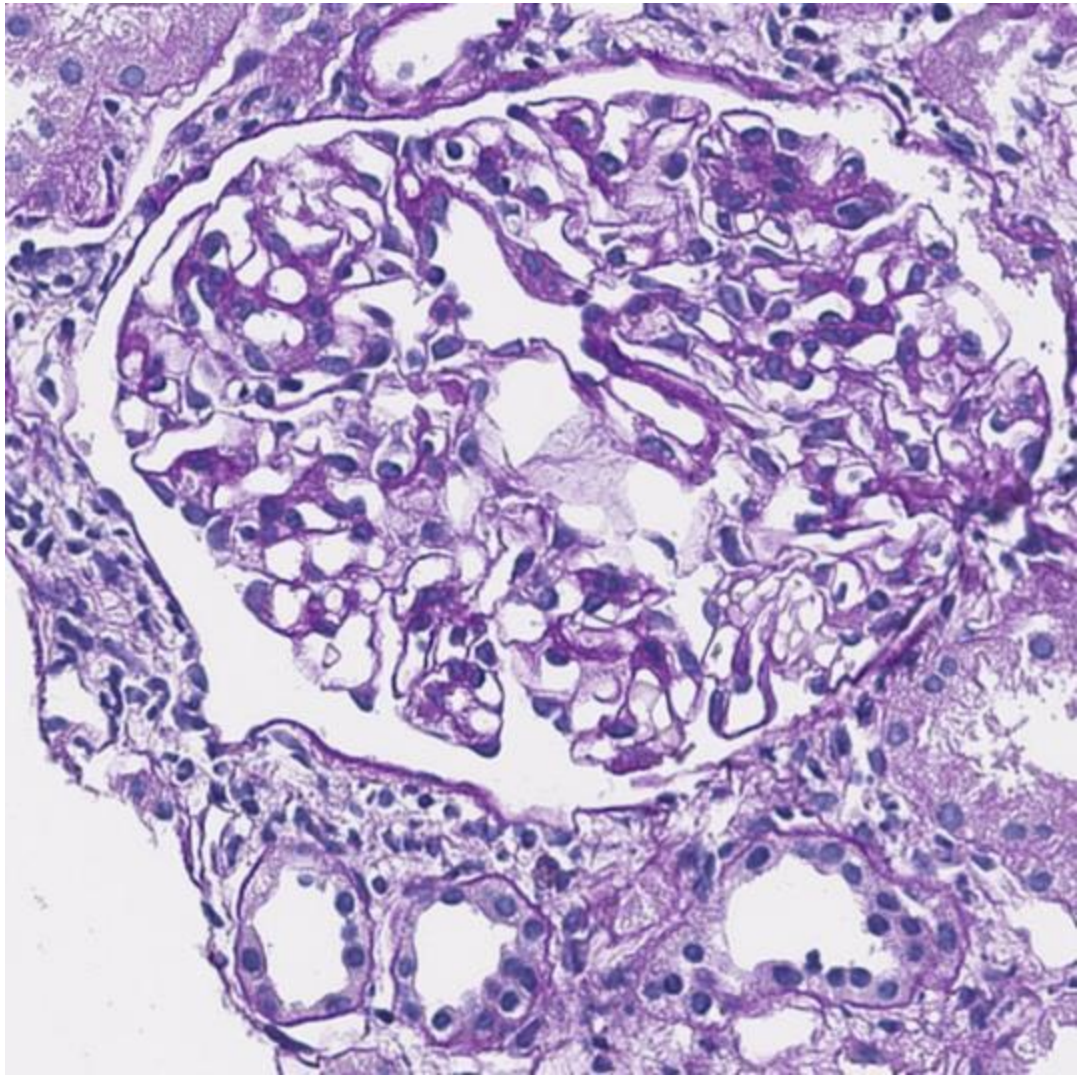

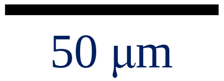

4 – Perfect
3 – Very good
2 – Good enough
1 – Not acceptable

	Stain quality score	Nuclear detail	Cytoplasmic detail	Basement membrane detail
Score				

Histochemically stained image #7

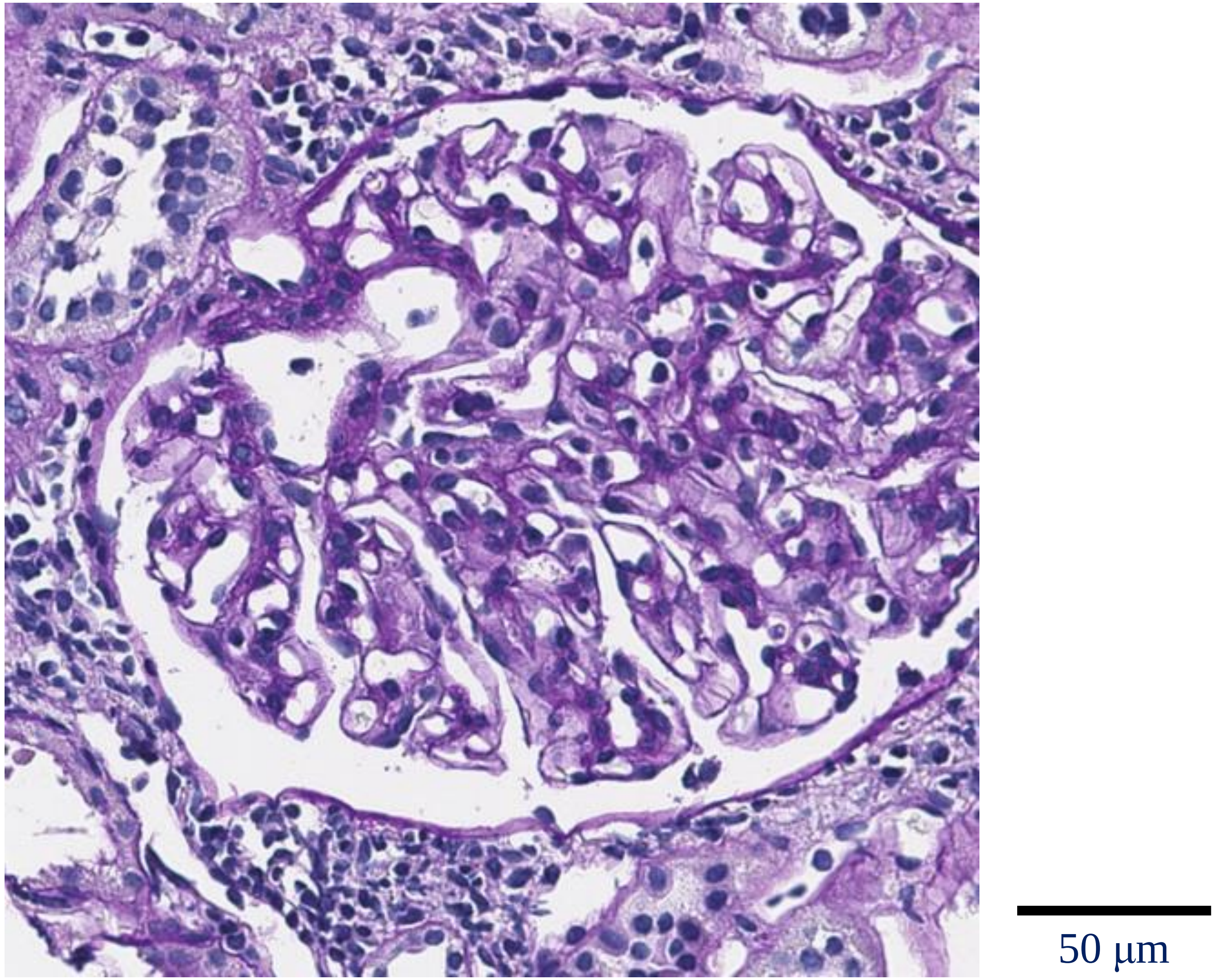

4 – Perfect
3 – Very good
2 – Good enough
1 – Not acceptable

	Stain quality score	Nuclear detail	Cytoplasmic detail	Basement membrane detail
Score				

Stain-transformed image #8

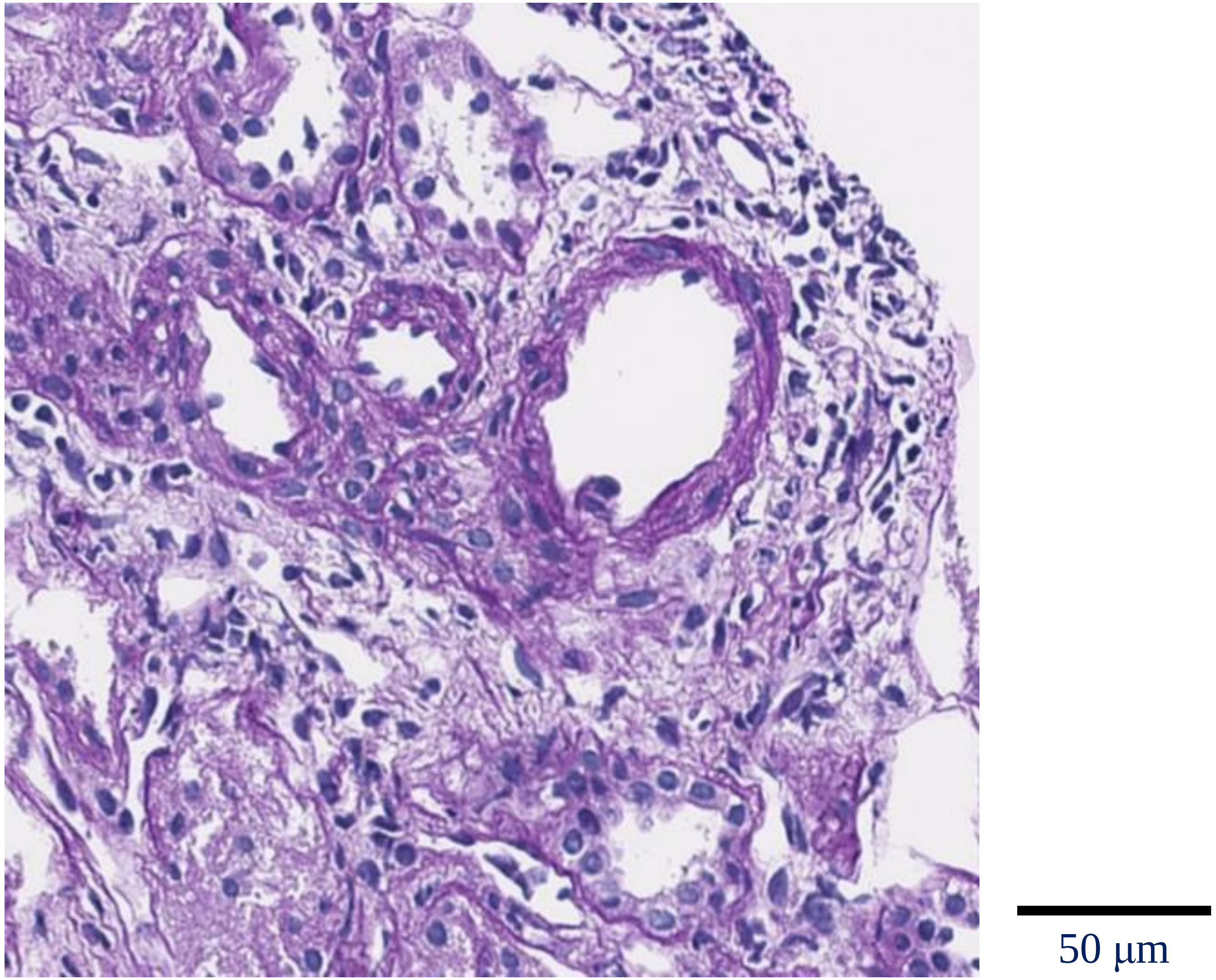

4 – Perfect
3 – Very good
2 – Good enough
1 – Not acceptable

	Stain quality score	Nuclear detail	Cytoplasmic detail	Basement membrane detail
Score				

Histochemically stained image #8

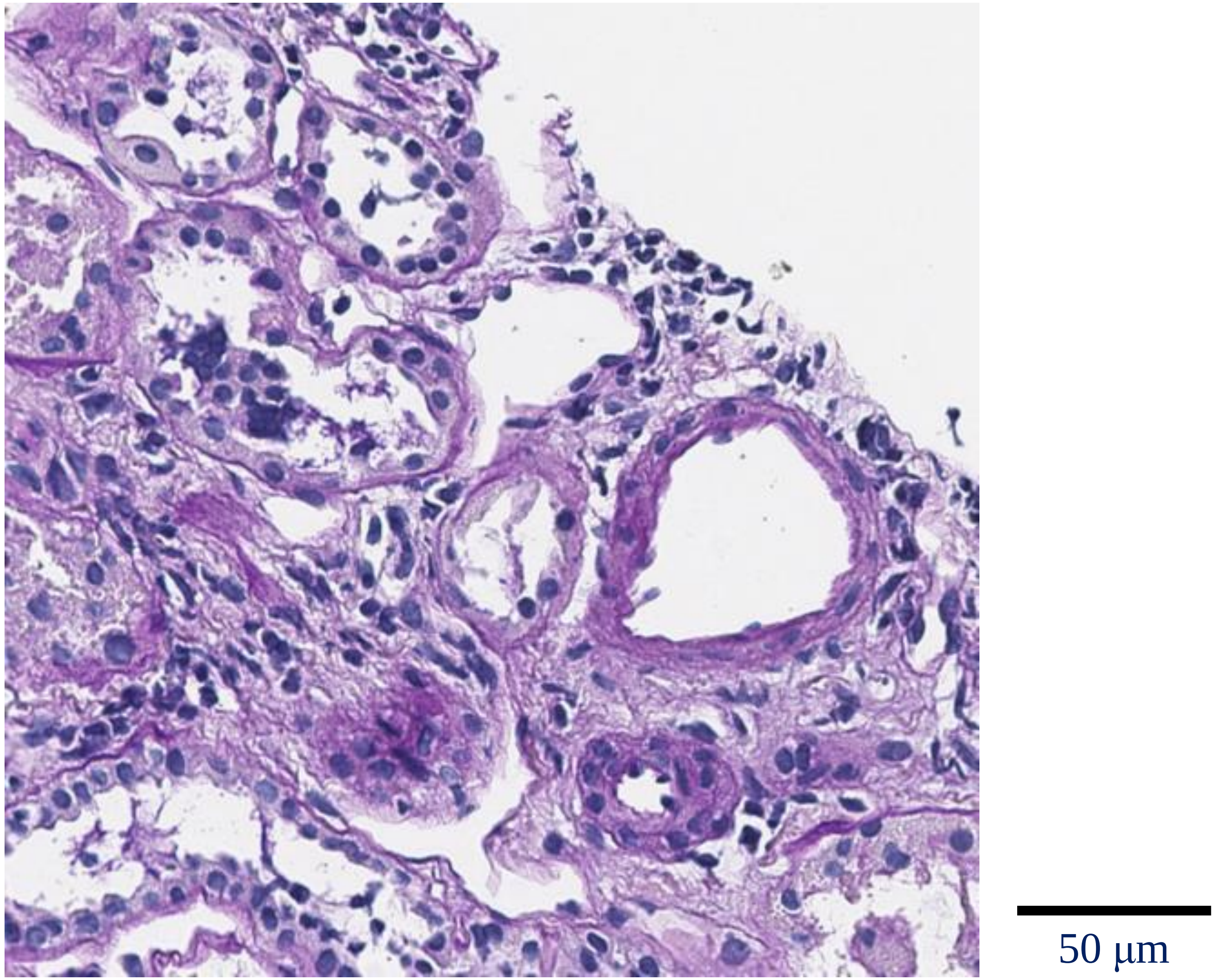

4 – Perfect
3 – Very good
2 – Good enough
1 – Not acceptable

	Stain quality score	Nuclear detail	Cytoplasmic detail	Basement membrane detail
Score				

Stain-transformed image #9

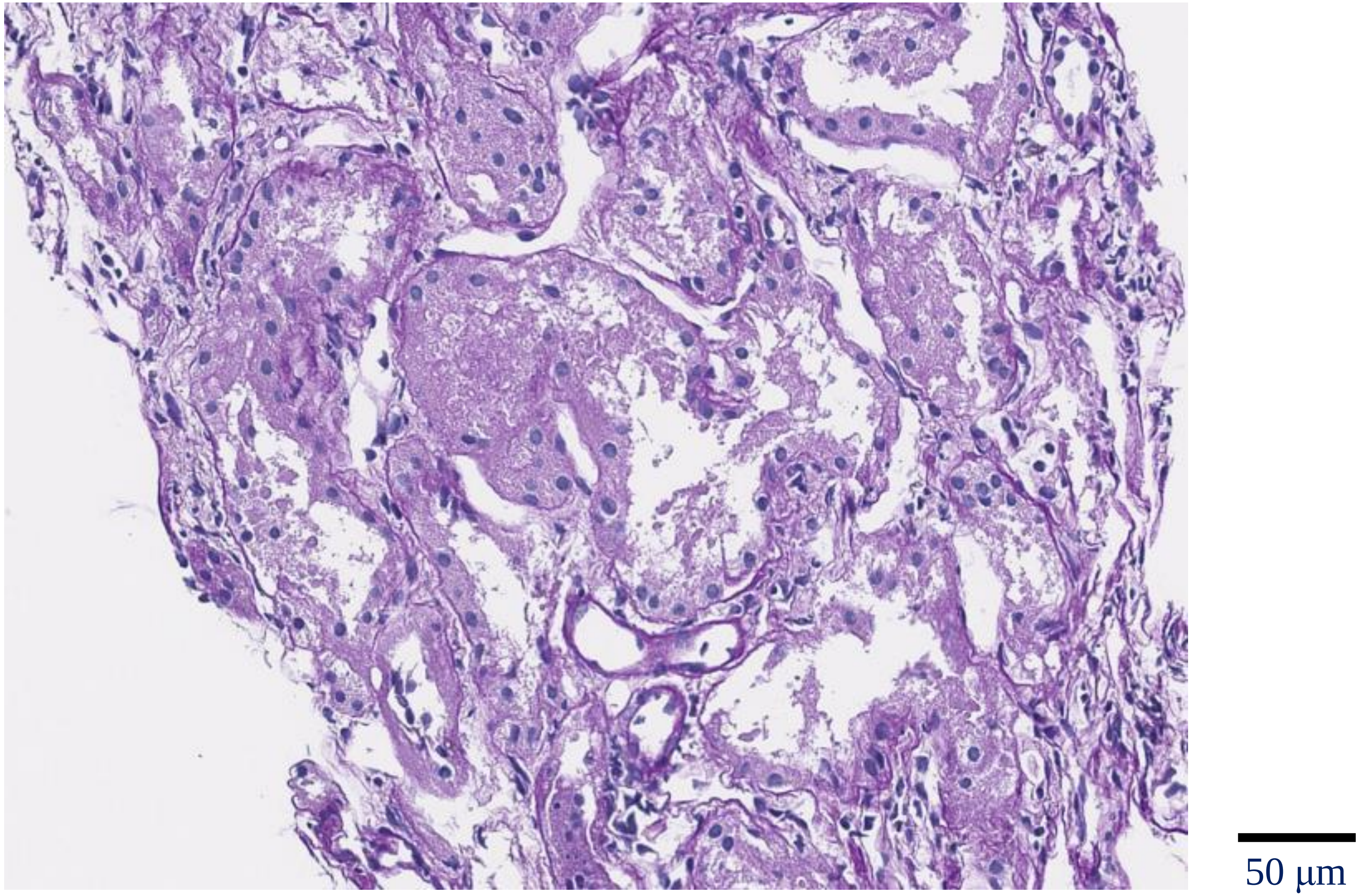

4 – Perfect

3 – Very good

2 – Good enough

1 – Not acceptable

	Stain quality score	Nuclear detail	Cytoplasmic detail	Basement membrane detail
Score				

Histochemically stained image #9

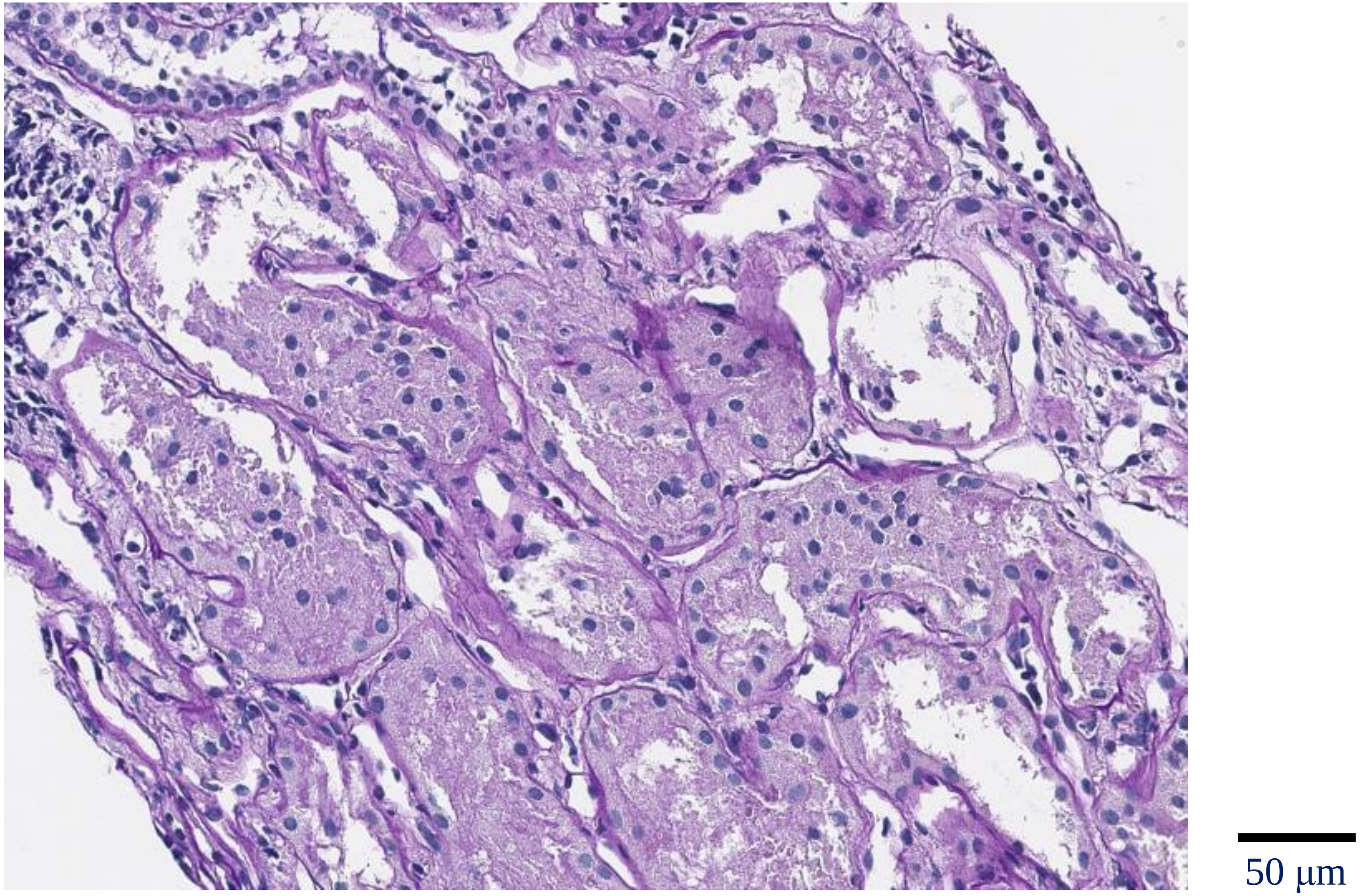

4 – Perfect

3 – Very good

2 – Good enough

1 – Not acceptable

	Stain quality score	Nuclear detail	Cytoplasmic detail	Basement membrane detail
Score				

Stain-transformed image #10

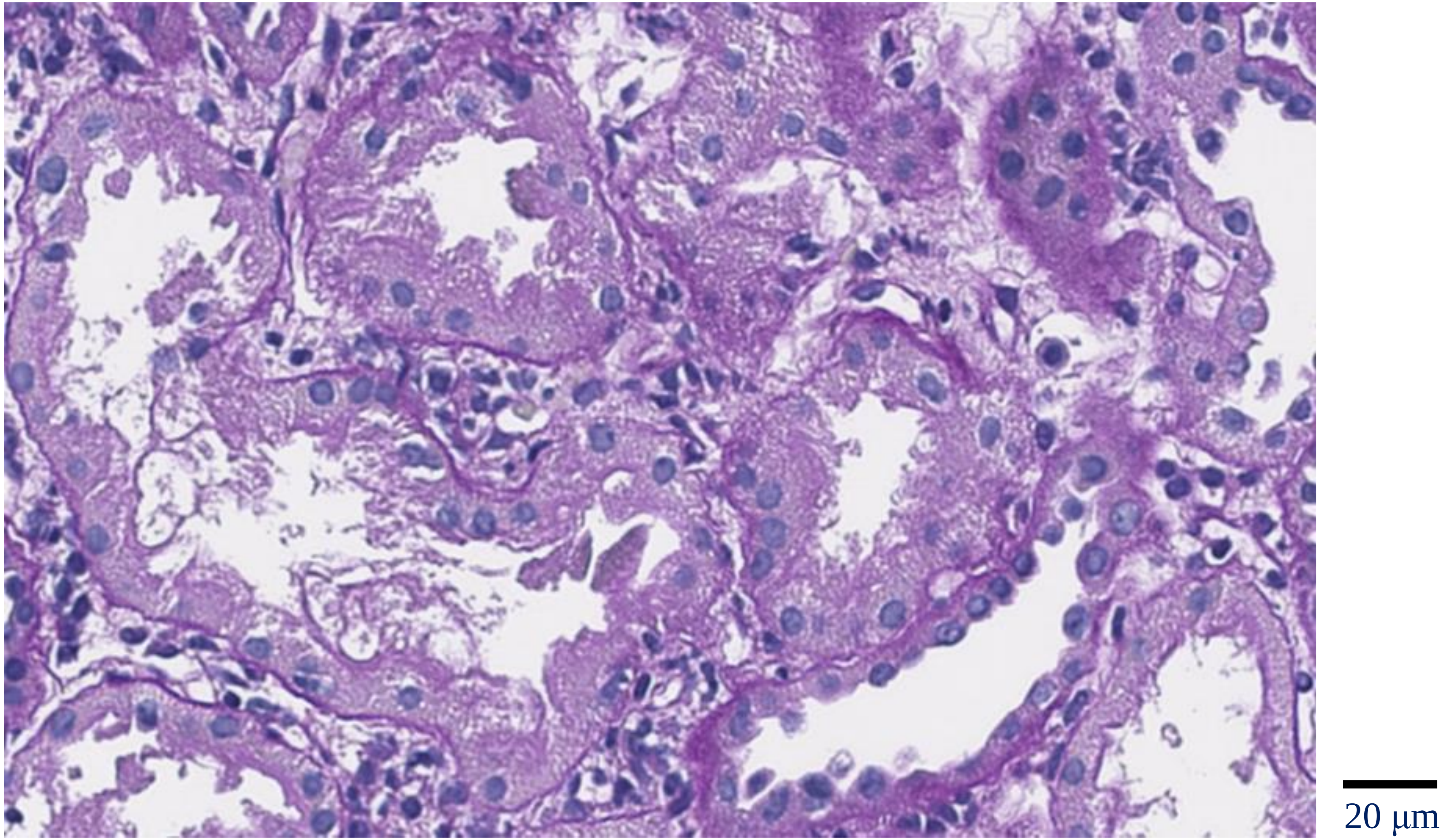

4 – Perfect
3 – Very good
2 – Good enough
1 – Not acceptable

	Stain quality score	Nuclear detail	Cytoplasmic detail	Basement membrane detail
Score				

Histochemically stained image #10

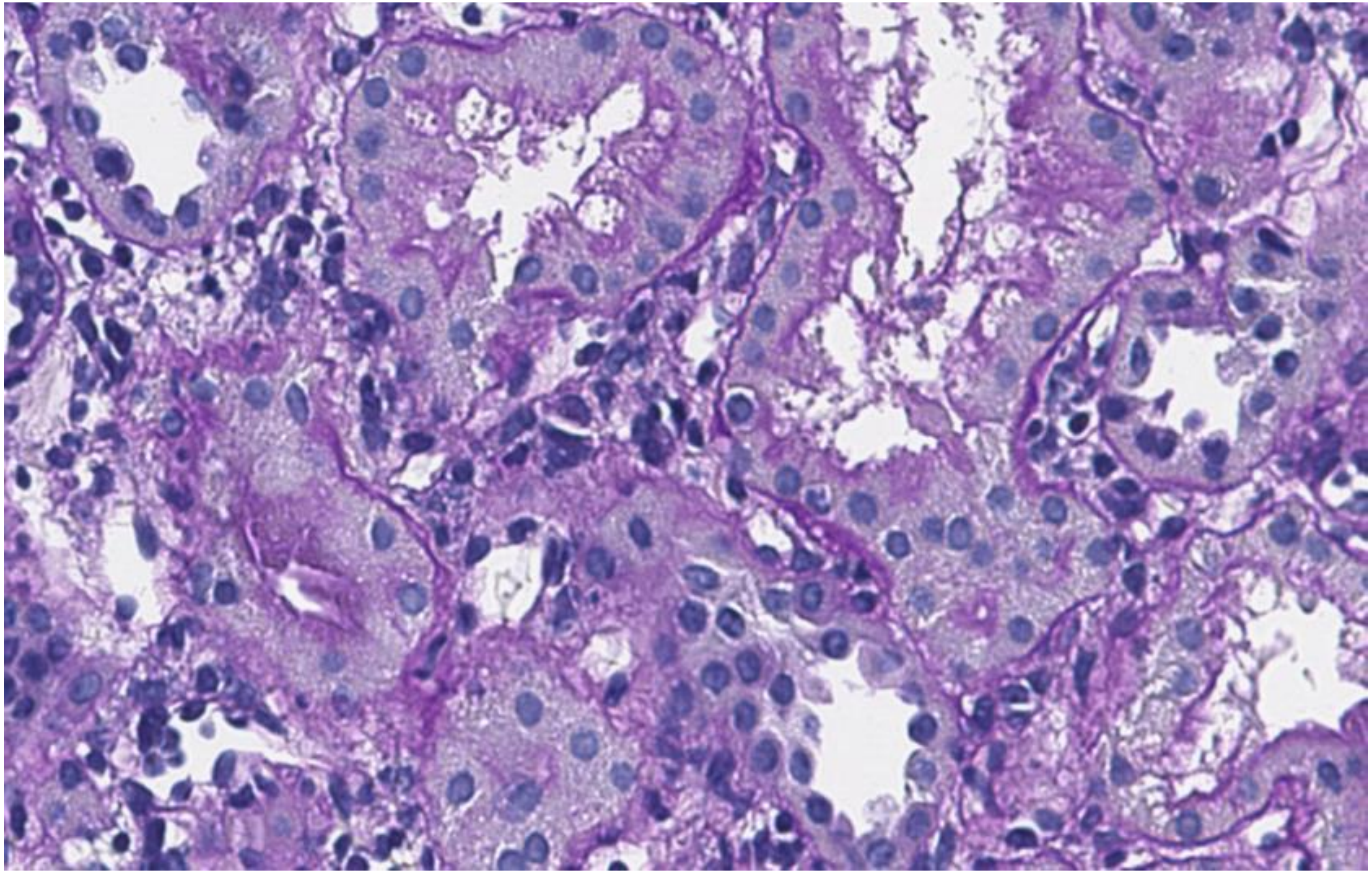

20 μm

4 – Perfect
3 – Very good
2 – Good enough
1 – Not acceptable

	Stain quality score	Nuclear detail	Cytoplasmic detail	Basement membrane detail
Score				

Stain-transformed image #11

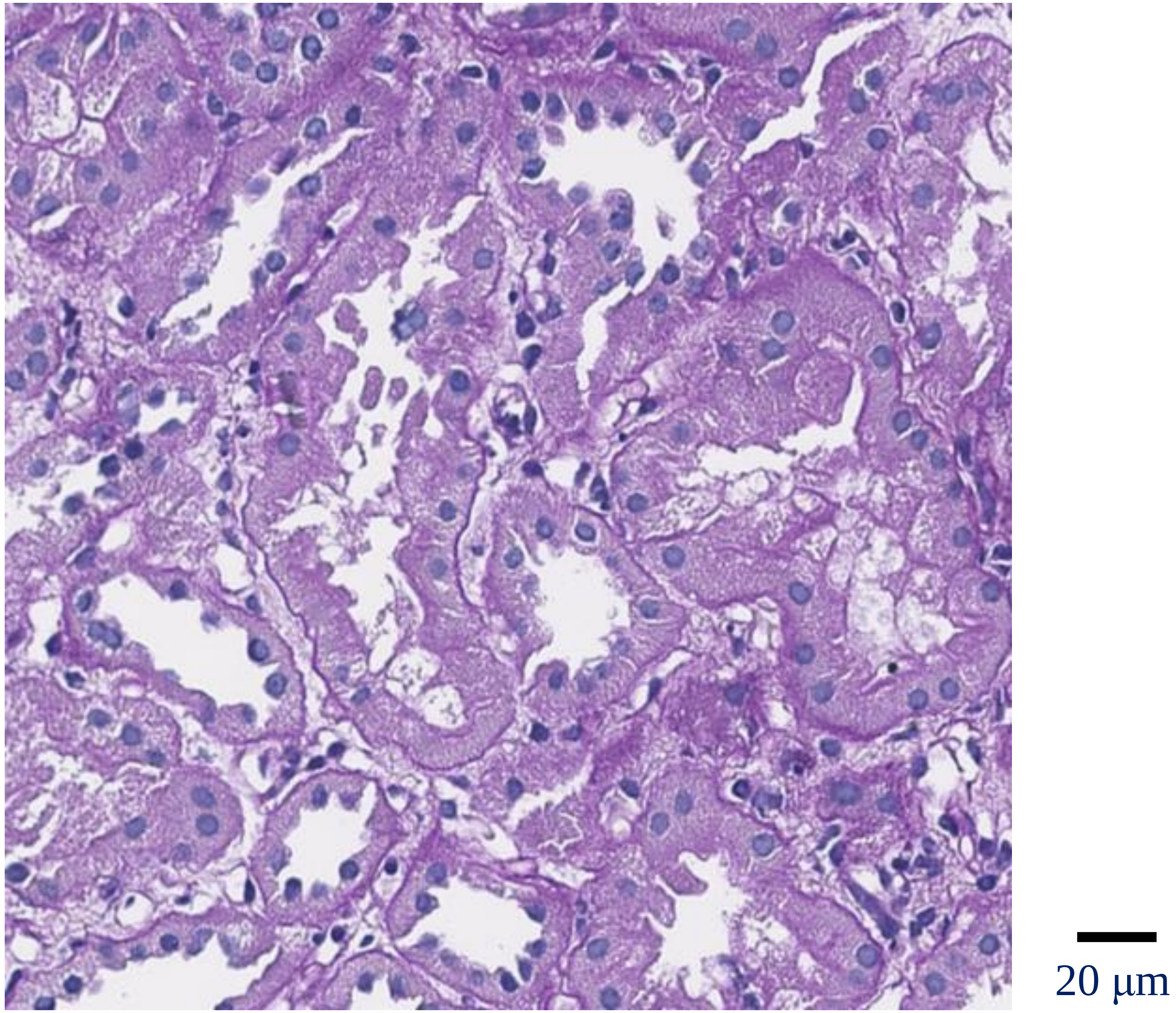

4 – Perfect
3 – Very good
2 – Good enough
1 – Not acceptable

	Stain quality score	Nuclear detail	Cytoplasmic detail	Basement membrane detail
Score				

Histochemically stained image #11

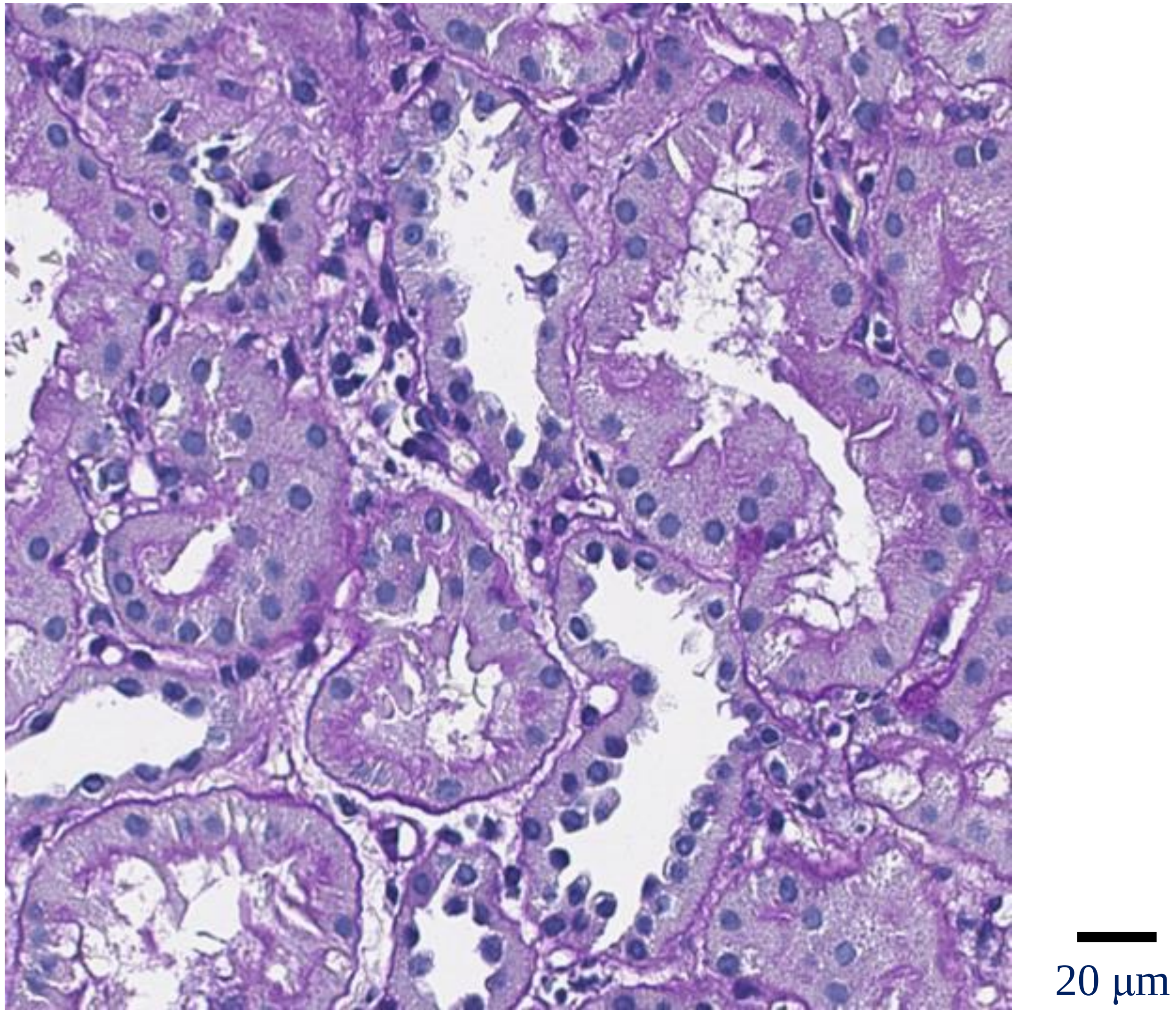

4 – Perfect
3 – Very good
2 – Good enough
1 – Not acceptable

	Stain quality score	Nuclear detail	Cytoplasmic detail	Basement membrane detail
Score				

Stain-transformed image #12

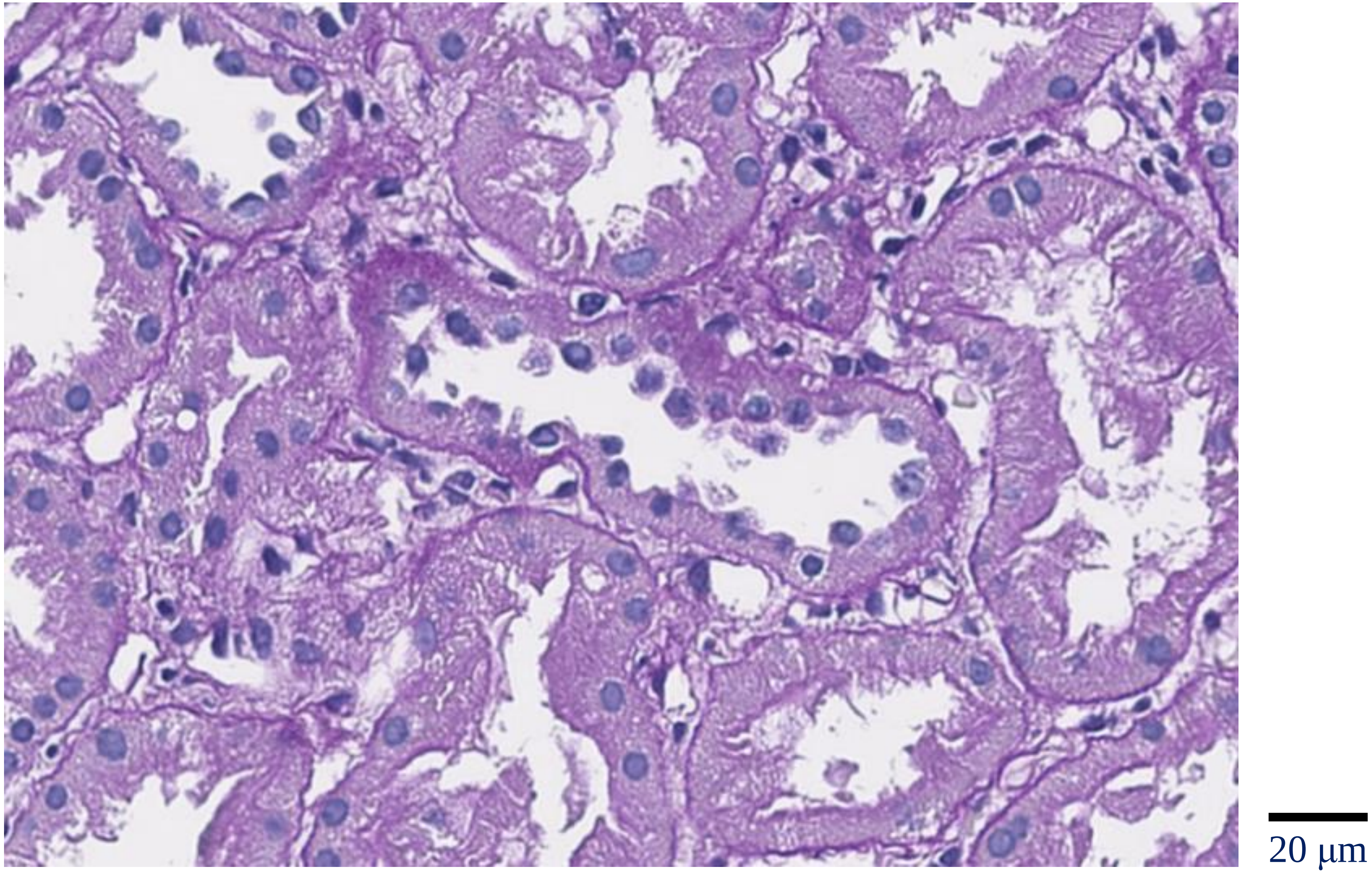

4 – Perfect
3 – Very good
2 – Good enough
1 – Not acceptable

	Stain quality score	Nuclear detail	Cytoplasmic detail	Basement membrane detail
Score				

Histochemically stained image #12

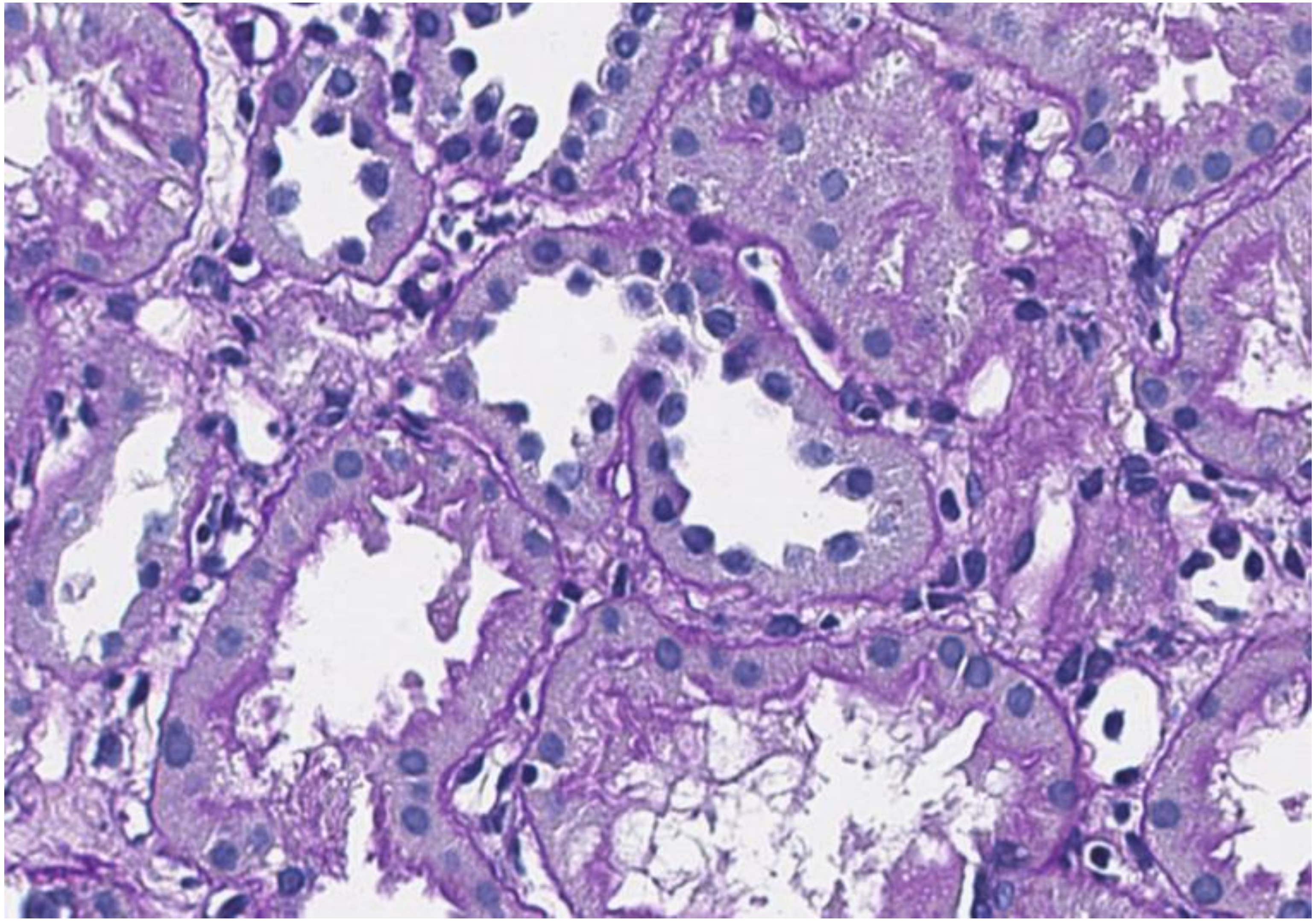

20 µm

4 – Perfect
3 – Very good
2 – Good enough
1 – Not acceptable

	Stain quality score	Nuclear detail	Cytoplasmic detail	Basement membrane detail
Score				

Stain-transformed image #13

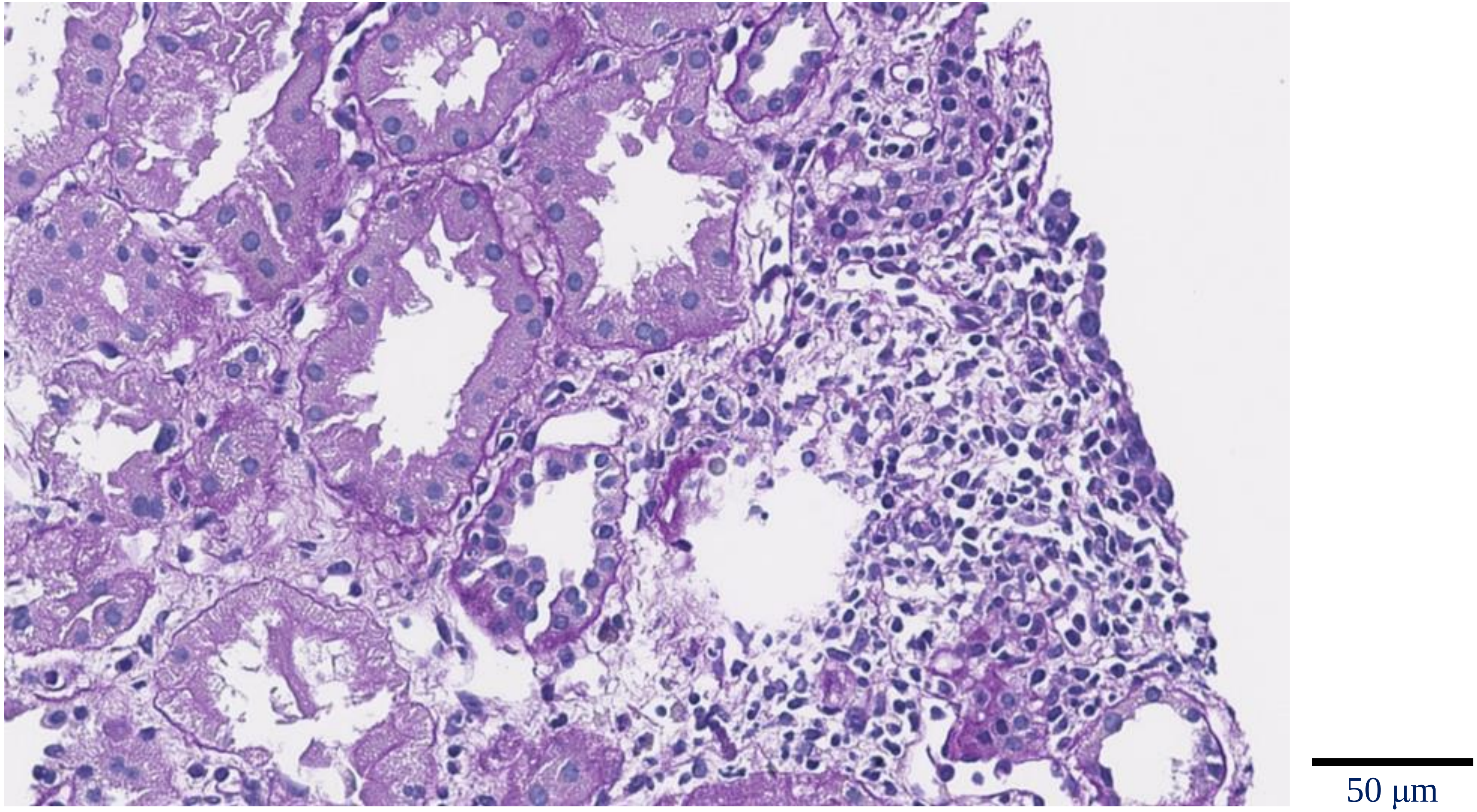

4 – Perfect
3 – Very good
2 – Good enough
1 – Not acceptable

	Stain quality score	Nuclear detail	Cytoplasmic detail	Basement membrane detail
Score				

Histochemically stained image #13

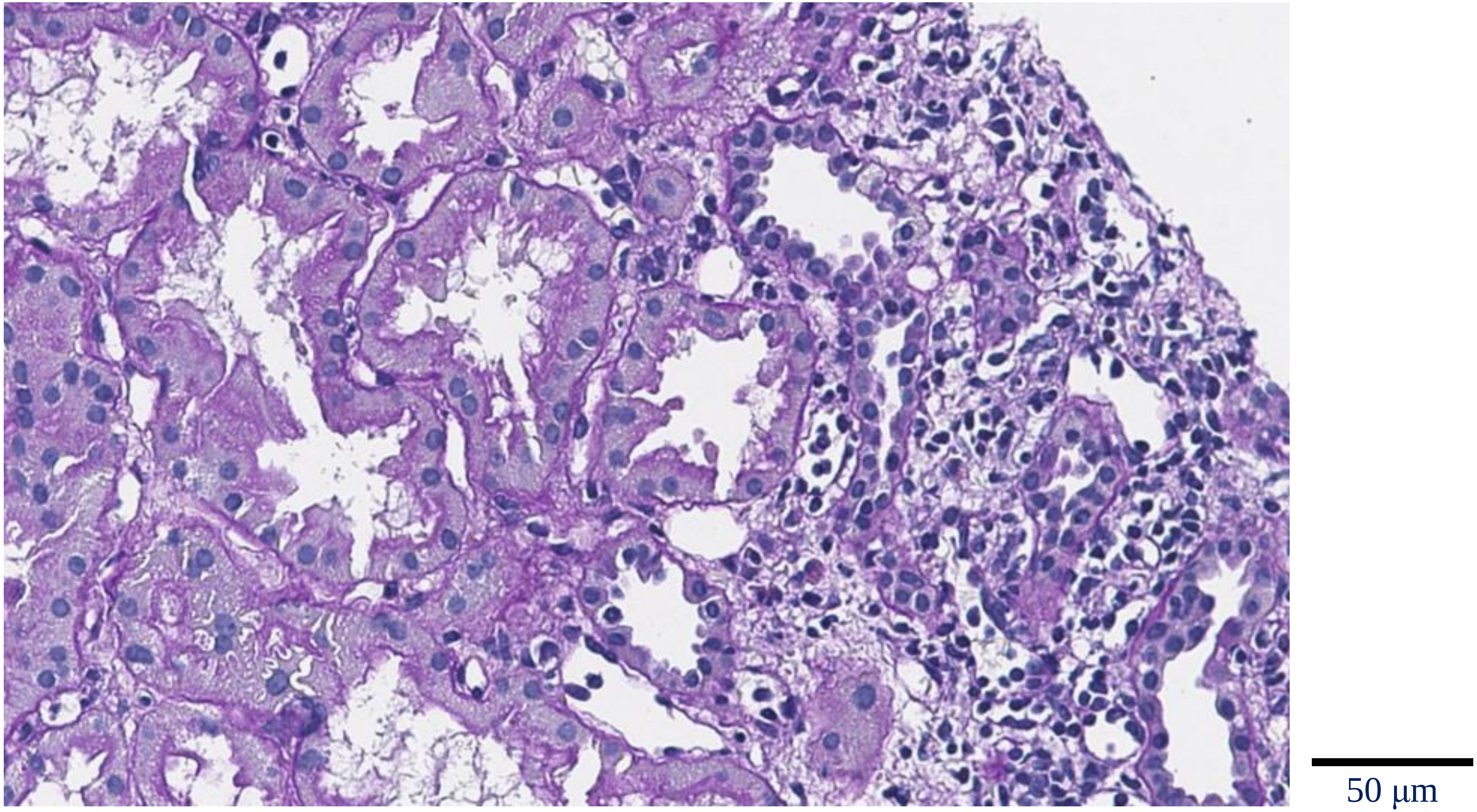

4 – Perfect
3 – Very good
2 – Good enough
1 – Not acceptable

	Stain quality score	Nuclear detail	Cytoplasmic detail	Basement membrane detail
Score				

Stain-transformed image #14

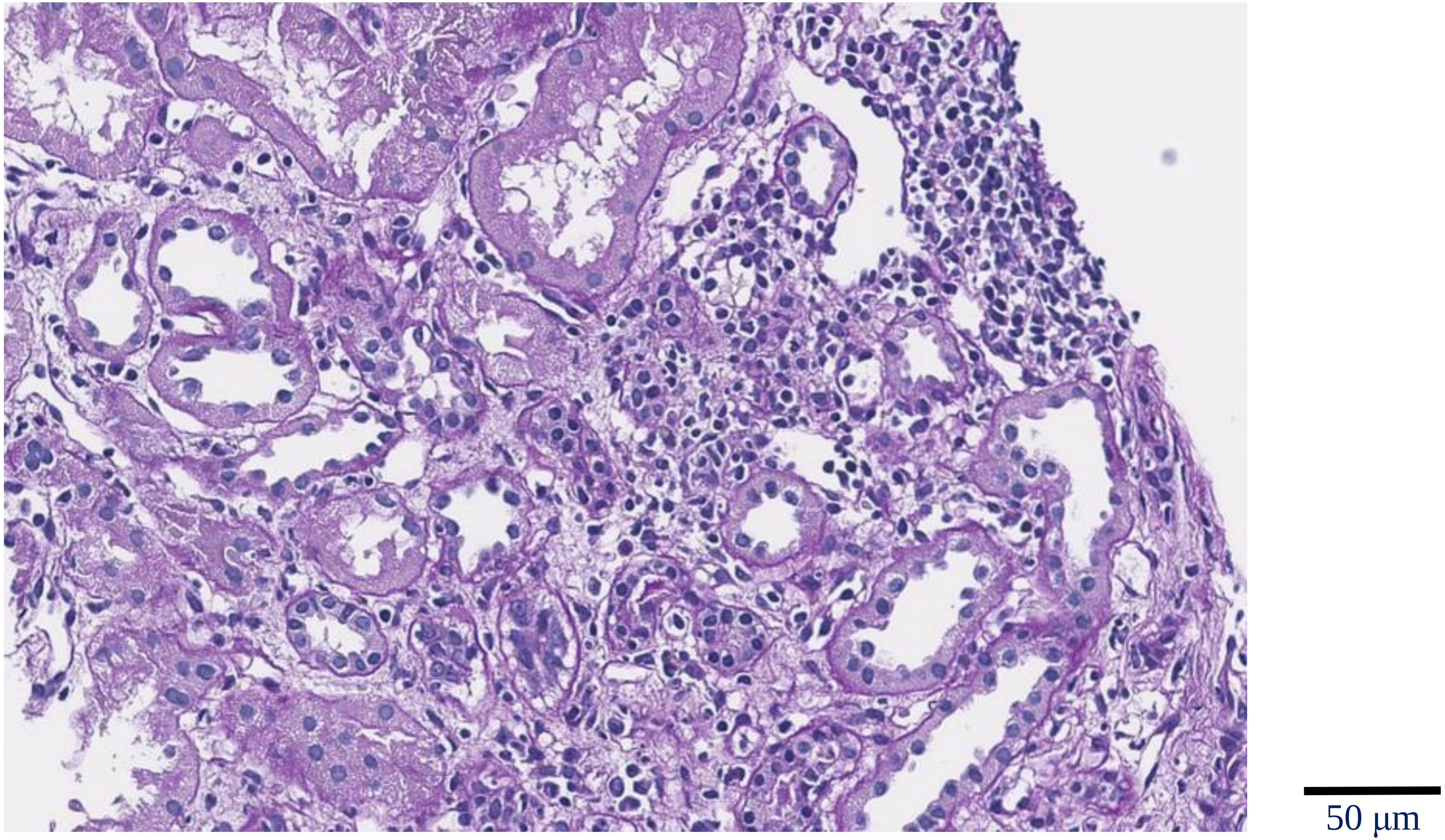

4 – Perfect
3 – Very good
2 – Good enough
1 – Not acceptable

	Stain quality score	Nuclear detail	Cytoplasmic detail	Basement membrane detail
Score				

Histochemically stained image #14

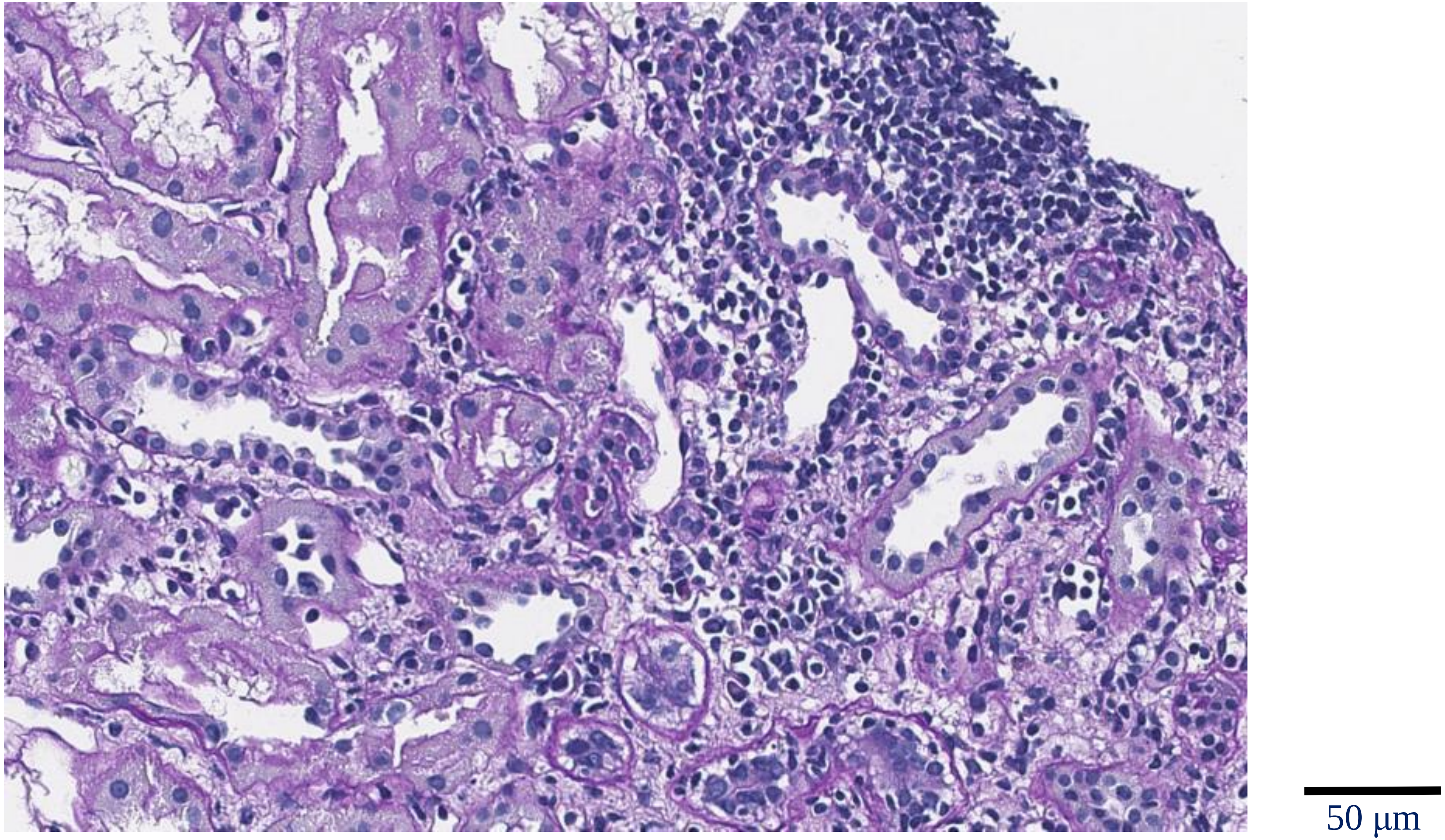

4 – Perfect
3 – Very good
2 – Good enough
1 – Not acceptable

	Stain quality score	Nuclear detail	Cytoplasmic detail	Basement membrane detail
Score				

Stain-transformed image #15

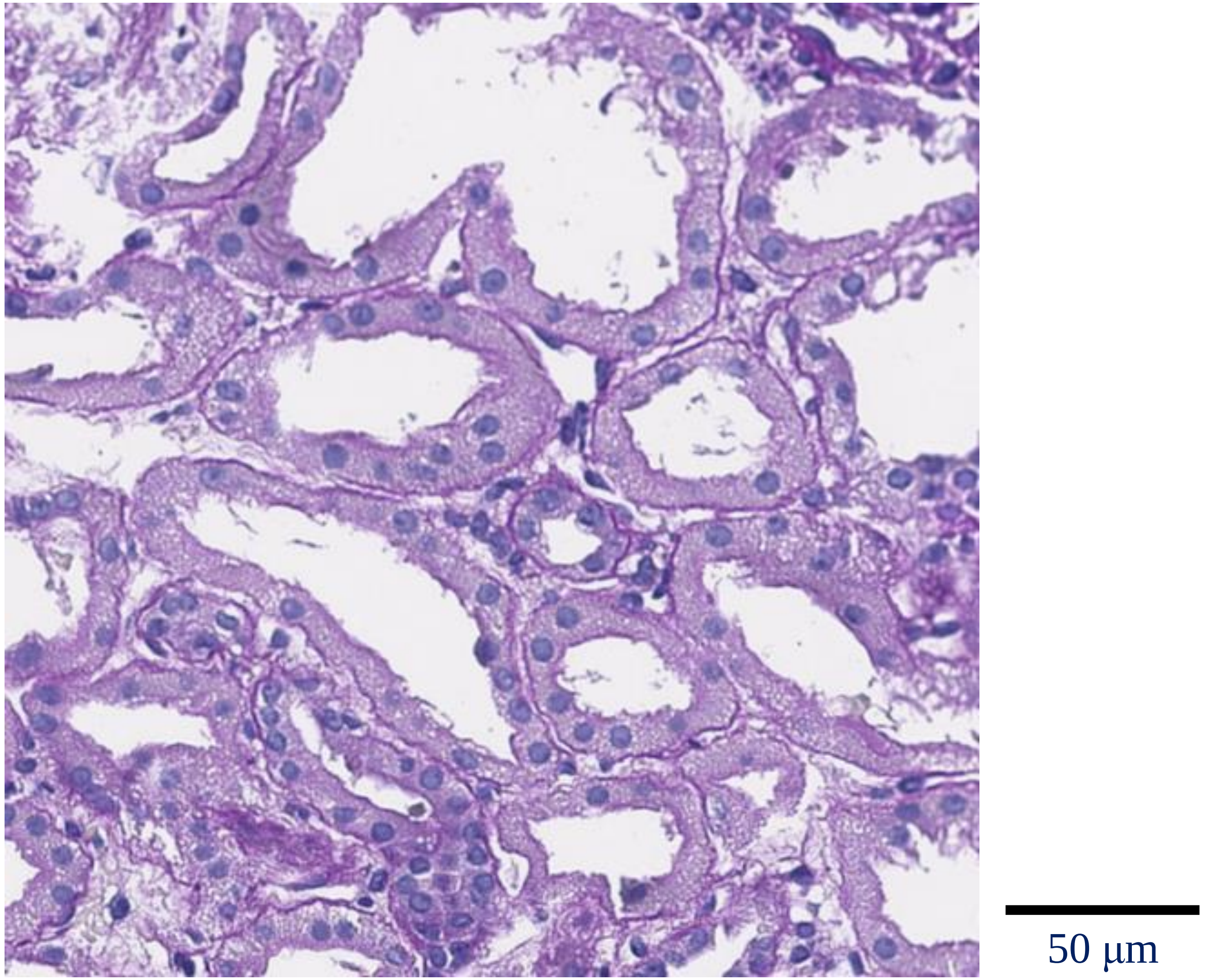

4 – Perfect
3 – Very good
2 – Good enough
1 – Not acceptable

	Stain quality score	Nuclear detail	Cytoplasmic detail	Basement membrane detail
Score				

Histochemically
stained image #15

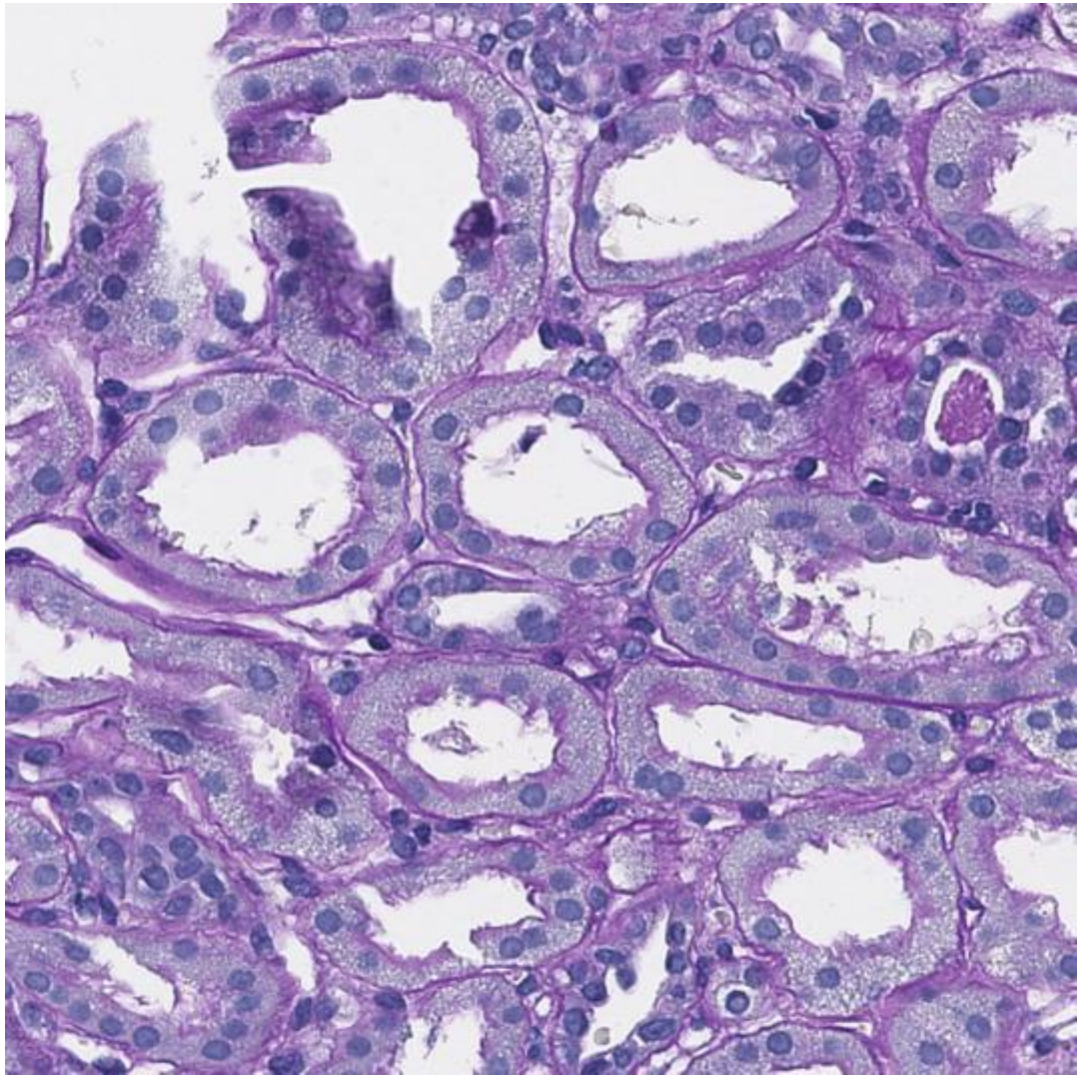

50 µm

4 – Perfect
3 – Very good
2 – Good enough
1 – Not acceptable

	Stain quality score	Nuclear detail	Cytoplasmic detail	Basement membrane detail
Score				

Stain-transformed image #16

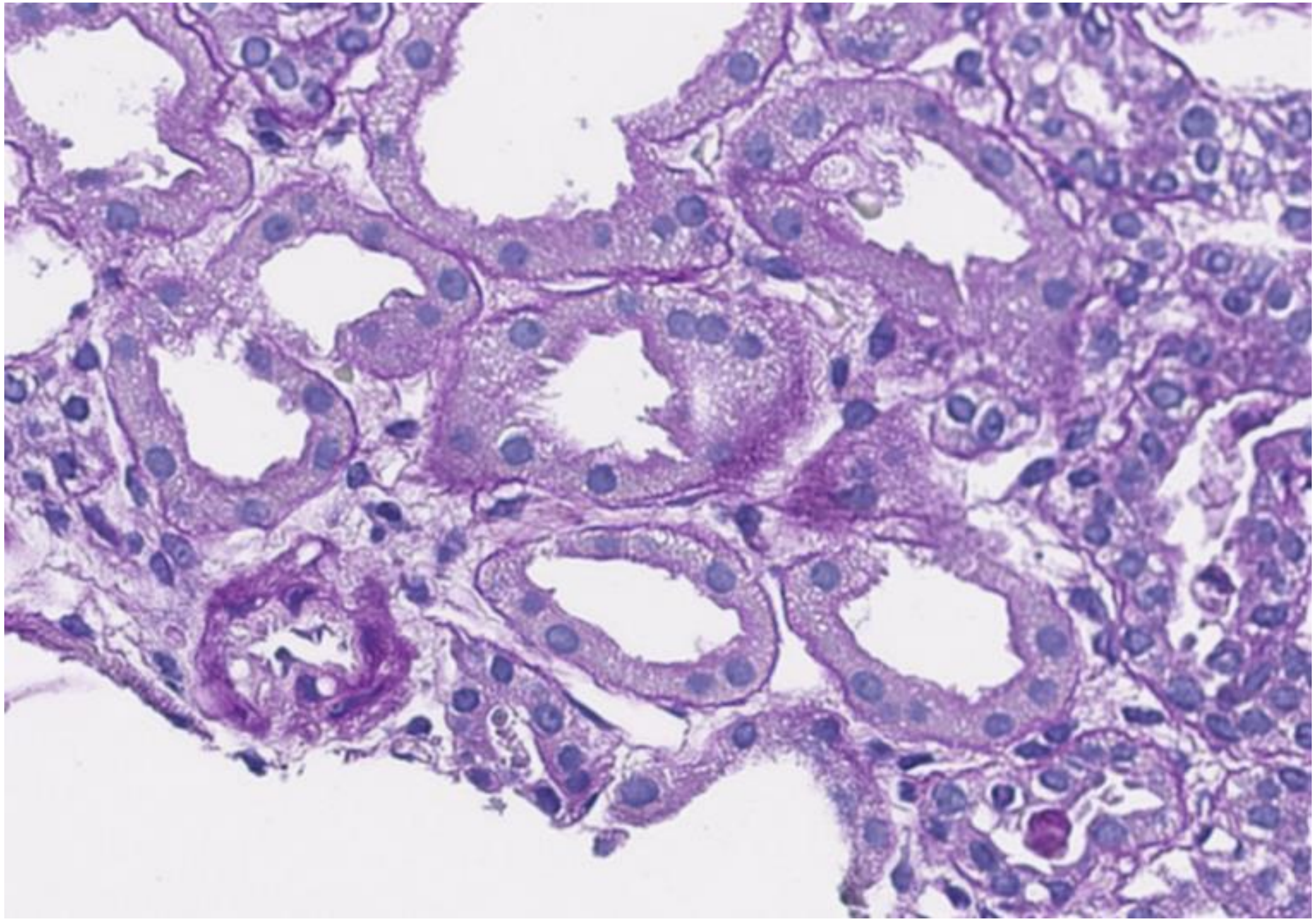

50 µm

4 – Perfect
3 – Very good
2 – Good enough
1 – Not acceptable

	Stain quality score	Nuclear detail	Cytoplasmic detail	Basement membrane detail
Score				

Histochemically stained image #16

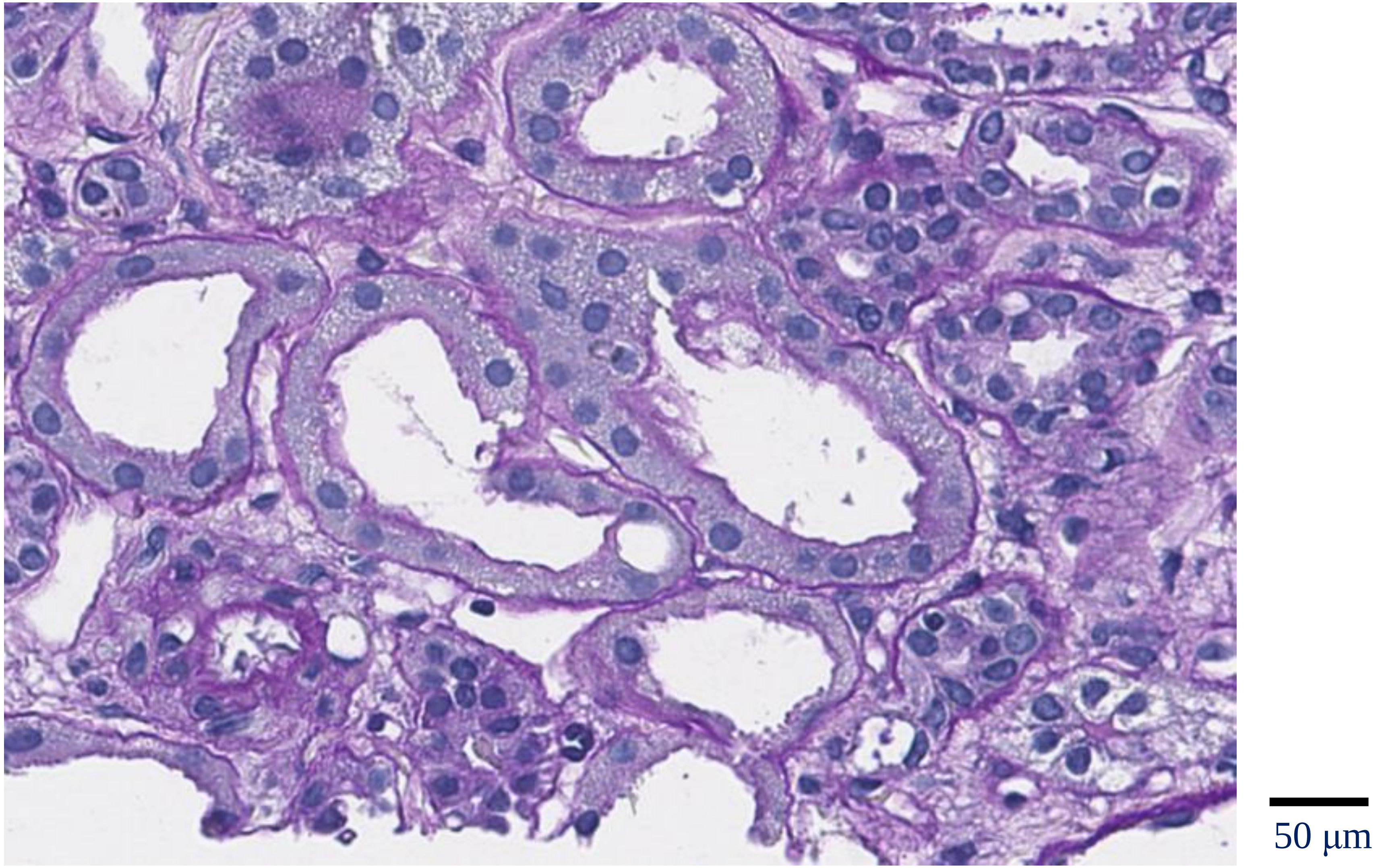

4 – Perfect

3 – Very good

2 – Good enough

1 – Not acceptable

	Stain quality score	Nuclear detail	Cytoplasmic detail	Basement membrane detail
Score				

Jones Silver Stain

Stain-transformed image #1

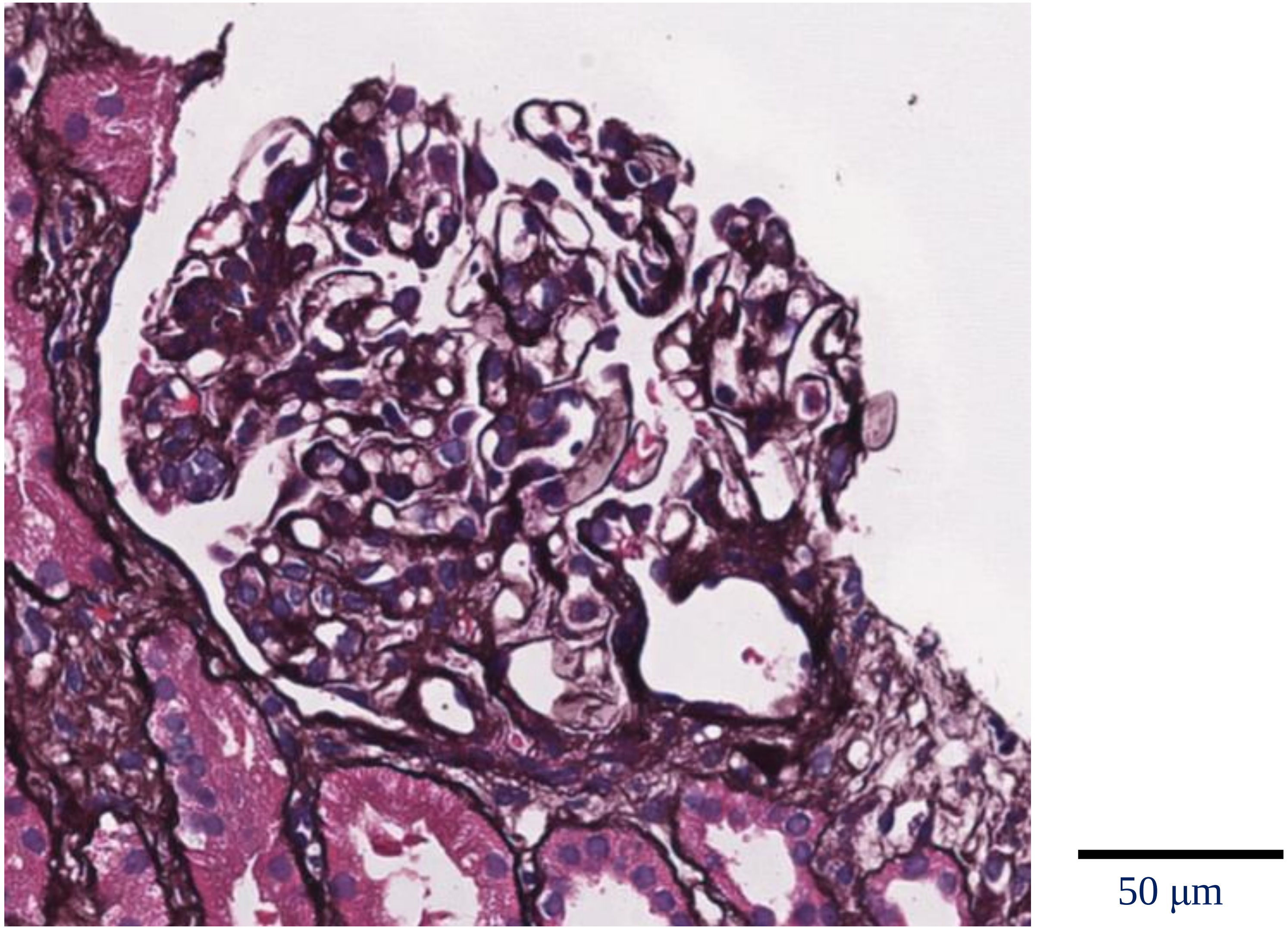

4 – Perfect
3 – Very good
2 – Good enough
1 – Not acceptable

	Stain quality score	Nuclear detail	Cytoplasmic detail	Basement membrane detail
Score				

Histochemically stained image #1

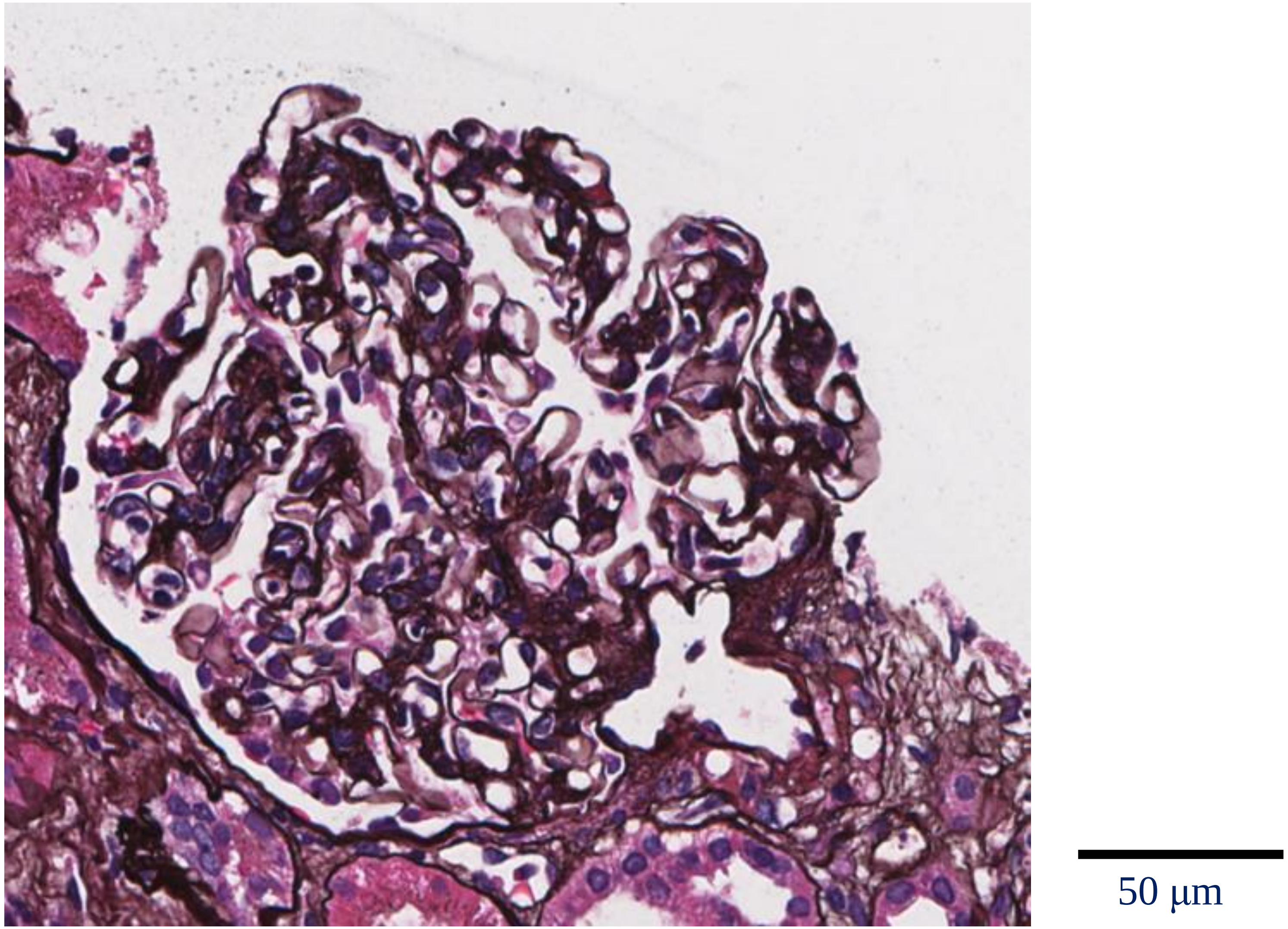

4 – Perfect
3 – Very good
2 – Good enough
1 – Not acceptable

	Stain quality score	Nuclear detail	Cytoplasmic detail	Basement membrane detail
Score				

Stain-transformed image #2

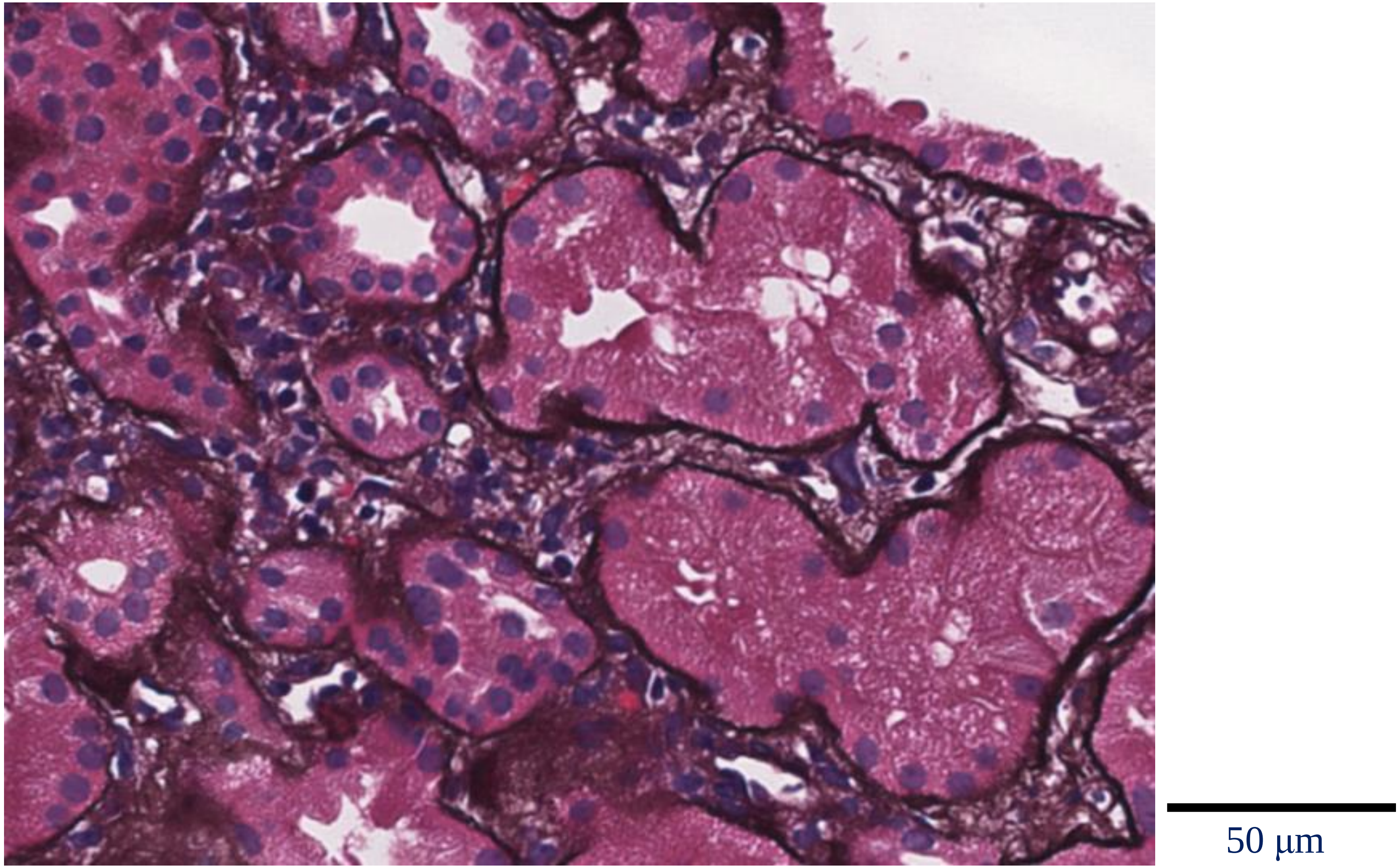

4 – Perfect
3 – Very good
2 – Good enough
1 – Not acceptable

	Stain quality score	Nuclear detail	Cytoplasmic detail	Basement membrane detail
Score				

Histochemically stained image #2

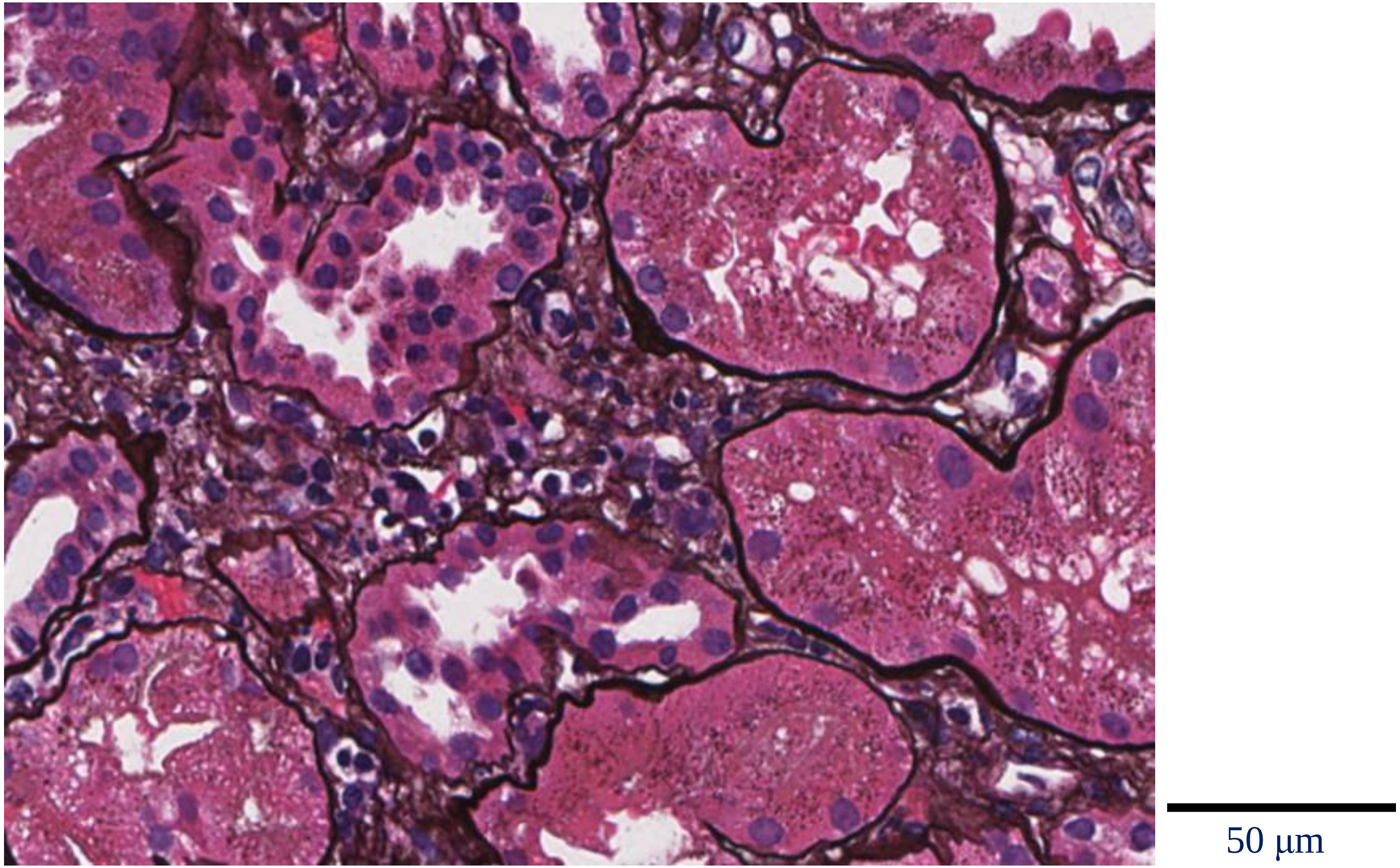

4 – Perfect
3 – Very good
2 – Good enough
1 – Not acceptable

	Stain quality score	Nuclear detail	Cytoplasmic detail	Basement membrane detail
Score				

Stain-transformed image #3

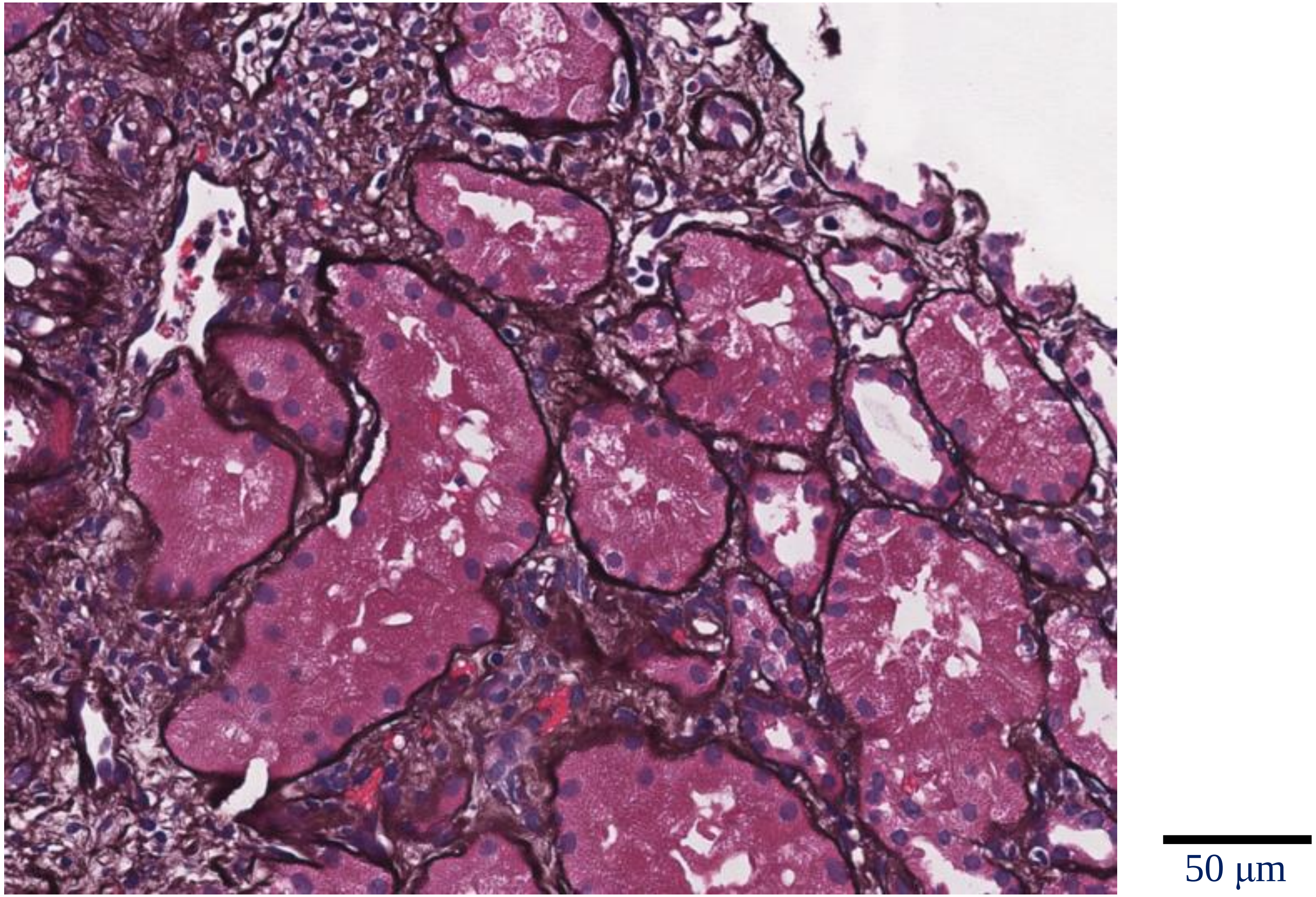

4 – Perfect
3 – Very good
2 – Good enough
1 – Not acceptable

	Stain quality score	Nuclear detail	Cytoplasmic detail	Basement membrane detail
Score				

Histochemically stained image #3

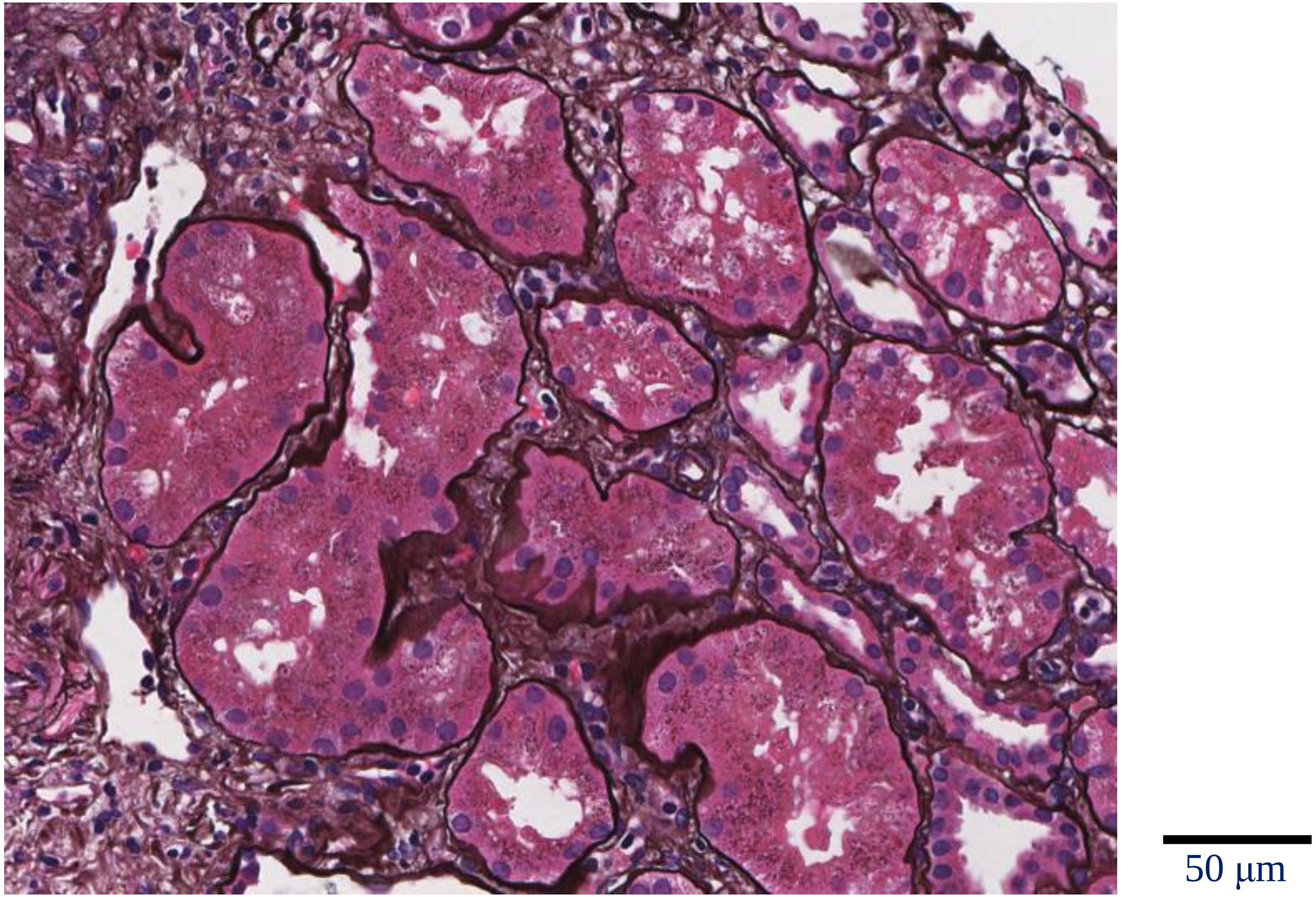

4 – Perfect

3 – Very good

2 – Good enough

1 – Not acceptable

	Stain quality score	Nuclear detail	Cytoplasmic detail	Basement membrane detail
Score				

Stain-transformed image #4

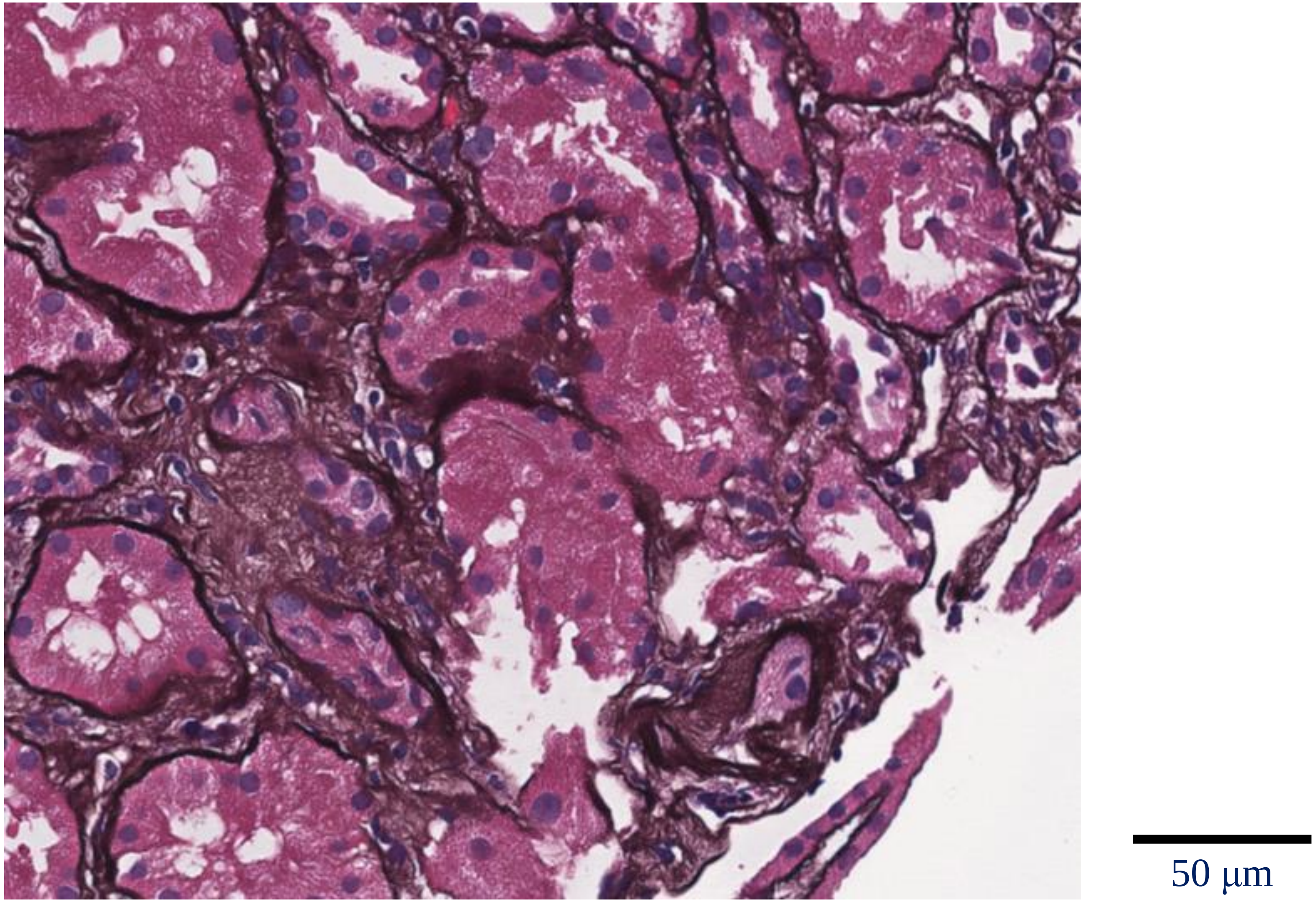

4 – Perfect
3 – Very good
2 – Good enough
1 – Not acceptable

	Stain quality score	Nuclear detail	Cytoplasmic detail	Basement membrane detail
Score				

Histochemically stained image #4

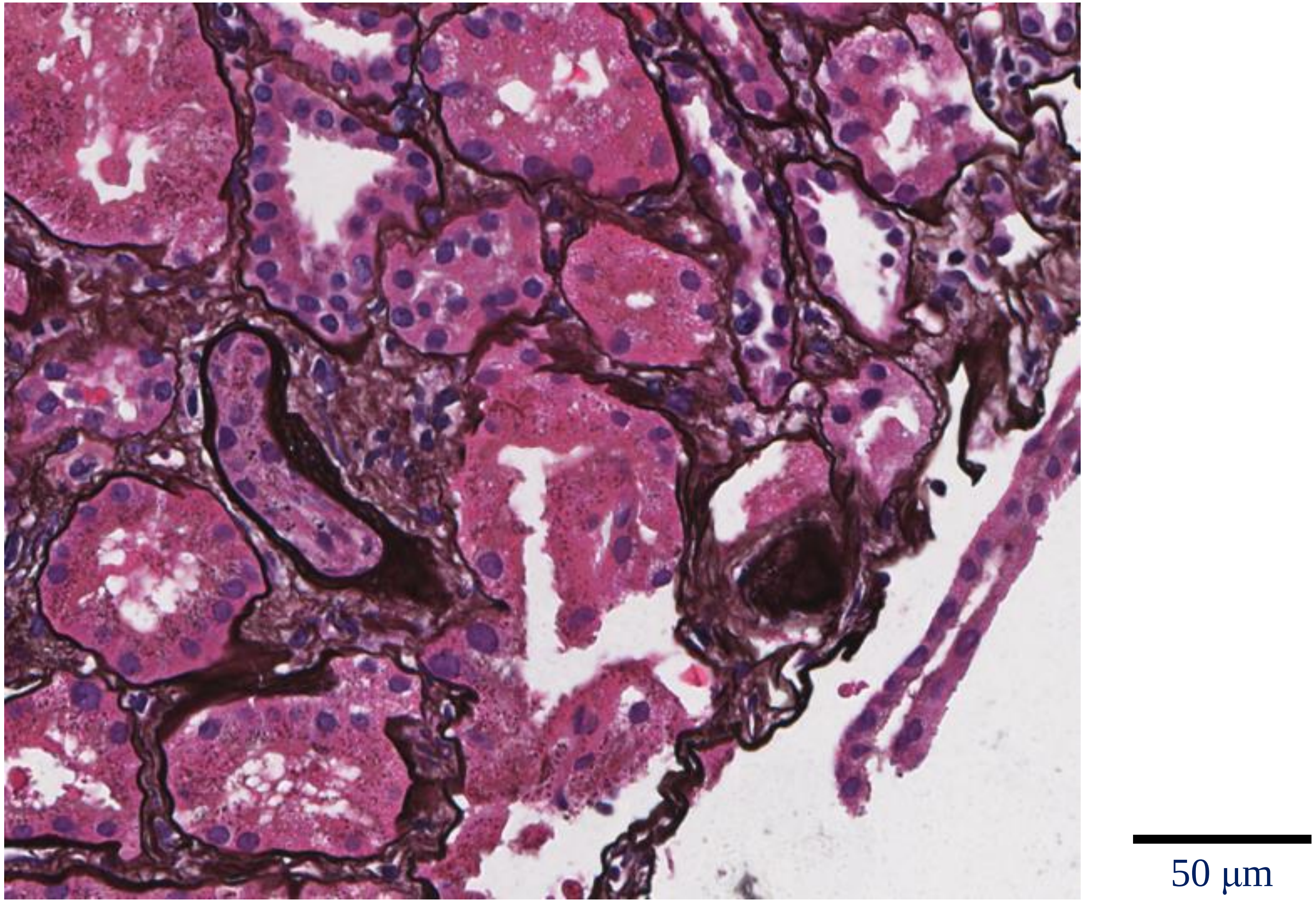

4 – Perfect
3 – Very good
2 – Good enough
1 – Not acceptable

	Stain quality score	Nuclear detail	Cytoplasmic detail	Basement membrane detail
Score				

Stain-transformed image #5

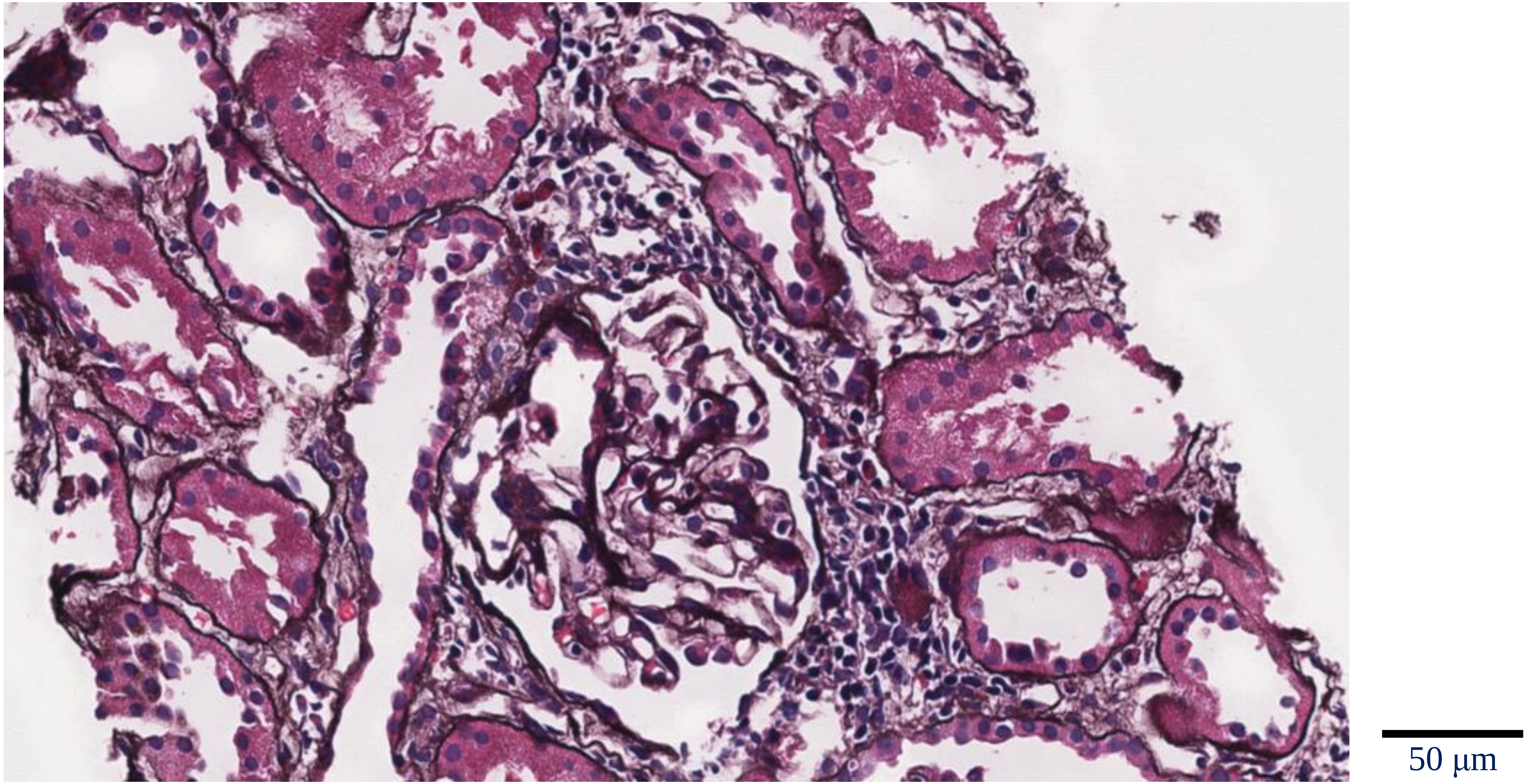

4 – Perfect

3 – Very good

2 – Good enough

1 – Not acceptable

	Stain quality score	Nuclear detail	Cytoplasmic detail	Basement membrane detail
Score				

Histochemically stained image #5

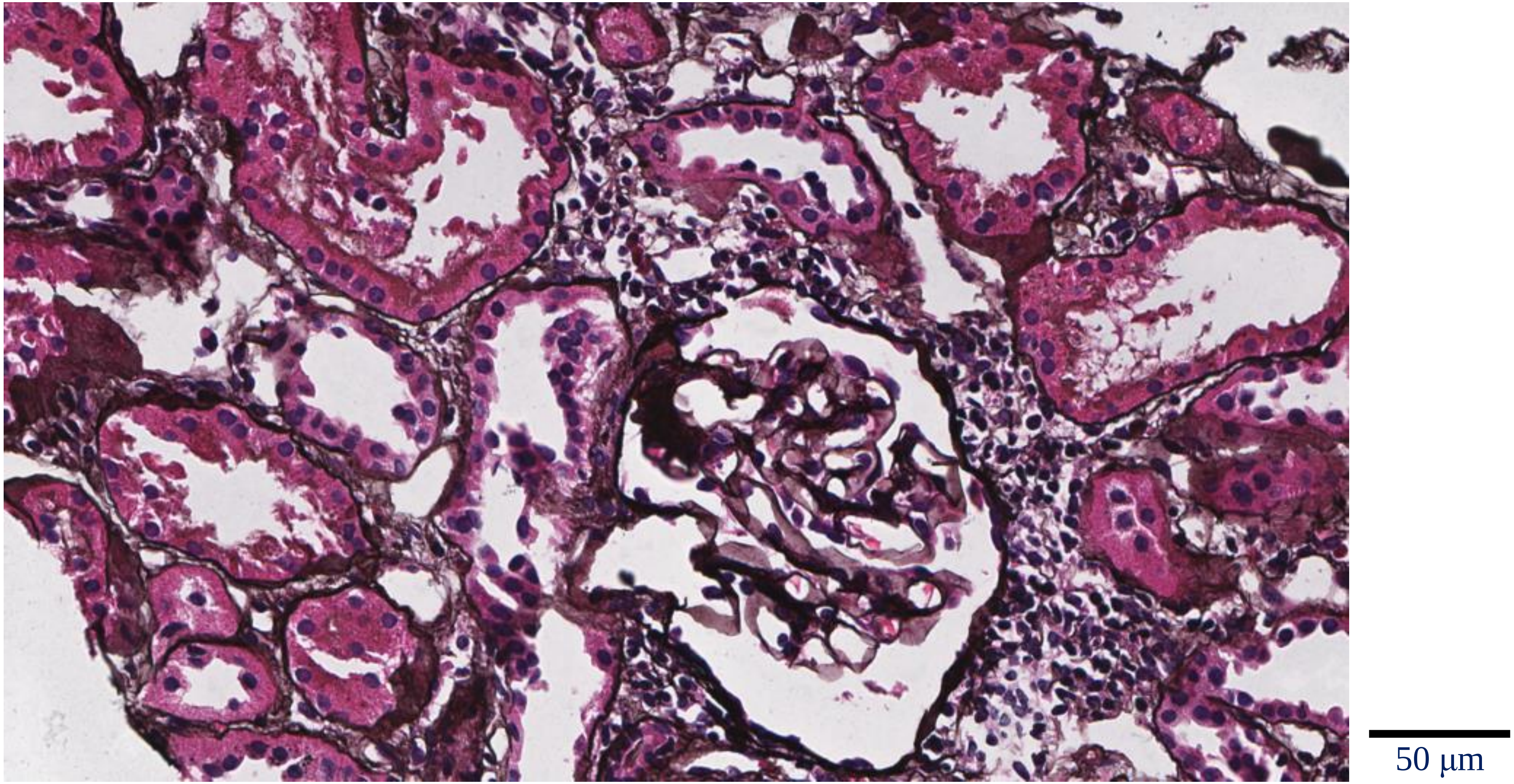

4 – Perfect
3 – Very good
2 – Good enough
1 – Not acceptable

	Stain quality score	Nuclear detail	Cytoplasmic detail	Basement membrane detail
Score				

Stain-transformed image #6

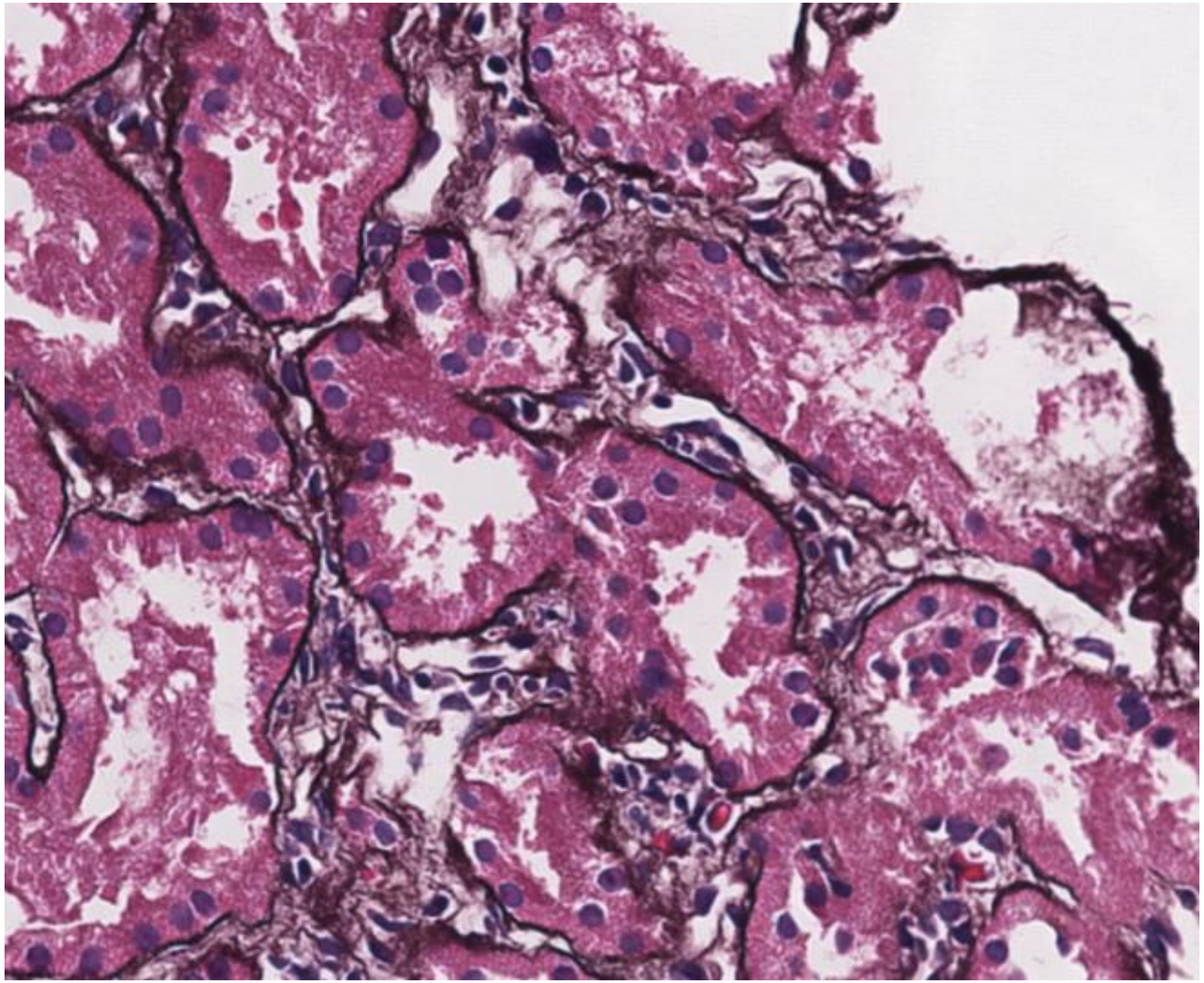

50 μm

4 – Perfect
3 – Very good
2 – Good enough
1 – Not acceptable

	Stain quality score	Nuclear detail	Cytoplasmic detail	Basement membrane detail
Score				

Histochemically stained image #6

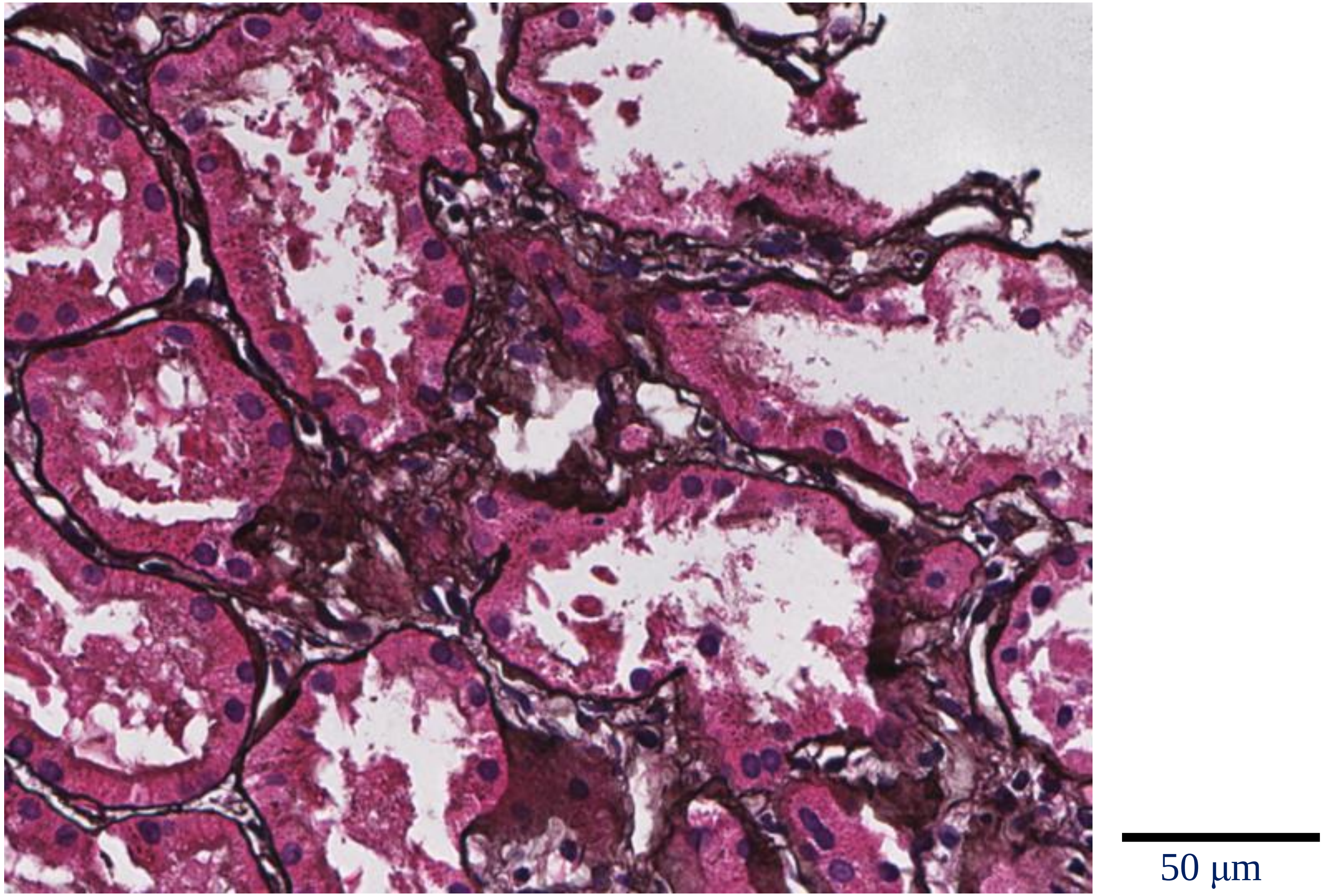

4 – Perfect
3 – Very good
2 – Good enough
1 – Not acceptable

	Stain quality score	Nuclear detail	Cytoplasmic detail	Basement membrane detail
**Score**				

Stain-transformed image #7

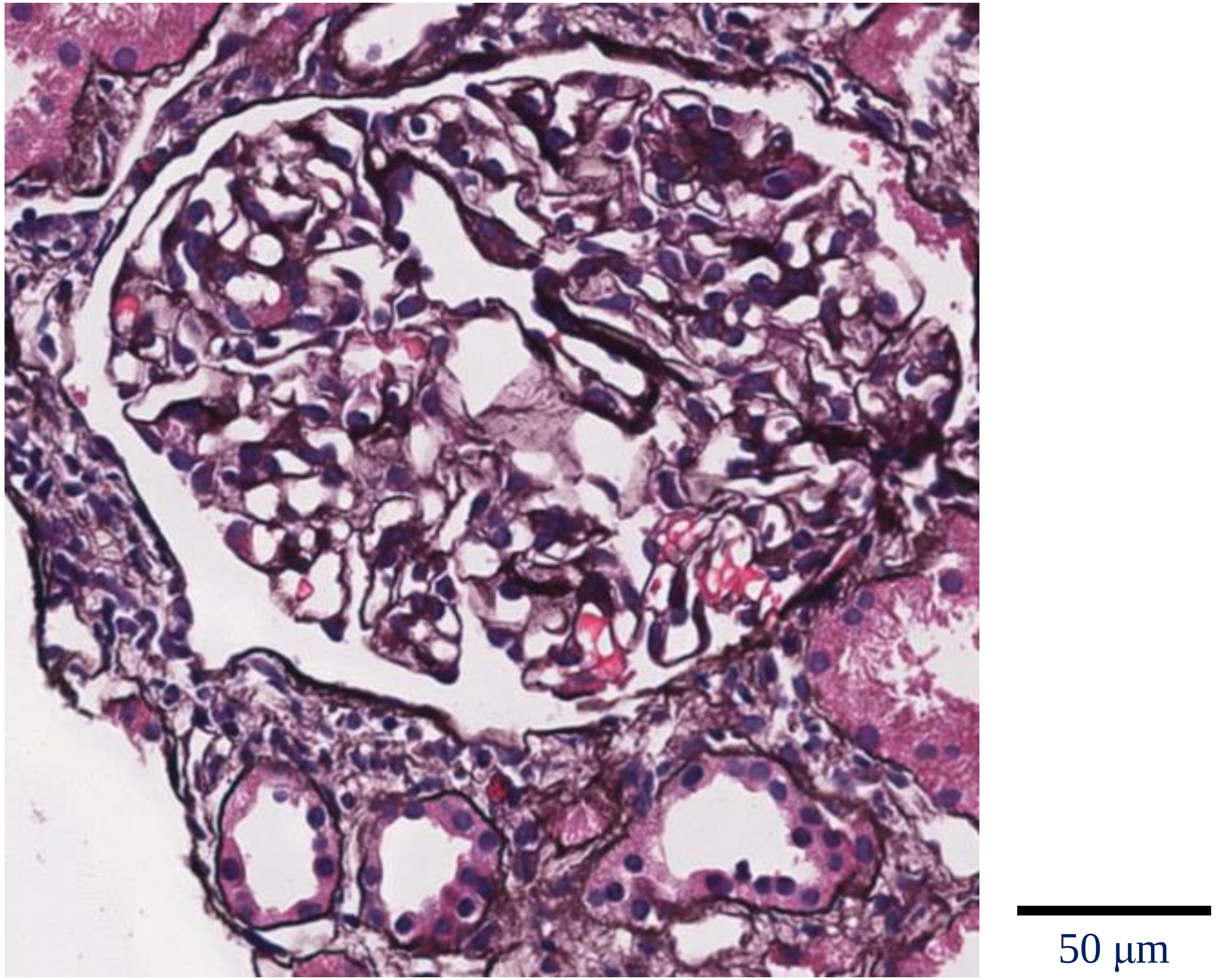

4 – Perfect
3 – Very good
2 – Good enough
1 – Not acceptable

	Stain quality score	Nuclear detail	Cytoplasmic detail	Basement membrane detail
Score				

Histochemically stained image #7

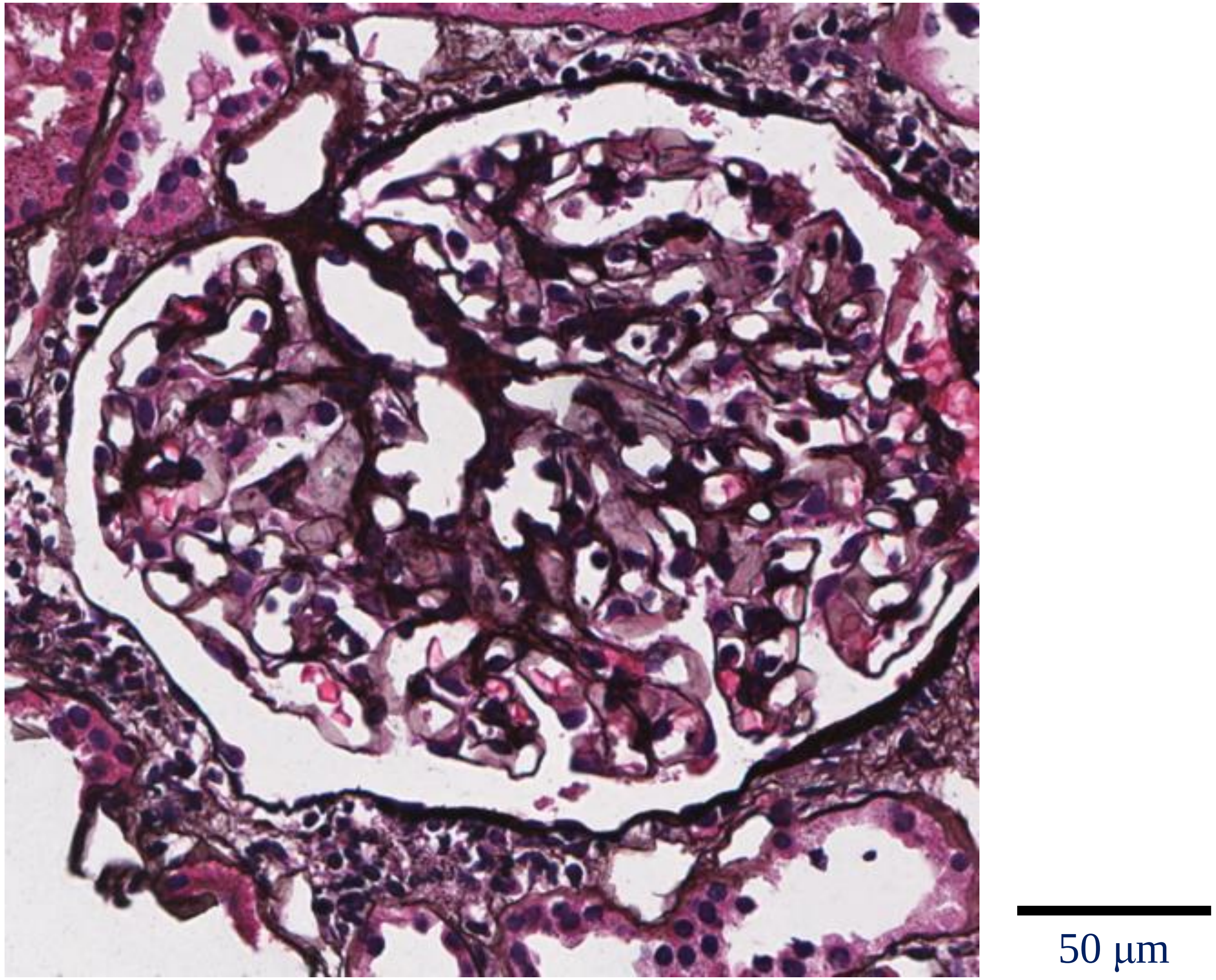

4 – Perfect
3 – Very good
2 – Good enough
1 – Not acceptable

	Stain quality score	Nuclear detail	Cytoplasmic detail	Basement membrane detail
Score				

Stain-transformed image #8

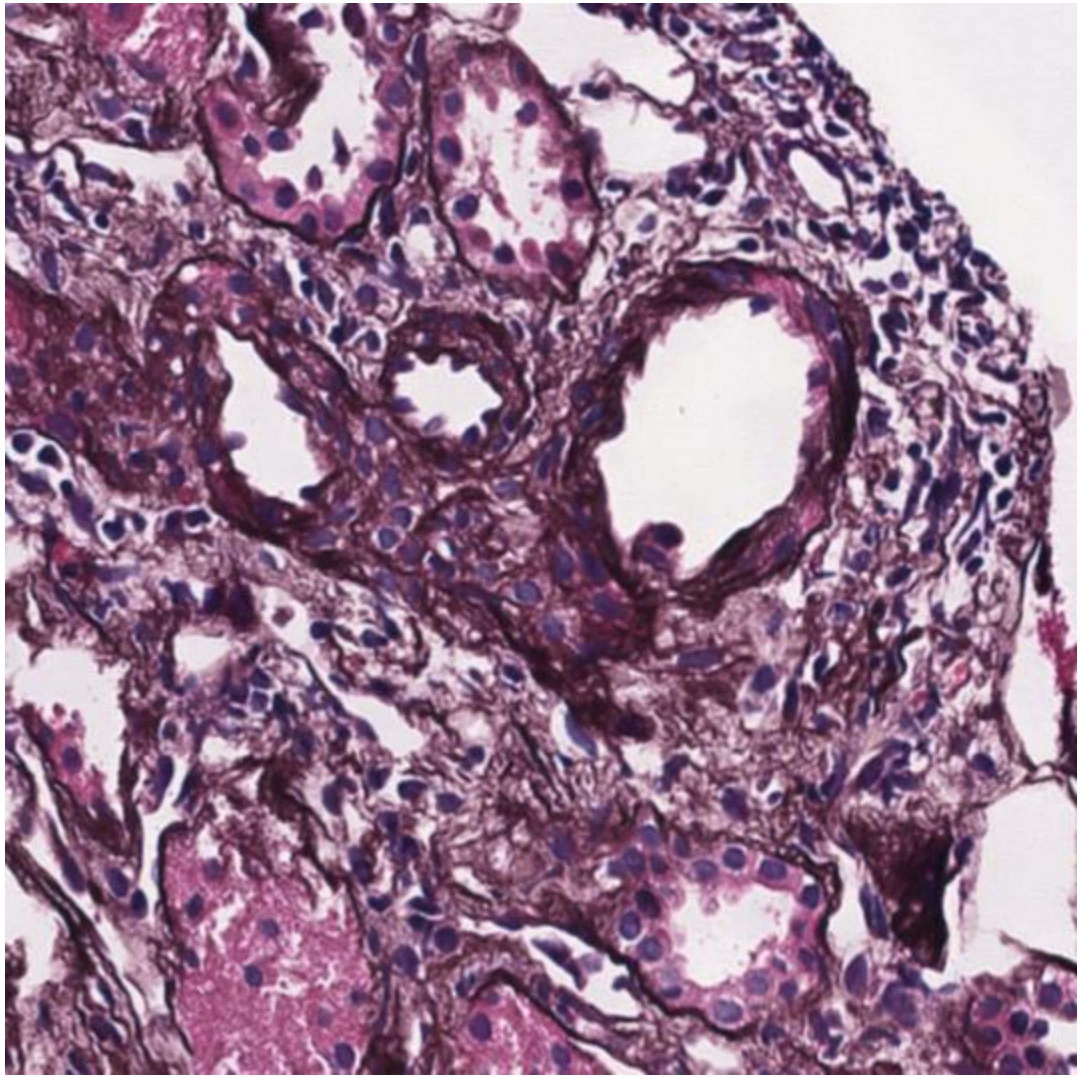

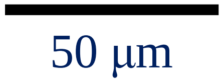

4 – Perfect

3 – Very good

2 – Good enough

1 – Not acceptable

	Stain quality score	Nuclear detail	Cytoplasmic detail	Basement membrane detail
Score				

Histochemically stained image #8

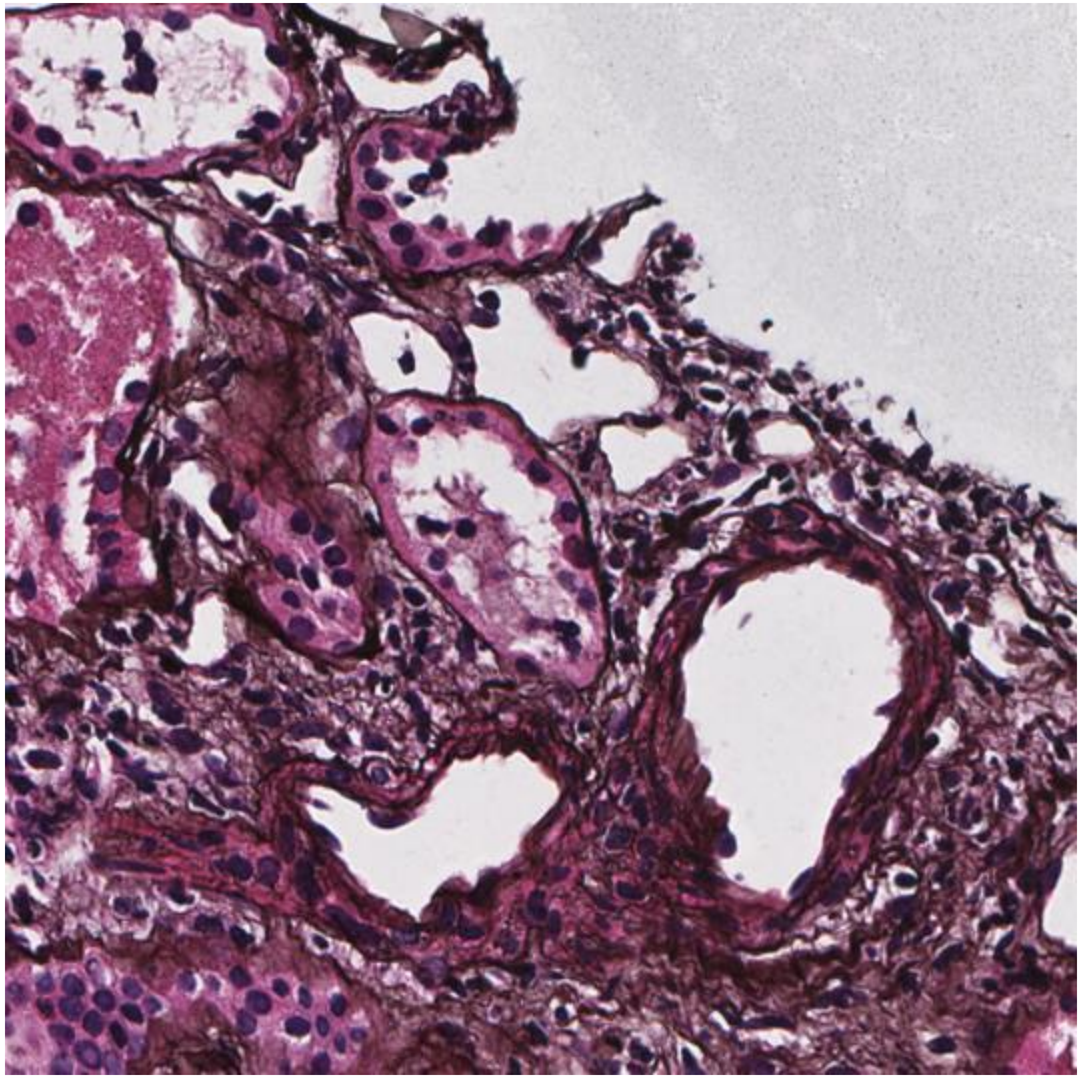

50 µm

4 – Perfect
3 – Very good
2 – Good enough
1 – Not acceptable

	Stain quality score	Nuclear detail	Cytoplasmic detail	Basement membrane detail
Score				

Stain-transformed image #9

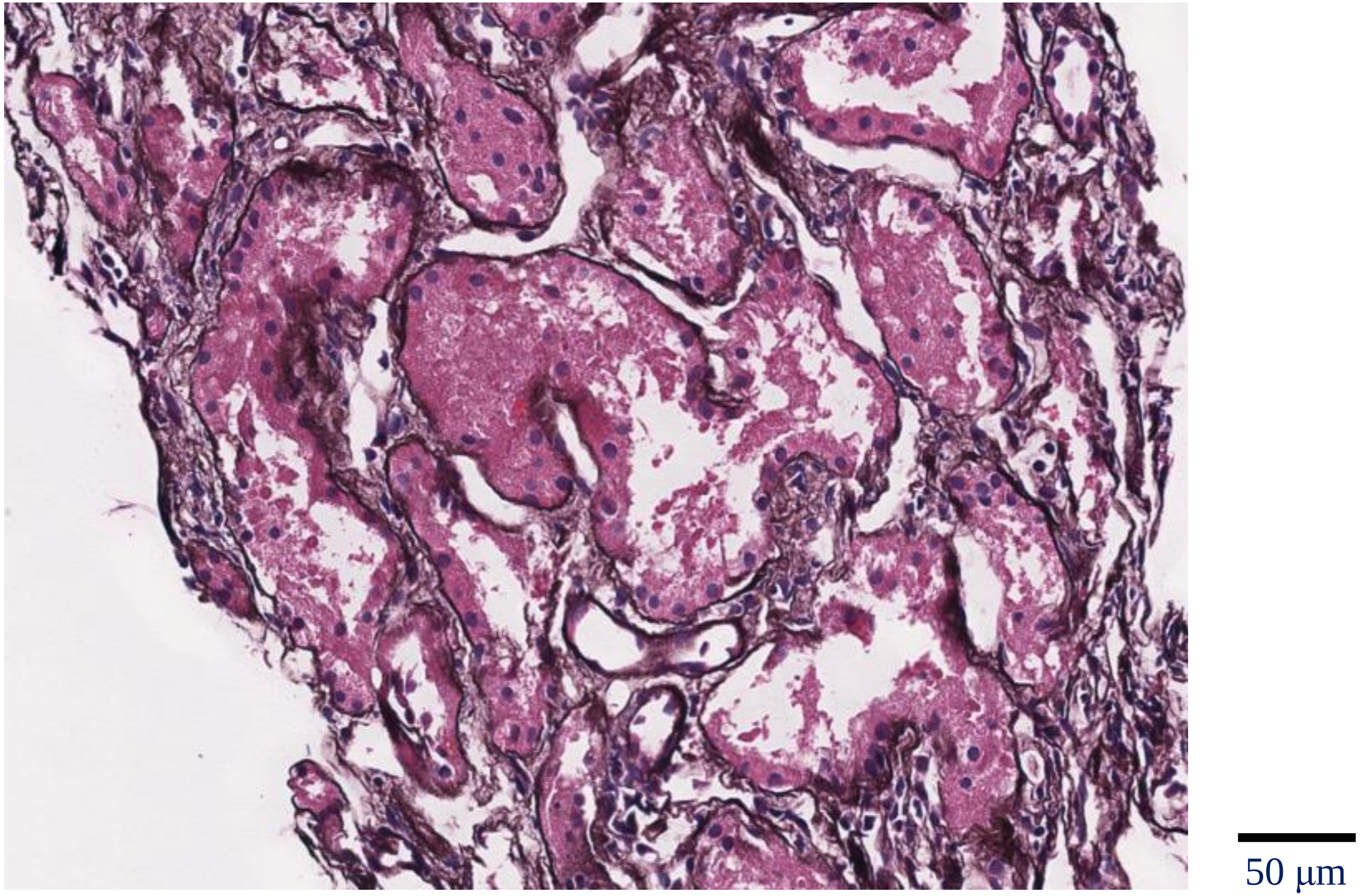

4 – Perfect
3 – Very good
2 – Good enough
1 – Not acceptable

	Stain quality score	Nuclear detail	Cytoplasmic detail	Basement membrane detail
Score				

Histochemically stained image #9

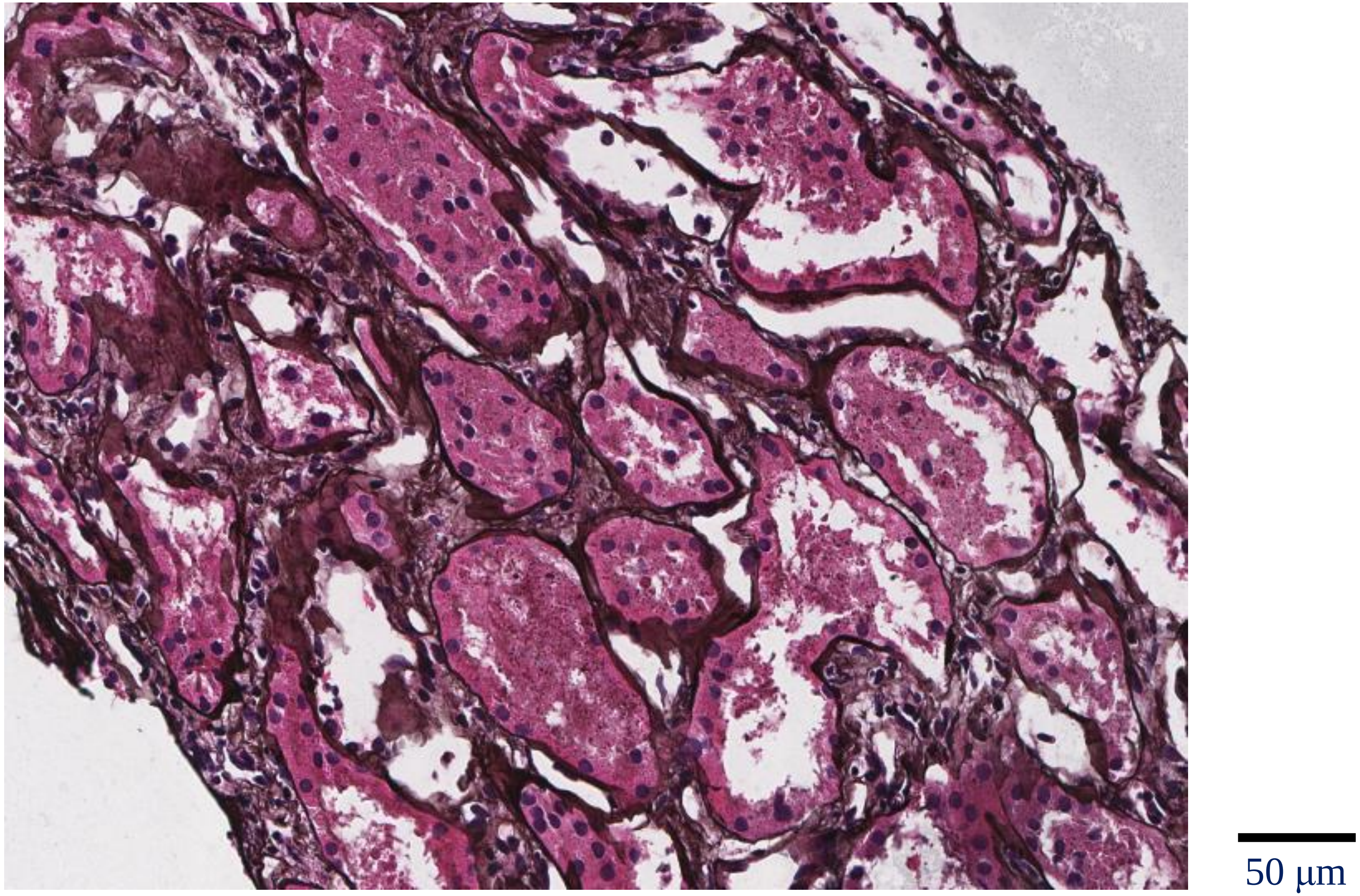

4 – Perfect
3 – Very good
2 – Good enough
1 – Not acceptable

	Stain quality score	Nuclear detail	Cytoplasmic detail	Basement membrane detail
Score				

Stain-transformed image #10

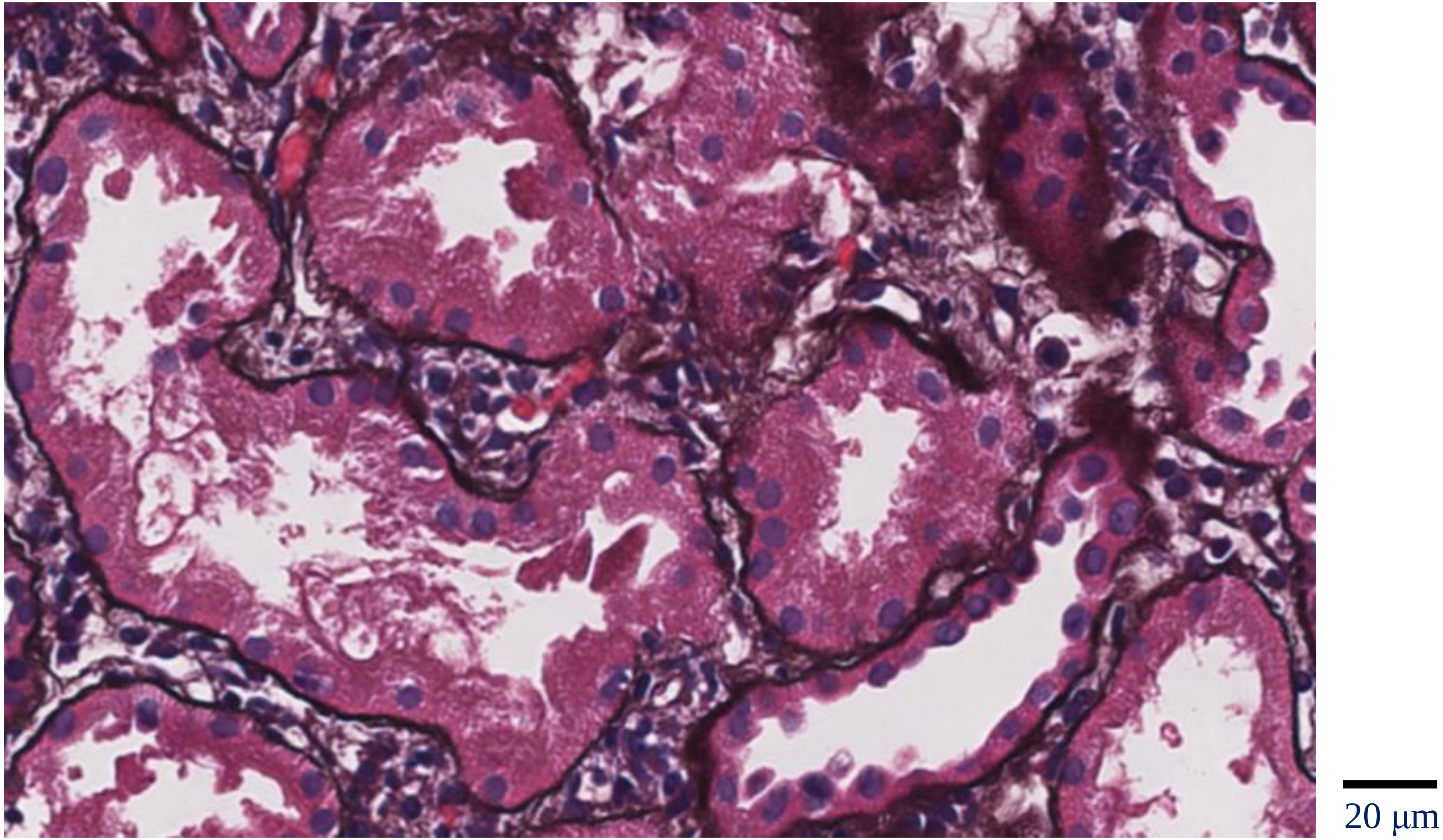

4 – Perfect
3 – Very good
2 – Good enough
1 – Not acceptable

	Stain quality score	Nuclear detail	Cytoplasmic detail	Basement membrane detail
Score				

Histochemically stained image #10

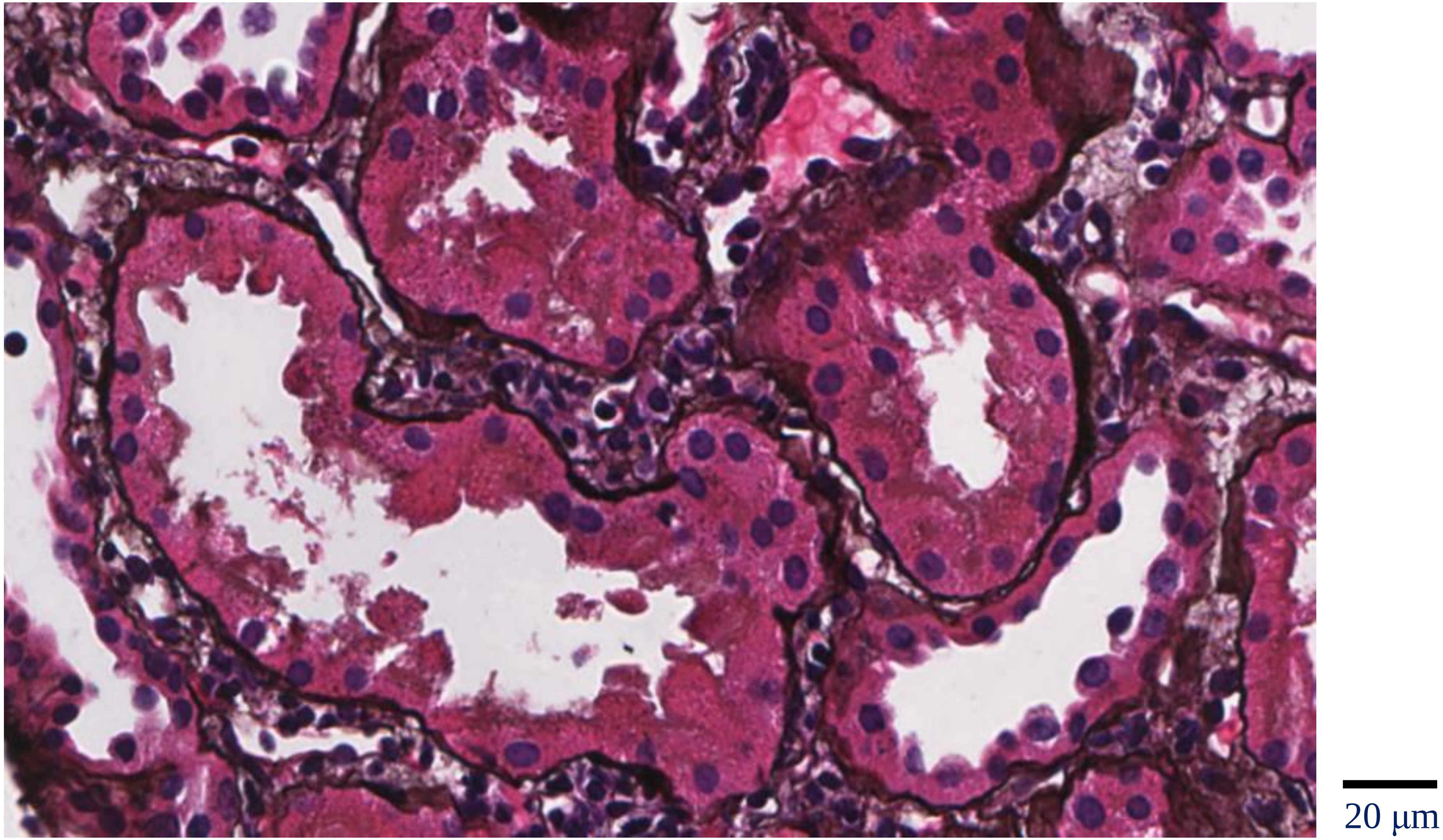

4 – Perfect
3 – Very good
2 – Good enough
1 – Not acceptable

	Stain quality score	Nuclear detail	Cytoplasmic detail	Basement membrane detail
Score				

Stain-transformed image #11

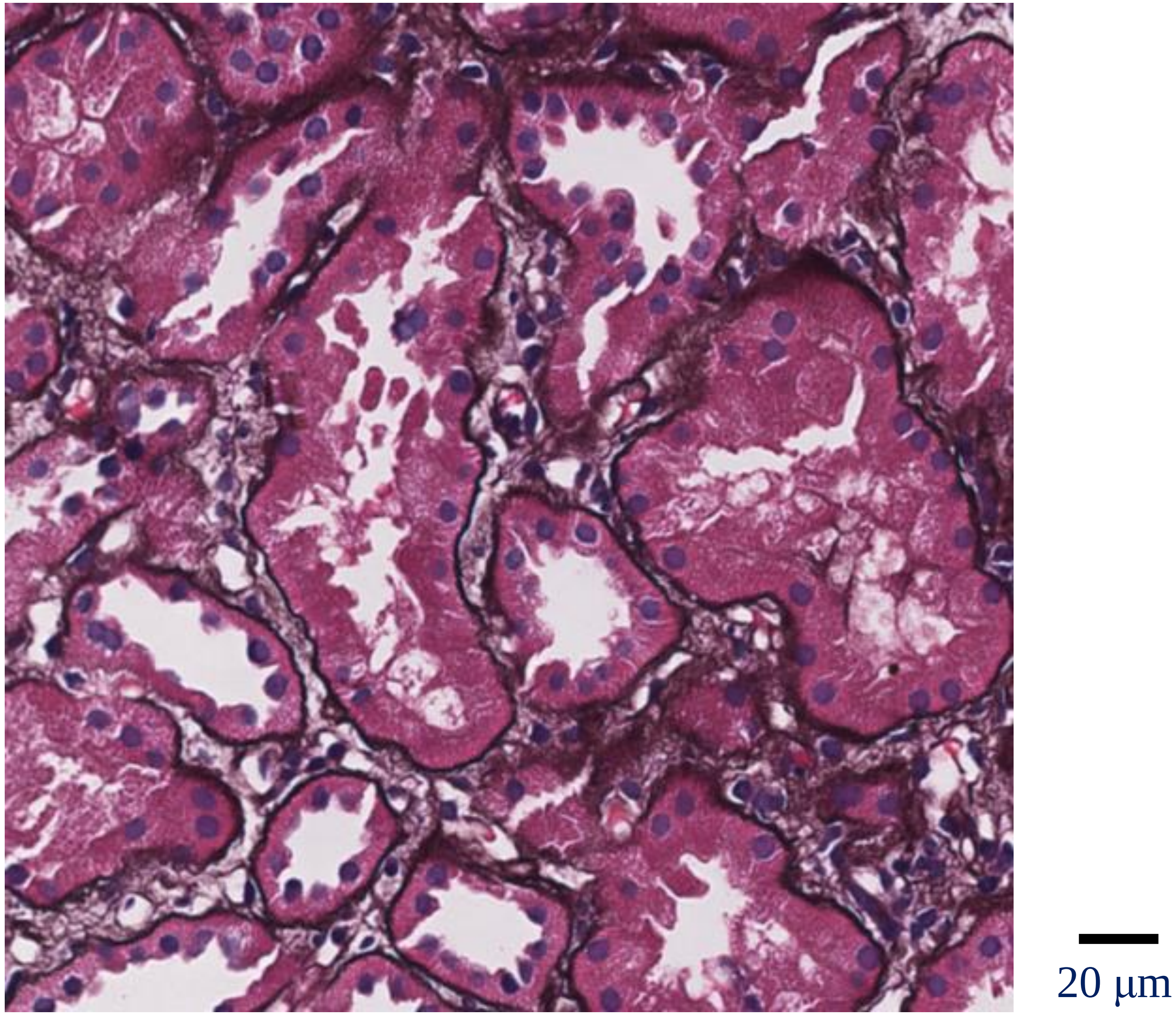

4 – Perfect
3 – Very good
2 – Good enough
1 – Not acceptable

	Stain quality score	Nuclear detail	Cytoplasmic detail	Basement membrane detail
Score				

Histochemically stained image #11

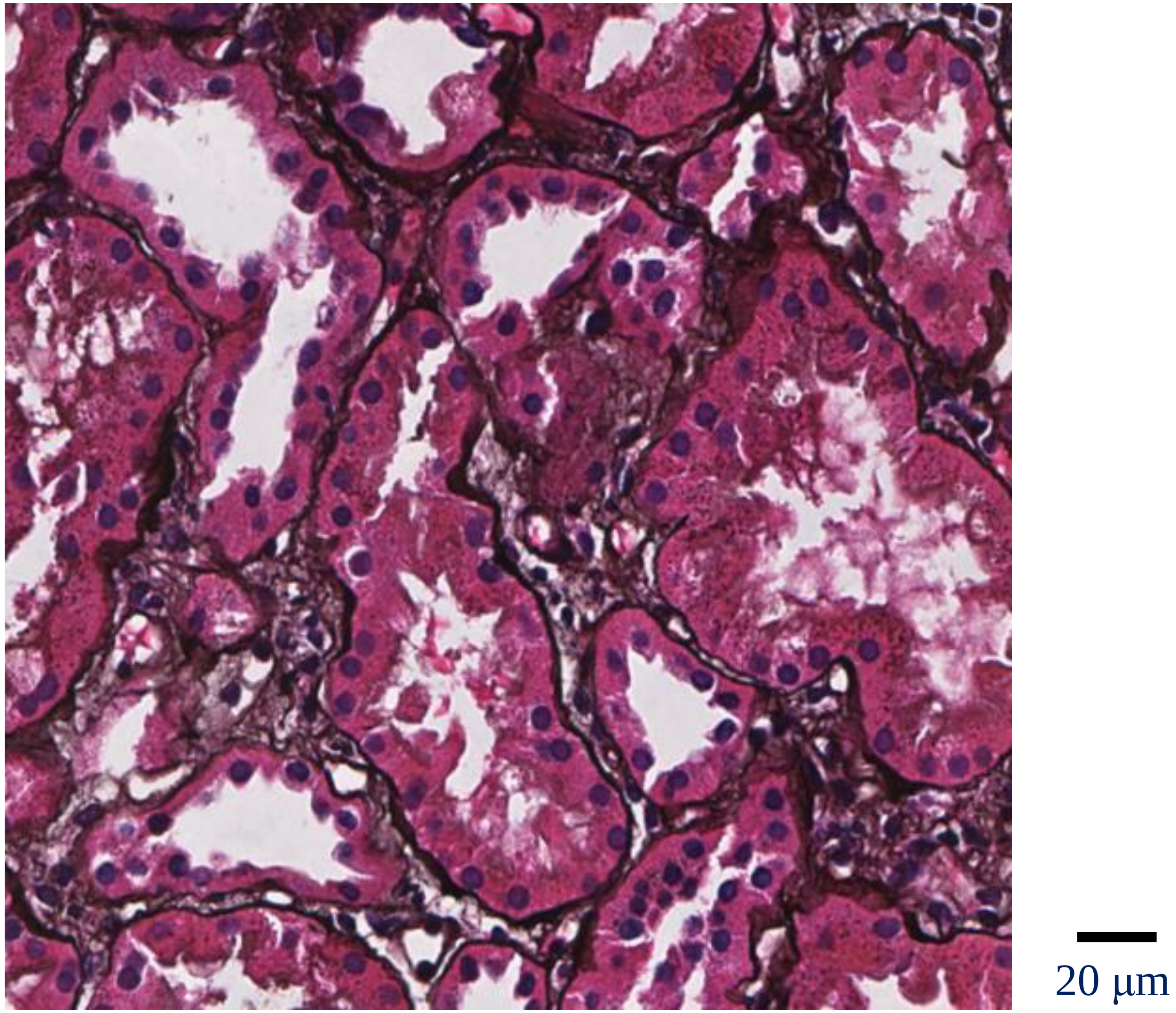

4 – Perfect
3 – Very good
2 – Good enough
1 – Not acceptable

	Stain quality score	Nuclear detail	Cytoplasmic detail	Basement membrane detail
Score				

Stain-transformed image #12

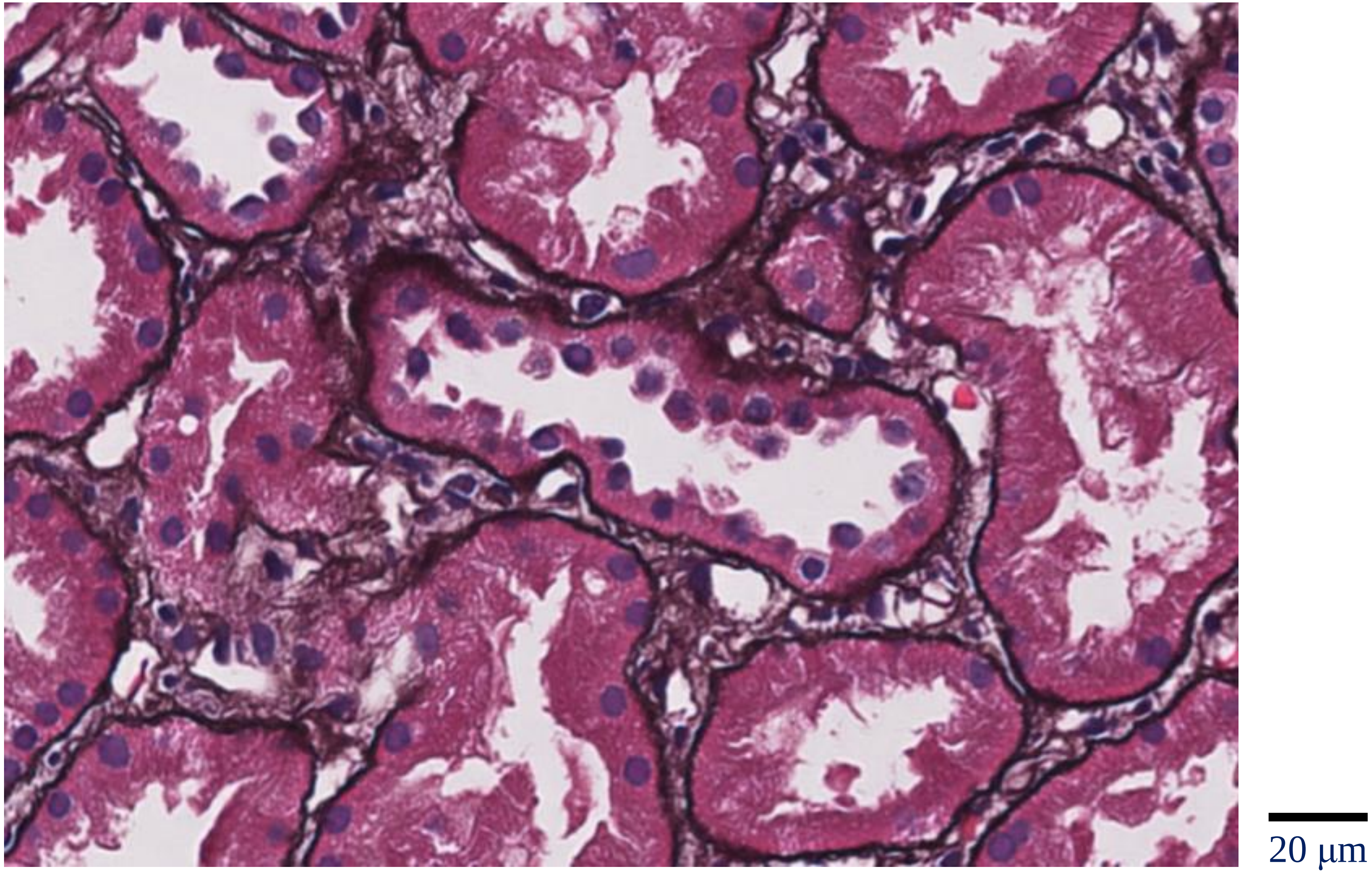

4 – Perfect
3 – Very good
2 – Good enough
1 – Not acceptable

	Stain quality score	Nuclear detail	Cytoplasmic detail	Basement membrane detail
Score				

Histochemically stained image #12

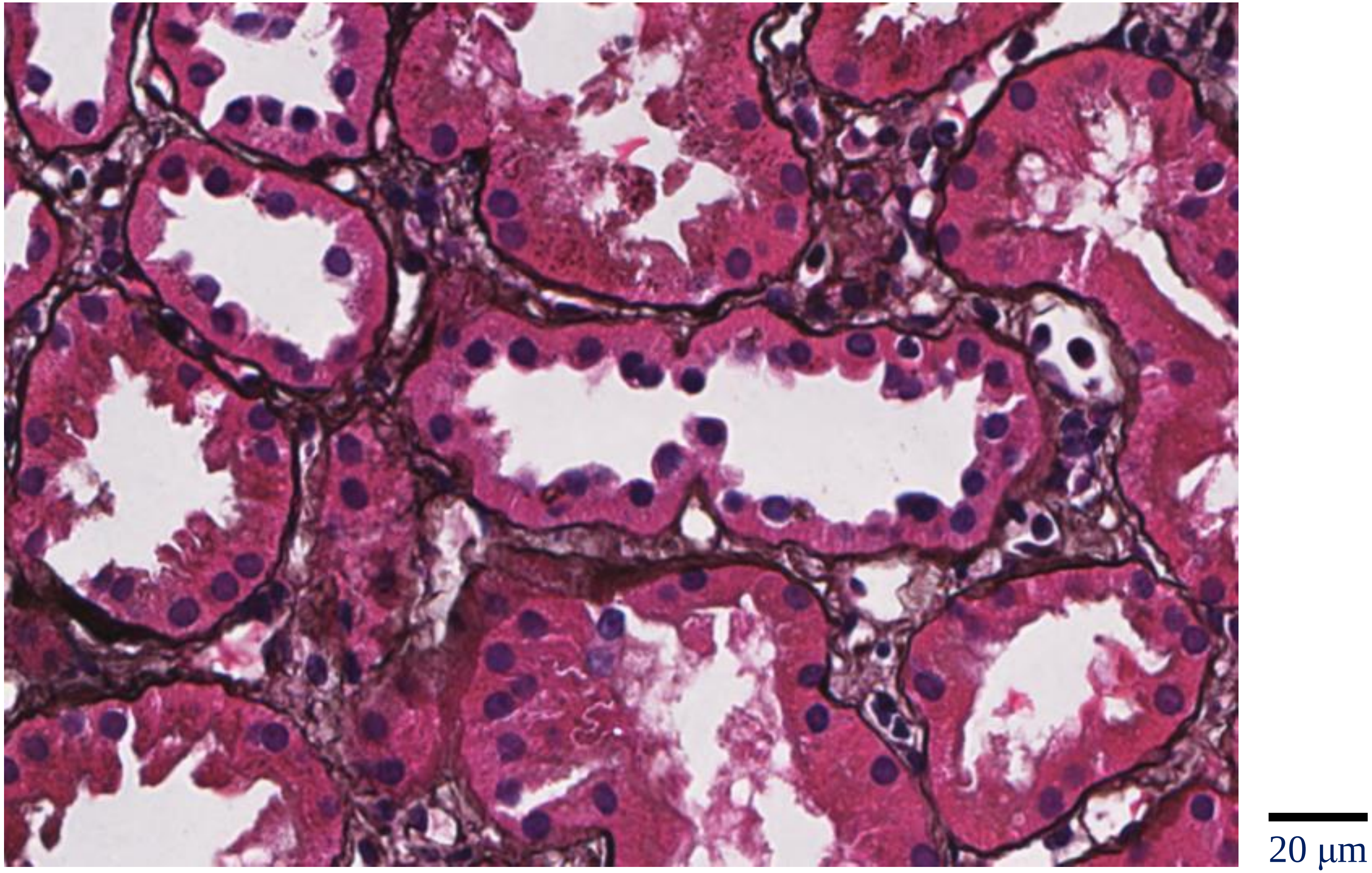

4 – Perfect
3 – Very good
2 – Good enough
1 – Not acceptable

	Stain quality score	Nuclear detail	Cytoplasmic detail	Basement membrane detail
Score				

Stain-transformed image #13

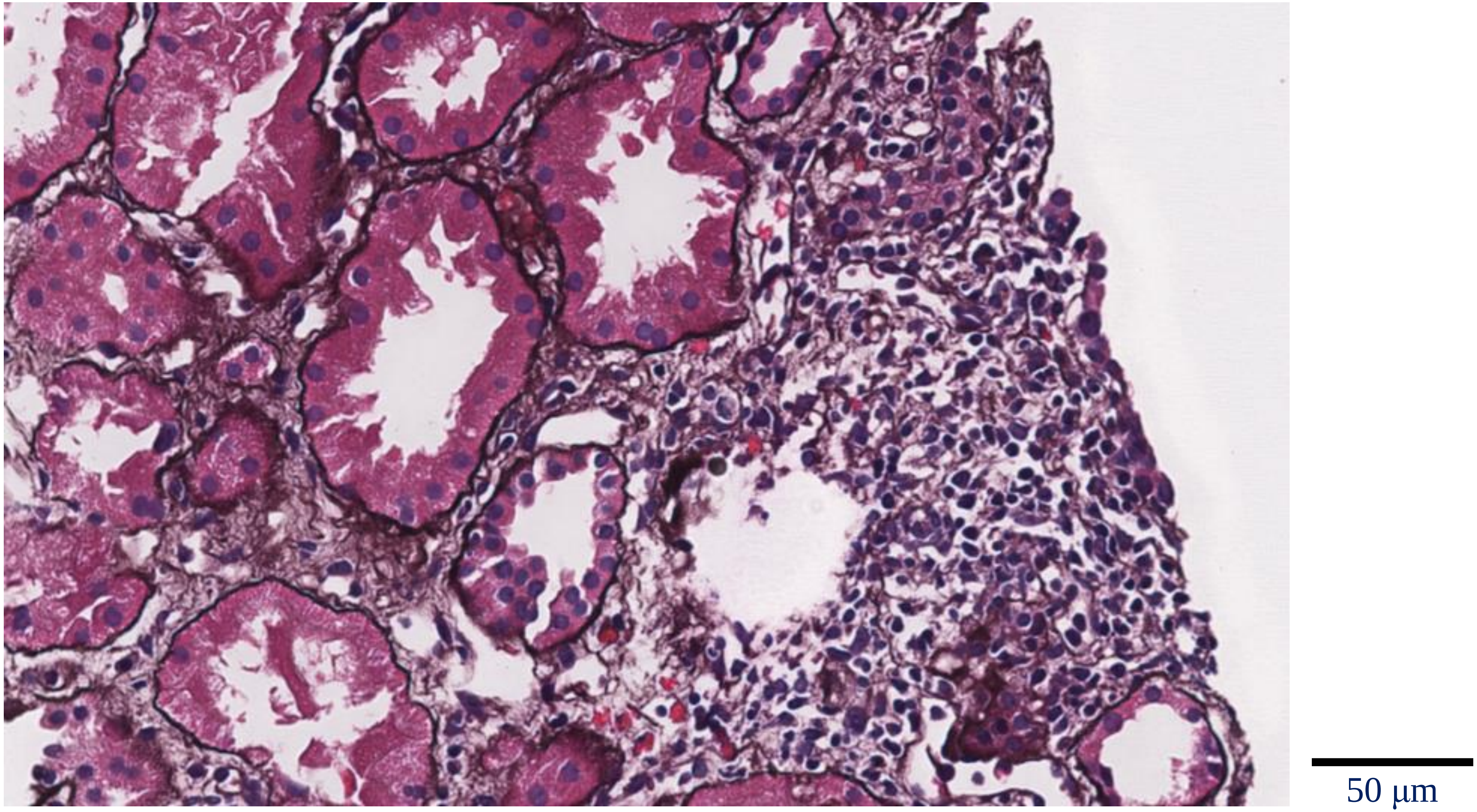

4 – Perfect
3 – Very good
2 – Good enough
1 – Not acceptable

	Stain quality score	Nuclear detail	Cytoplasmic detail	Basement membrane detail
Score				

Histochemically stained image #13

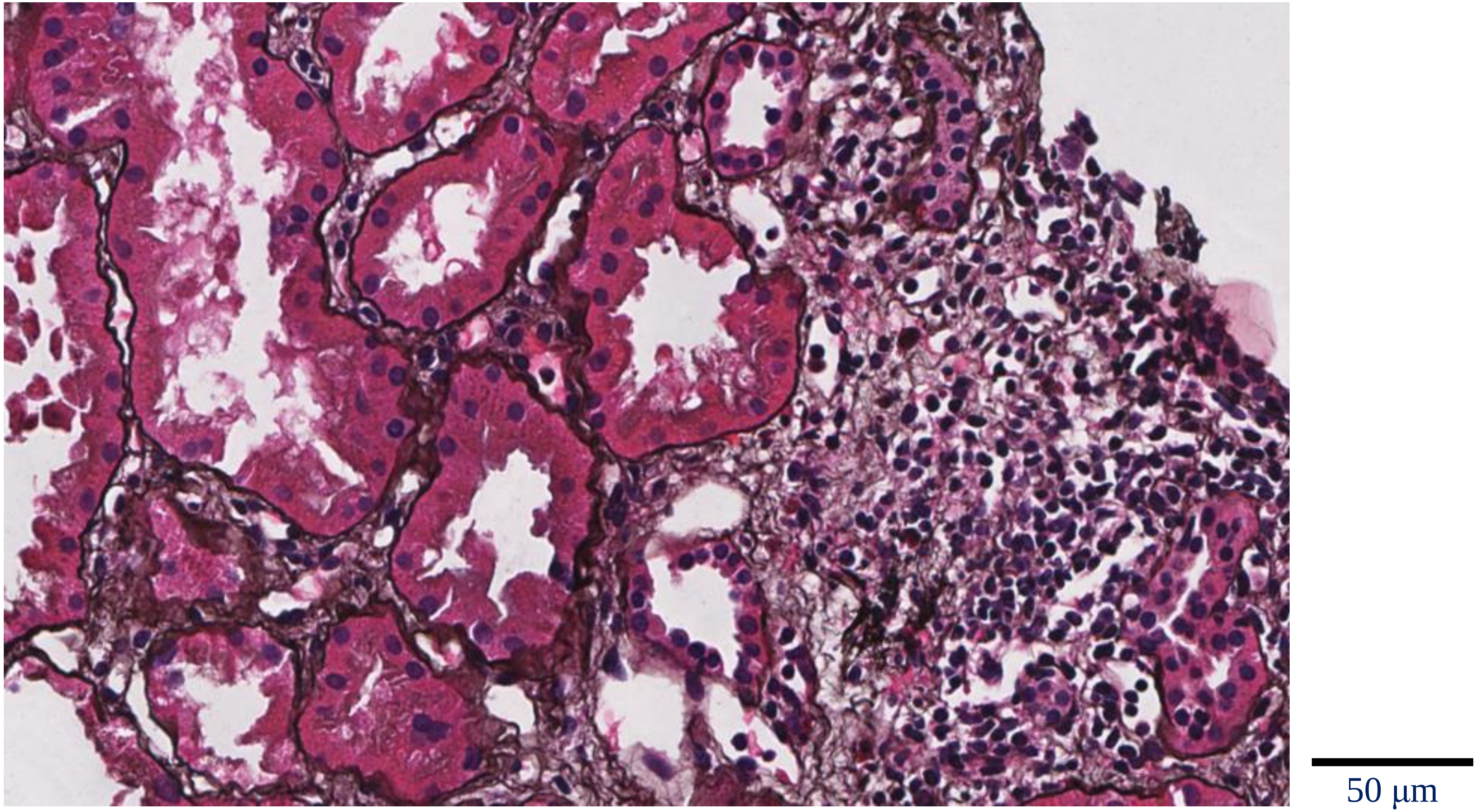

4 – Perfect
3 – Very good
2 – Good enough
1 – Not acceptable

	Stain quality score	Nuclear detail	Cytoplasmic detail	Basement membrane detail
Score				

Stain-transformed image #14

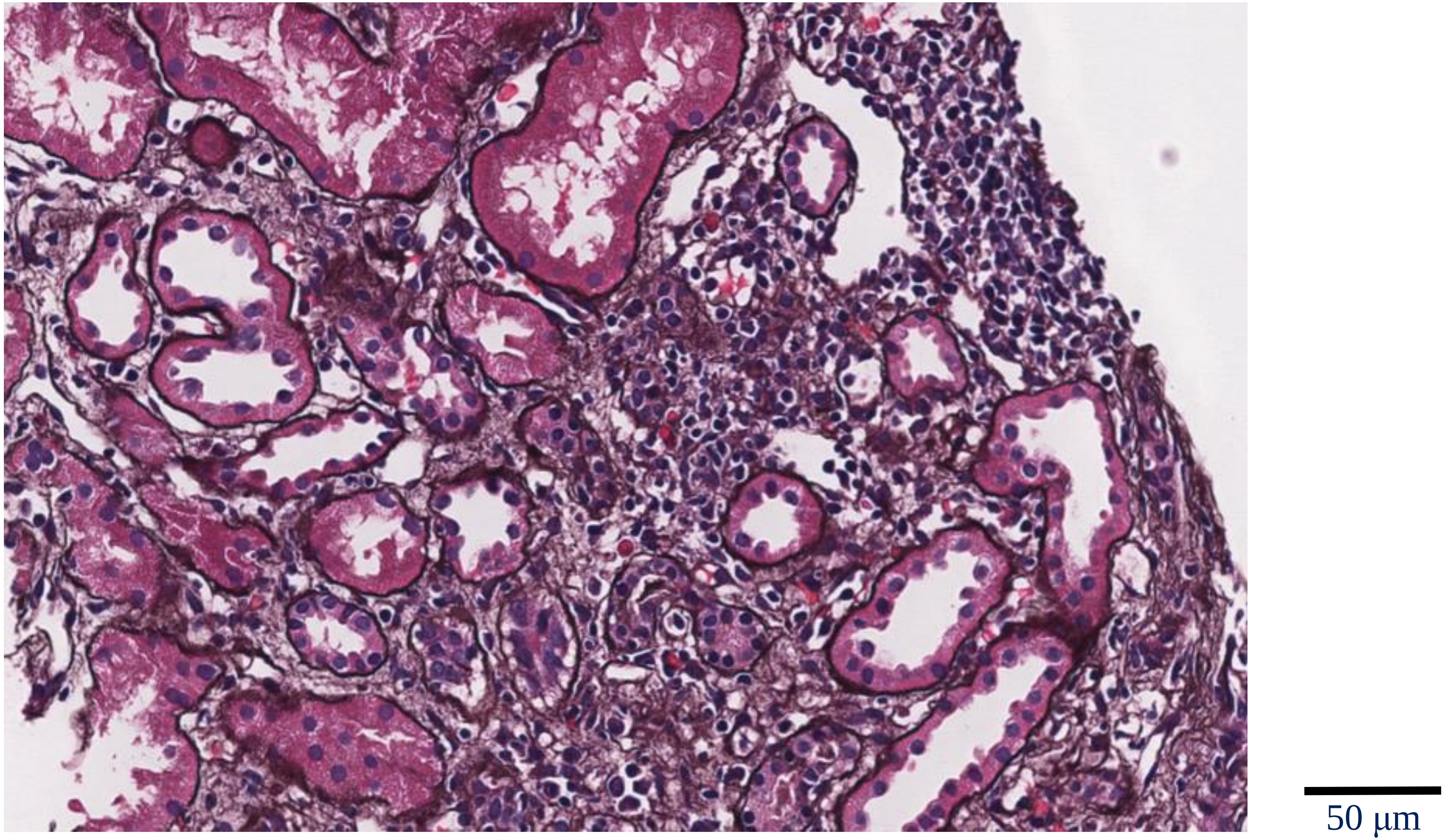

4 – Perfect
3 – Very good
2 – Good enough
1 – Not acceptable

	Stain quality score	Nuclear detail	Cytoplasmic detail	Basement membrane detail
Score				

Histochemically stained image #14

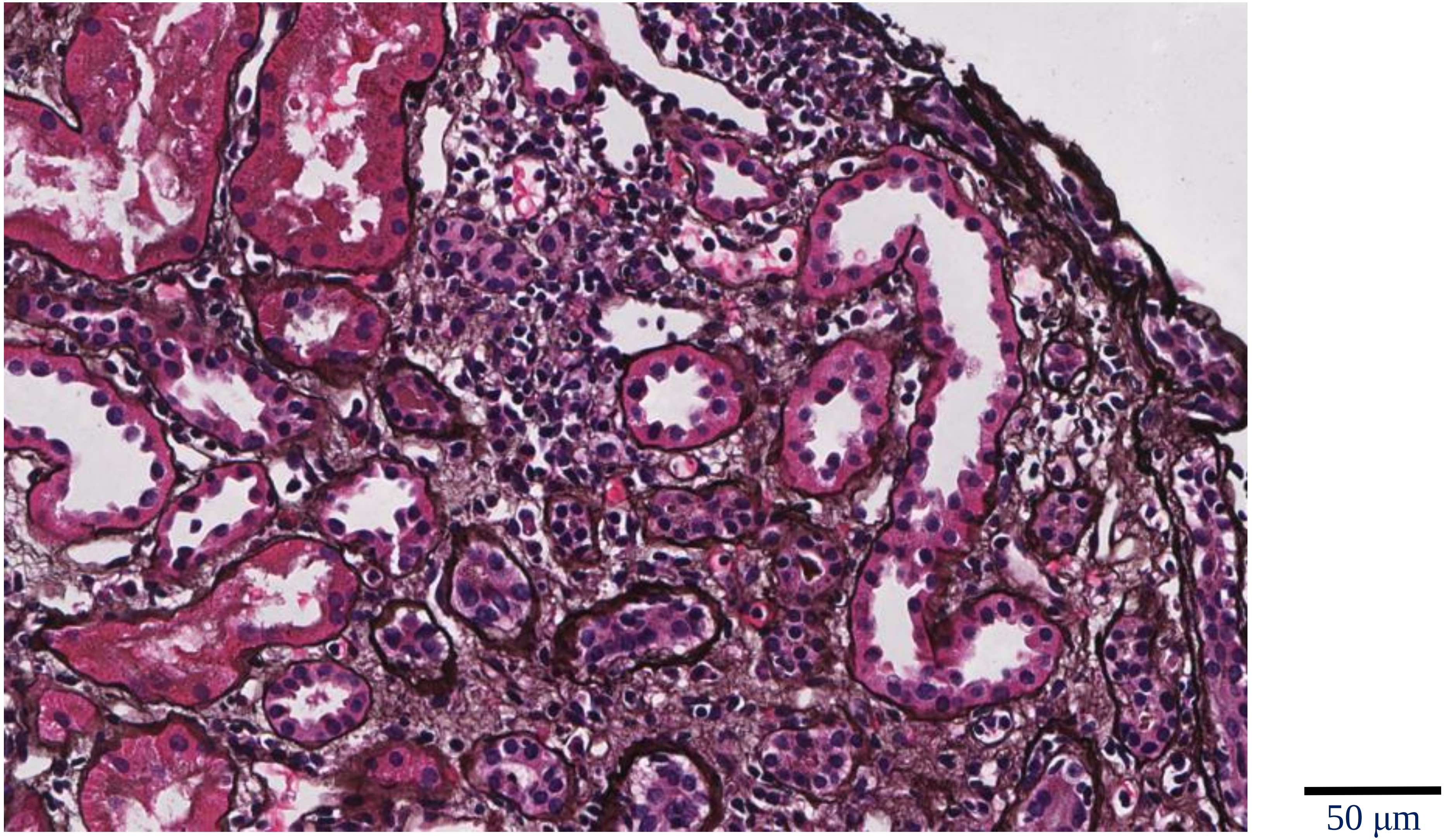

4 – Perfect
3 – Very good
2 – Good enough
1 – Not acceptable

	Stain quality score	Nuclear detail	Cytoplasmic detail	Basement membrane detail
Score				

Stain-transformed image #15

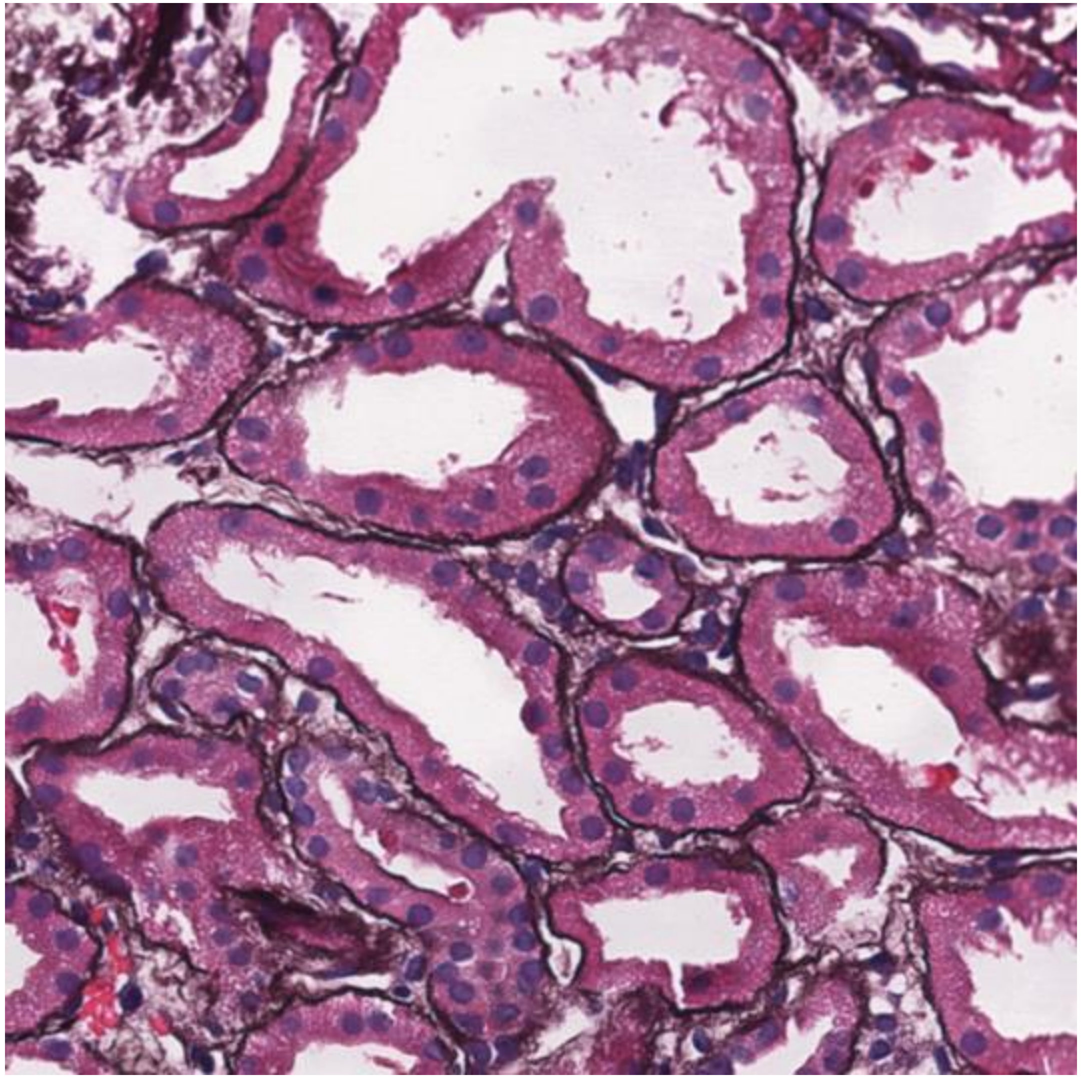

50 µm

4 – Perfect
3 – Very good
2 – Good enough
1 – Not acceptable

	Stain quality score	Nuclear detail	Cytoplasmic detail	Basement membrane detail
Score				

Histochemically stained image #15

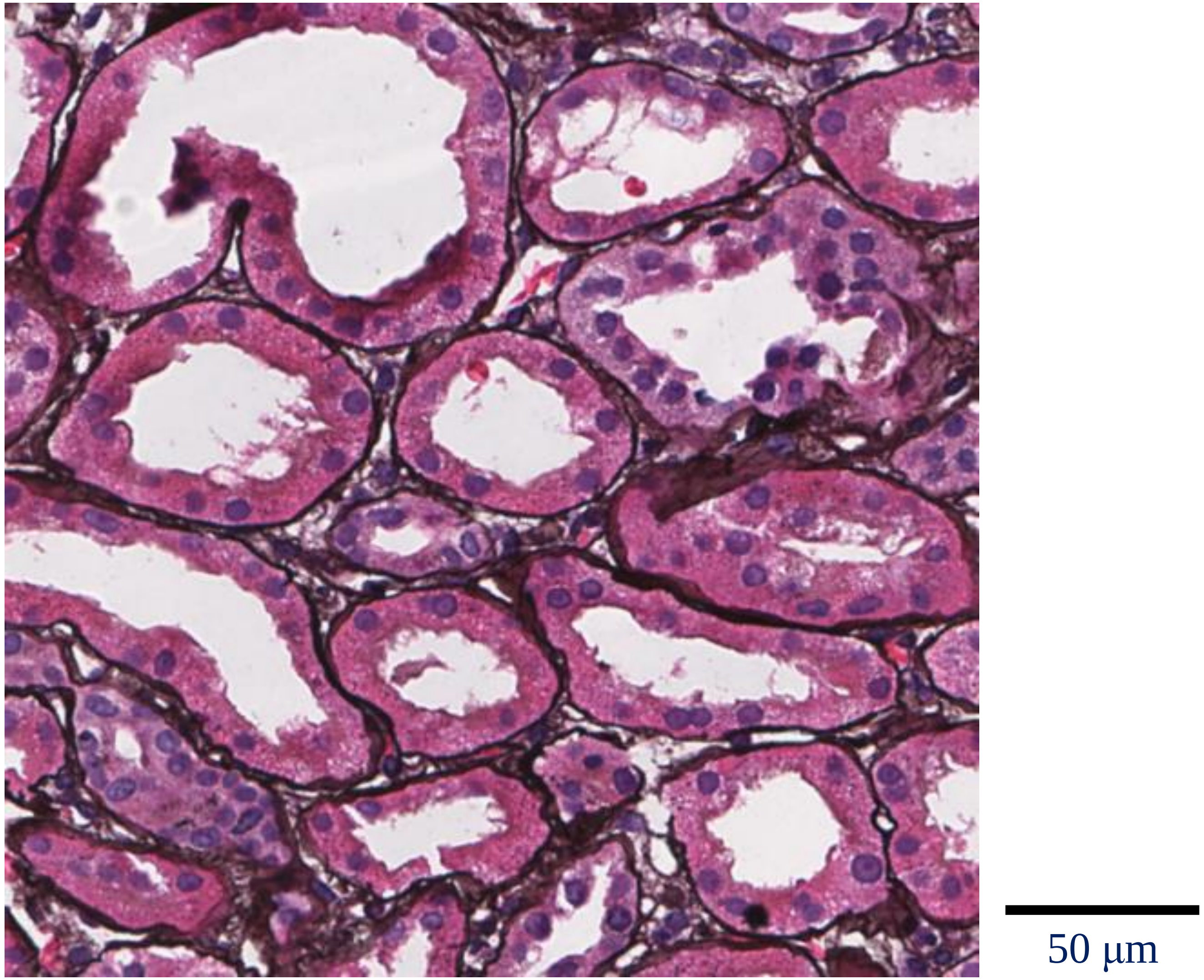

4 – Perfect
3 – Very good
2 – Good enough
1 – Not acceptable

	Stain quality score	Nuclear detail	Cytoplasmic detail	Basement membrane detail
Score				

Stain-transformed image #16

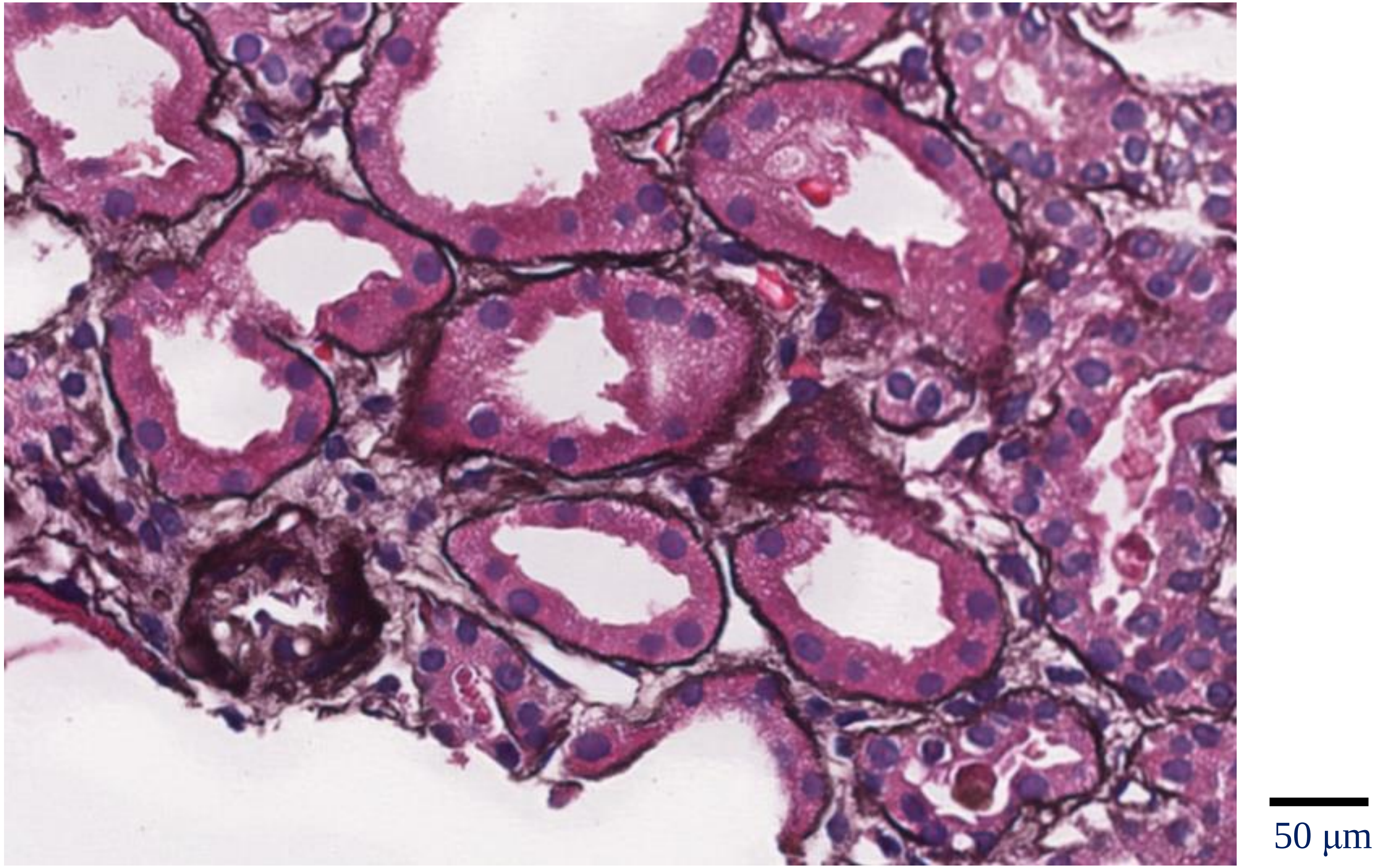

4 – Perfect
3 – Very good
2 – Good enough
1 – Not acceptable

	Stain quality score	Nuclear detail	Cytoplasmic detail	Basement membrane detail
Score				

Histochemically stained image #16

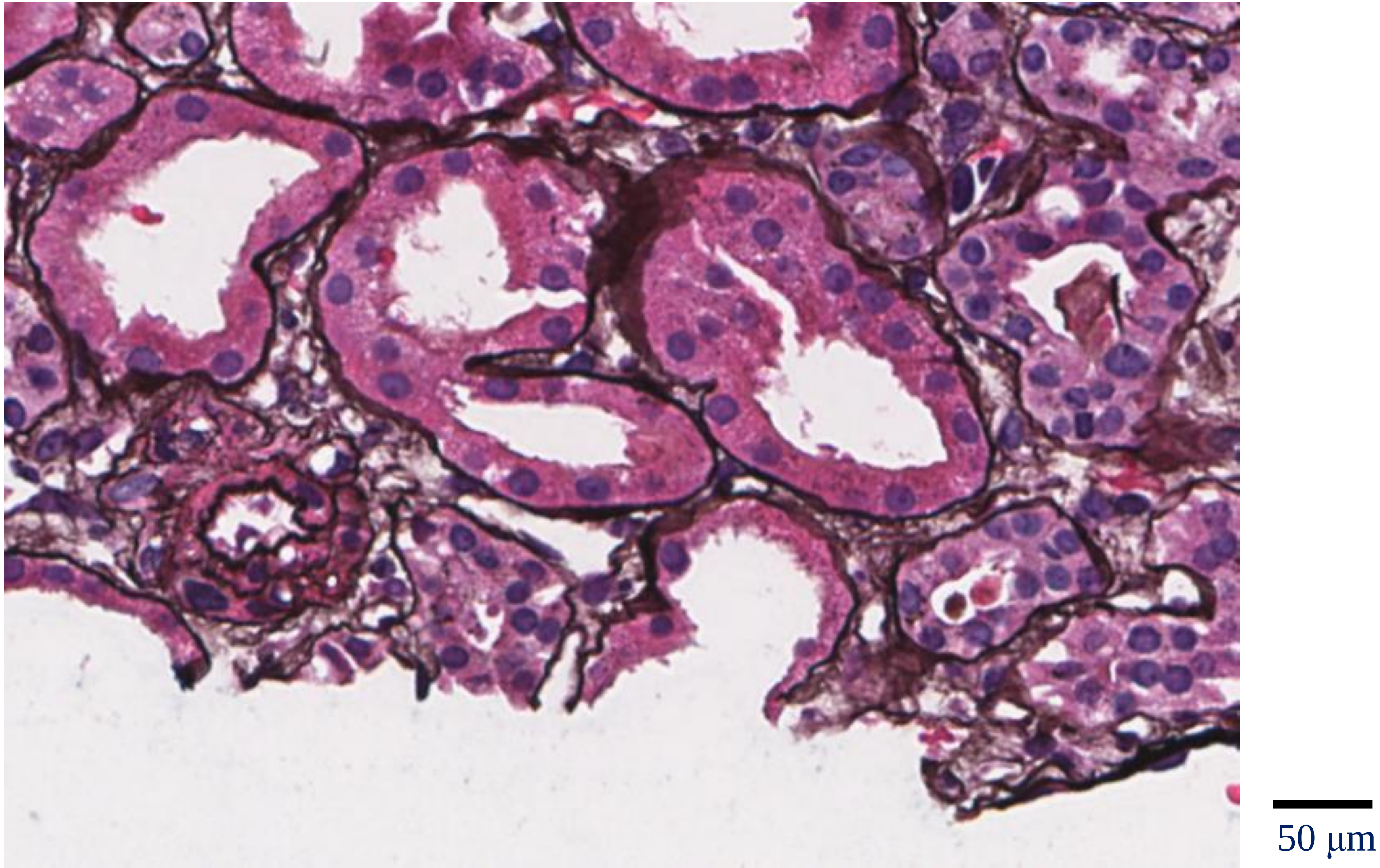

4 – Perfect
3 – Very good
2 – Good enough
1 – Not acceptable

	Stain quality score	Nuclear detail	Cytoplasmic detail	Basement membrane detail
Score				
