# Supplementary material for: Deep learning-based transformation of H&E stained tissues into special stains
Source: Nat Commun. 2021 Aug 12;12:4884. doi: 10.1038/s41467-021-25221-2 (PMC8361203; doi:10.1038/s41467-021-25221-2)
Supplement: Supplementary file 6 — Reporting Summary [file 41467_2021_25221_MOESM6_ESM.pdf]

nature research

Corresponding author(s): Yair Rivenson, Dean Wallace, Aydogan Ozcan

Last updated by author(s): Jul 16, 2021

Reporting Summary

Nature Research wishes to improve the reproducibility of the work that we publish. This form provides structure for consistency and transparency in reporting. For further information on Nature Research policies, see our Editorial Policies and the Editorial Policy Checklist.

Statistics

For all statistical analyses, confirm that the following items are present in the figure legend, table legend, main text, or Methods section.

n/a	Confirmed	
☐	☒	The exact sample size (n) for each experimental group/condition, given as a discrete number and unit of measurement
☐	☒	A statement on whether measurements were taken from distinct samples or whether the same sample was measured repeatedly
☐	☒	The statistical test(s) used AND whether they are one- or two-sided<br>*Only common tests should be described solely by name; describe more complex techniques in the Methods section.*
☒	☐	A description of all covariates tested
☒	☐	A description of any assumptions or corrections, such as tests of normality and adjustment for multiple comparisons
☐	☒	A full description of the statistical parameters including central tendency (e.g. means) or other basic estimates (e.g. regression coefficient) AND variation (e.g. standard deviation) or associated estimates of uncertainty (e.g. confidence intervals)
☐	☒	For null hypothesis testing, the test statistic (e.g. F, t, r) with confidence intervals, effect sizes, degrees of freedom and P value noted<br>*Give P values as exact values whenever suitable.*
☒	☐	For Bayesian analysis, information on the choice of priors and Markov chain Monte Carlo settings
☒	☐	For hierarchical and complex designs, identification of the appropriate level for tests and full reporting of outcomes
☒	☐	Estimates of effect sizes (e.g. Cohen's d, Pearson's r), indicating how they were calculated

Our web collection on statistics for biologists contains articles on many of the points above.

Software and code

Policy information about availability of computer code

Data collection	Autofluorescence images of label-free tissue were captured using a fluorescence microscope (IX83, Olympus Corporation, Tokyo, Japan) which uses a motorized stage for scanning. The microscope was controlled by MetaMorph microscope automation software (version 7.10.161 Molecular Devices, LLC). Brightfield microscope images were taken from a pre-existing database. The image viewer was run using the Orthanc server package (version 1.8).
Data analysis	The neural networks were trained and implemented using Python version 3.6.2 with TensorFlow version 1.8.0. Image co-registration was performed using Matlab vR2018a (The MathWorks Inc.).

For manuscripts utilizing custom algorithms or software that are central to the research but not yet described in published literature, software must be made available to editors and reviewers. We strongly encourage code deposition in a community repository (e.g. GitHub). See the Nature Research guidelines for submitting code & software for further information.

Data

Policy information about availability of data

All manuscripts must include a data availability statement. This statement should provide the following information, where applicable:

- Accession codes, unique identifiers, or web links for publicly available datasets
- A list of figures that have associated raw data
- A description of any restrictions on data availability

Data supporting the results demonstrated by this study are available within the main text and the Supplementary Information. The full set of images used for the stain quality assessment study can be found in the Supplementary Images file as well as at: https://github.com/kevindehaan/stain-transformation. The full pathologist reports and adjudication results can be found in the supplementary data and on GitHub. Examples of patient sample fields of view can also be found on GitHub at https://github.com/kevindehaan/stain-transformation. For each example, the histochemically stained H&E and stain transformed special stains are shown

for the same field of view, while the histochemically stained special stains that are shown come from the same biopsy, through serial tissue sections. Raw whole slide images corresponding to patient specimen were obtained under UCLA IRB 18-001029 from the UCLA Health private database for the current study and therefore cannot be made publicly available.

Field-specific reporting

Please select the one below that is the best fit for your research. If you are not sure, read the appropriate sections before making your selection.

☒ Life sciences ☐ Behavioural & social sciences ☐ Ecological, evolutionary & environmental sciences

For a reference copy of the document with all sections, see nature.com/documents/nr-reporting-summary-flat.pdf

Life sciences study design

All studies must disclose on these points even when the disclosure is negative.

Sample size	58 cases coming from unique patients were chosen to demonstrate a proof of concept for the technique.
Data exclusions	Two of the cases used in the preliminary study of 16 samples were excluded from the final analysis, as WSI of the three special stains could not be obtained from serial tissue sections - and therefore could not be used for all three phases of the expanded study.
Replication	We reproduced the technique using the 58 subjects described in the paper.
Randomization	The 58 non-neoplastic kidney cases were randomly selected by a board-certified kidney pathologist (J.E.Z.) to represent a variety of kidney diseases. All training, validation, and test samples were randomly chosen and allocated.
Blinding	Blinding is not relevant - the diagnosticians were only given H&E to perform their first diagnosis, and for their second and third diagnoses (after the >3 week washout period) they were given all of the different generated stains as well as the H&E to test the study hypothesis.

Reporting for specific materials, systems and methods

We require information from authors about some types of materials, experimental systems and methods used in many studies. Here, indicate whether each material, system or method listed is relevant to your study. If you are not sure if a list item applies to your research, read the appropriate section before selecting a response.

Materials & experimental systems

n/a	Involved in the study	
☒	☐	Antibodies
☒	☐	Eukaryotic cell lines
☒	☐	Palaeontology and archaeology
☒	☐	Animals and other organisms
☐	☒	Human research participants
☒	☐	Clinical data
☒	☐	Dual use research of concern

Methods

n/a	Involved in the study	
☒	☐	ChIP-seq
☒	☐	Flow cytometry
☒	☐	MRI-based neuroimaging

Human research participants

Policy information about studies involving human research participants

Population characteristics	Cases were randomly chosen to represent a range of non-neoplastic kidney diseases.
Recruitment	Images were obtained from an existing private database of non-neoplastic kidney diseases at UCLA Health.
Ethics oversight	Oversight was approved by UCLA IRB 18-001029.

Note that full information on the approval of the study protocol must also be provided in the manuscript.
